# Sample-specific network analysis identifies gene co-expression patterns of immunotherapy response in clear cell renal cell carcinoma

**DOI:** 10.1016/j.isci.2025.113061

**Published:** 2025-07-05

**Authors:** Liangwei Yin, Pietro Traversa, Mohamed Elati, Yamir Moreno, Natalia Marek-Trzonkowska, Christophe Battail

**Affiliations:** 1 Université Grenoble Alpes, IRIG, Laboratoire Biosciences et Bioingénierie pour la Santé, UA 13 INSERM-CEA-UGA, 38000 Grenoble, France; 2 Institute for Biocomputation and Physics of Complex Systems (BIFI), University of Zaragoza, 50018 Zaragoza, Spain; 3 Department of Theoretical Physics, University of Zaragoza, 50018 Zaragoza, Spain; 4 CENTAI Institute, 10138 Turin, Italy; 5 University Lille, CNRS, Inserm, CHU Lille, UMR9020-U1277 – CANTHER – Cancer Heterogeneity Plasticity and Resistance to Therapies, F-59000 Lille, France; 6 International Centre for Cancer Vaccine Science, University of Gdansk, Kladki 24, 80-822 Gdansk, Poland; 7 Laboratory of Immunoregulation and Cellular Therapies, Department of Family Medicine, Medical University of Gdańsk, ul. Dębinki 2, 80-811 Gdańsk, Poland

**Keywords:** oncology, bioinformatics, machine learning

## Abstract

Immunotherapies have recently emerged as a standard of care for advanced cancers, offering remarkable improvements in patient prognosis. However, only a small subset of patients benefit, and robust molecular predictors remain elusive. We present a computational framework leveraging sample-specific gene co-expression networks to identify features predictive of immunotherapy response in kidney cancer. Our results reveal that patients with similar clinical outcomes exhibit comparable gene co-expression patterns. Notably, increased gene connectivity and stronger negative gene-gene associations are hallmarks of poor responders. We further developed sample-specific pathway-level network scores to detect dysregulated biological pathways linked to treatment outcomes. Finally, incorporating these sample-level network features improves the predictive performance of gene expression-based machine learning models. This work highlights the value of personalized gene network features for stratifying patients with cancer and optimizing immunotherapy strategies.

## Highlights

• Sample-specific gene networks predict the immunotherapy response of kidney cancer
• Poor responders exhibit high gene connectivity and strong negative gene-gene associations
• Network features of treatment response differ between primary tumor site and metastases
• Network features improve the performance of gene expression-based machine learning models

## Graphical abstract

**Figure:**
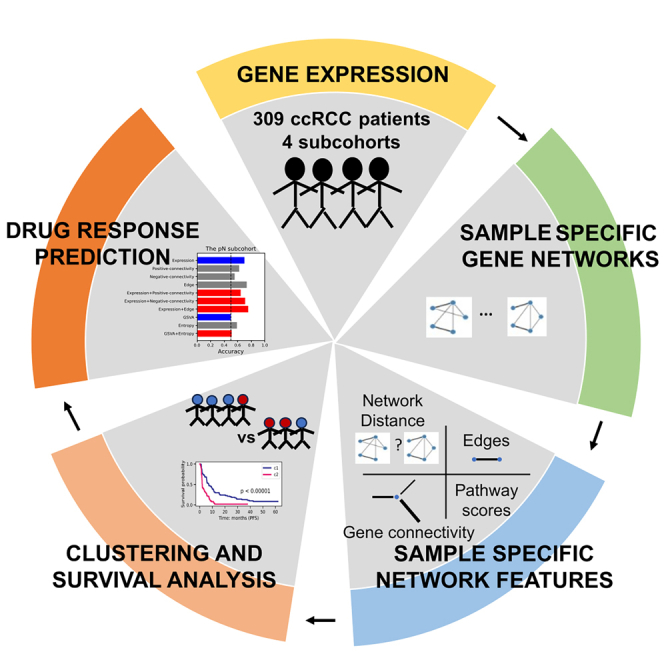

## Introduction

Clear cell renal cell carcinoma (ccRCC) is the predominant histological subtype of kidney cancer, with a high mortality rate following metastatic progression.^1^ Immune checkpoint inhibitors (ICIs) targeting programmed cell death protein 1 (PD-1) and programmed cell death ligand 1, either as monotherapy or in combination with angiogenesis inhibitors, have become the standard of care for metastatic ccRCC in recent years.^2^^,^^3^ While these therapies have improved patient survival rates, the objective response rate to nivolumab, an ICI, has been reported to be only 34.1%.^4^ Among the response mechanisms to ICIs, truncating mutations in polybromo-1 and focal loss of 10q23.31 have been positively associated with patient survival, likely due to the higher expression of angiogenesis genes and the loss of the tumor suppressor *PTEN*, respectively.^5^^,^^6^^,^^7^ Although immunotherapy aims to enhance immune response against tumors, the proportion of CD8^+^ T cell infiltration has not been correlated with treatment outcomes.^8^ However, these findings have not been consistently observed in prior studies,^9^^,^^10^^,^^11^ underscoring the complex mechanisms of genomic mutations and T cells in tumor progression and therapy resistance. Therefore, the identification of novel predictive markers is crucial for optimizing patient therapies and advancing personalized medicine.

To model the complex disease system at the individual level, several methods have been developed to infer sample-specific networks that capture the unique network structures of multiple samples with different phenotypes. These include the sample-specific network (SSN) method,^12^ which estimates perturbations of the Pearson correlation coefficient for each pair of genes; SSN based on the partial correlations between genes (P-SSN)^13^; linear interpolation for inferring SSN (LIONESS)^14^; Bayesian optimized networks obtained by assimilating omic data (BONOBO)^15^; and sample-specific weighted correlation network (SWEET),^16^ which mitigates size imbalances between different subpopulations of a dataset.

With regard to the key differences between these methods, the SSN method infers an SSN using one case sample against a set of control samples as a reference, based on differential Pearson correlation. SSNs have demonstrated strong performance on both simulated data and experimental tumor transcriptomes, highlighting their biological relevance in identifying deregulated pathways and driver genes.^12^^,^^17^ Similarly, the P-SSN method uses a differential partial correlation analysis between a set of control samples (m) and a specific sample plus the given samples (m + 1). By focusing on direct interactions and excluding indirect interactions, the P-SSN network distance can distinguish different cancer types or subtypes based on network edges.^13^ Both P-SSN and SSNs rely on a reference group of healthy samples, which may overlook the heterogeneity of patient samples across populations. To address this limitation, the LIONESS method uses linear interpolation to estimate sample-specific networks by comparing an aggregated network of a group (m) and a perturbed network without a case sample (m − 1).^14^ While LIONESS can be affected by population size, the SWEET method introduces genome-wide sample weights into network inference to mitigate this problem. These methods show that network degrees of PD-1 pathway genes and the *TBC1D* gene are associated with patient survival in glioblastoma and lung adenocarcinoma, respectively.^16^^,^^18^ Similarly, the BONOBO method constructs sample-specific co-expression networks without relying on external reference data and achieves gene network reconstruction performance on simulated data that are very similar to that of SWEET.^15^ While methods for inferring sample-specific gene co-expression networks do exist, no study has yet comprehensively extracted and analyzed network features to assess their relevance in precision medicine, particularly as predictive markers of treatment response in patients with cancer.

In our study, we inferred SSNs and extracted a wide range of network features to investigate the relevance of gene co-expression patterns in the stratification and treatment response of patients with ccRCC. From sample-specific weighted co-expression networks generated using the SWEET method, we explored not only network features such as gene connectivity and gene-gene associations but also network similarity and pathway network-based scores. These latter metrics account for the overall network structure to enable patient subtyping and integrate network information into signaling pathways. Using transcriptomic profiling data from 309 patients with advanced ccRCC collected in clinical trial cohorts, we stratified patients into distinct clusters and identified gene co-expression patterns associated with patient survival using network similarity, network nodes, network edges, and pathway network-based scores. The network features improved the prediction performance of gene expression score-based machine learning (ML) models. Additionally, we validated the relevance of pathway network-based scores in an independent cohort of patients with advanced ccRCC treated with avelumab and axitinib. In summary, our method not only provides a comprehensive strategy to explore gene co-expression patterns from general network structure to specific network markers for patient stratification and treatment prediction but also complements sample-specific pathway enrichment analysis in current cancer research.

## Results

### Inference of sample-specific gene co-expression networks

**Figure 1:**
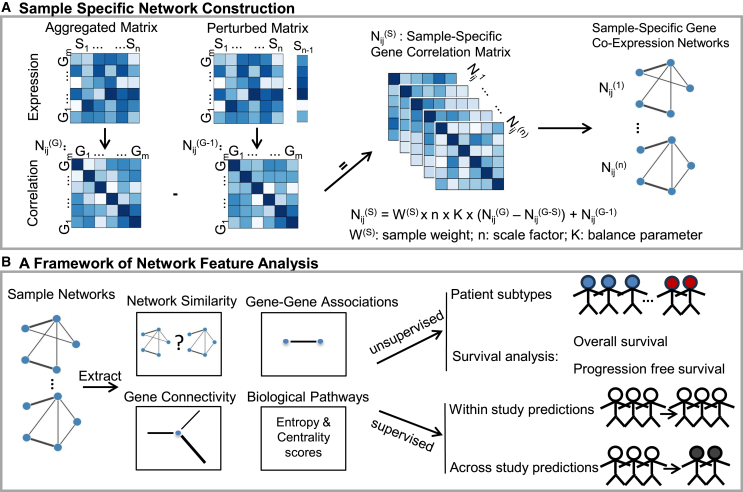
A computational framework for the inference of sample-specific gene co-expression networks and calculation of network features to stratify patients based on their response to antitumor therapies (A) Description of sample-specific gene network construction with the SWEET method. Each sample network was constructed with the difference between an aggregated correlation matrix and a sample-specific perturbed correlation matrix. Sample weight (*W*^*(S)*^), scale factor (*n*, i.e., number of samples), and balance parameter (*K*) were used to adjust for differences in proportions of sample subgroups within a cohort. *S*_n_: the *n*_th_ sample; *G*_n_: the *n*_th_ gene; *N*_ij_: the network of edges between gene i and gene j, i, j ∈ m (the number of genes); *N*_ij_^(G)^: aggregated network; *N*_ij_^(G−S)^: perturbed network; *N*_ij_^(S)^: sample specific network. (B) Description of our pipeline for patient subtyping, survival analysis, and treatment response predictions using network features calculated from sample-specific gene networks. Network similarity was measured by adjusted network distance. Gene connectivity and gene-gene edges were calculated using both the number and the strength of associations between genes. Biological pathway entropy and centrality scores embedded the complexity and the topology of gene network within each pathway.

Sample-specific weighted gene co-expression networks (ssGCNs) were constructed with 20,545 genes using the SWEET method for each subcohort from a meta-cohort of 309 patients with advanced ccRCC included in CheckMate 009, CheckMate 010, and CheckMate 025 clinical trials (Figure 1A).^8^^,^^16^ To accurately study differences in patient treatment response, the meta-cohort was divided into four subcohorts based on both the therapy administered and the site of tissue biopsy, either primary tumor or metastasis. Indeed, it has been shown that primary and metastatic sites in advanced kidney cancers harbored distinct molecular characteristics,^19^ which may influence the treatment response of patients. In details, these subcohorts consisted of 133 and 92 samples from primary tumor sites of patients treated with nivolumab (pN, anti-PD-1) or everolimus (pE, mammalian target of rapamycin [mTOR] inhibitor), respectively, and 47 and 37 samples from tumor metastases of patients treated with nivolumab (mN, anti PD-1) or everolimus (mE, mTOR inhibitor), respectively (Table S1). Utilizing an optimal balance parameter set at 10% and a two-sided *Z* score threshold of 2.58, the ssGCNs attained an average network density of 1.6%, encompassing 20,357 nodes and 3,320,160 edges, with an average determination coefficient *R*^2^ of 0.696 for scale-free topology (Figure S1). It is widely acknowledged that an *R*^2^ value closer to 1 indicates that the ssGCNs adhere more closely to the anticipated *power-law* node*-degree distribution* observed for biological networks. A further observation of note is that the *R*^2^ coefficients for SSNs from primary tumor sites exhibited higher values compared to those derived from tumor metastases (0.774 vs. 0.448, Wilcoxon rank-sum test, *p* value < 0.01) (Figure S2A). To assess whether subcohort size influenced the *R*^2^ coefficient of the gene-degree distribution, a simulation was performed on the pN subcohort by randomly selecting 40–120 samples. The results showed that a decrease in cohort size was associated with a reduction in the mean of *R*^2^ coefficients, likely reflecting a loss of robustness of the gene network (Figure S2B).

Cancers often exhibited varied gene network complexities, with acquired network nodes demonstrating enrichment in metabolic and immune-related processes including regulation of immune response, T cell receptor signaling pathway, and podosome assembly.^20^ To explore whether our ssGCNs exhibited tumor-specific features, we compared their network density and enrichment of cancer-related genes to an aggregated network from expression data of normal renal cortex tissues (*n* = 85). The obtained results demonstrated that the ccRCC ssGCNs exhibited a higher network density (1.6%) in comparison to the normal network (0.46%). Furthermore, 98.3% (304/309) of our ssGCNs demonstrated a higher enrichment of cancer-related genes among the top 1,000 genes of the highest degree when compared to the normal network (Figure S3). These characteristics validate the relevance of our ccRCC SSNs for further exploration using advanced network features.

To identify novel network-based markers for predicting immunotherapy response, we focused on the pN and mN subcohorts of patients treated with nivolumab. The pE and mE subcohorts were used primarily to validate the biological relevance of network features.

### Adjusted network distance in Pearson correlation-based ssGCNs

Network distance, a measure of similarity, has been utilized to estimate gene regulation similarity between samples and accurately identify tumor subtypes.^13^ To assess whether network distance could reflect clinical status similarity, we calculated pairwise network distances between ssGCNs of patients in the pN subcohort (Equation 3). However, unsupervised clustering based on these network distances showed no significant association with survival data (Figure S4; log rank tests: *p* value > 0.2 for both overall survival [OS] and progression-free survival [PFS]). The limited sensitivity of network distance may be attributed to the relatively small divergence between gene networks of patients with differing immunotherapy responses compared to those distinguishing tumor subtypes.

To address this limitation, we developed an adjusted version of network distance incorporating clinical outcome categorization collected from the Braun et al. publication (clinical benefit [CB], intermediate clinical benefit [ICB], and nonclinical benefit [NCB]). While this categorization was defined by objective responses, tumor shrinkage, and PFS,^8^ the adjusted network distance did not directly rely on PFS values, ensuring unbiased subsequent analyses. Adjusted distances were computed using edges between each sample and an aggregated network constructed based on clinical outcomes. For the pN and mN subcohorts, CB-aggregated networks were derived from 44 and 13 samples, resulting in 2,450,498 and 720,447 edges, respectively. NCB-aggregated networks were constructed from 46 and 20 samples, yielding 2,853,404 and 1,758,449 edges, respectively. Three versions of adjusted network distances were computed relative to the CB network, the NCB network, and the difference between the two.

**Figure 2:**
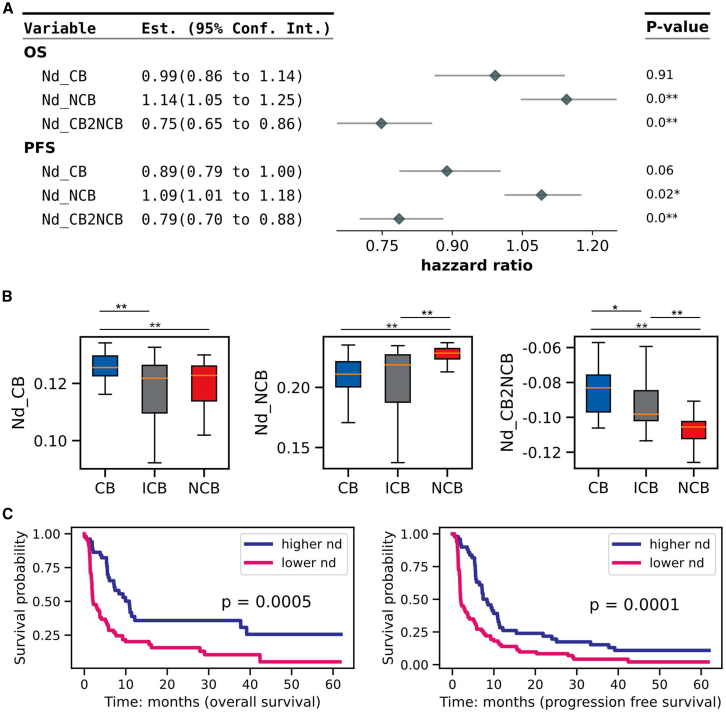
Survival analysis with the adjusted network distance calculated on the subcohort of tumor primary sites from patients followed after immunotherapy by nivolumab (A) A forest plot depicting the univariate Cox regression results using adjusted network distances. Sample-specific network distances were adjusted with aggregated networks of clinical benefit (CB), nonclinical benefit (NCB), or the difference between them. (B) Comparison of the distributions of adjusted network distances between CB categories using Wilcoxon rank-sum tests. (C) Survival analysis using network distances adjusted with the differences between CB- and NCB-aggregated networks. Samples were divided into two groups based on the median value of the adjusted network distance (nd) (higher nd and lower nd groups). *p* values were calculated using the log rank tests. (∗∗: *p* value < 0.01; ∗: *p* value < 0.05).

Univariate Cox regression analysis revealed a correlation between adjusted network distances and survival data (Figure 2A). For the pN subcohort, network distances adjusted to the CB-aggregated network were favorable for OS and PFS, though not significantly. In contrast, distance adjusted with NCB-aggregated networks was significantly unfavorable for OS and PFS (the Cox model: *p* value < 0.05). Adjusted distances using both CB- and NCB-aggregated networks demonstrated more pronounced favorable associations with OS and PFS. A comparison between CB and NCB patients revealed that CB patients had significantly higher network distances when adjusted with CB or both CB and NCB networks and lower distances when adjusted with NCB networks alone (Figure 2B; Wilcoxon rank-sum test; *p* value < 0.01). Survival analysis based on adjusted network distances showed that patients with higher distances were significantly associated with greater OS and PFS (Figure 2C; log rank tests, *p* value < 0.01). Furthermore, adjusted network distances correlated with OS for the mN subcohort and with both OS and PFS for the pE subcohort (Figures S5A–S5C). In summary, these results demonstrate that within the context of ssGCNs generated from Pearson correlations, network distance adjusted with prior clinical knowledge effectively stratifies patients based on their response to nivolumab.

### Gene connectivity is associated with treatment response

**Figure 3:**
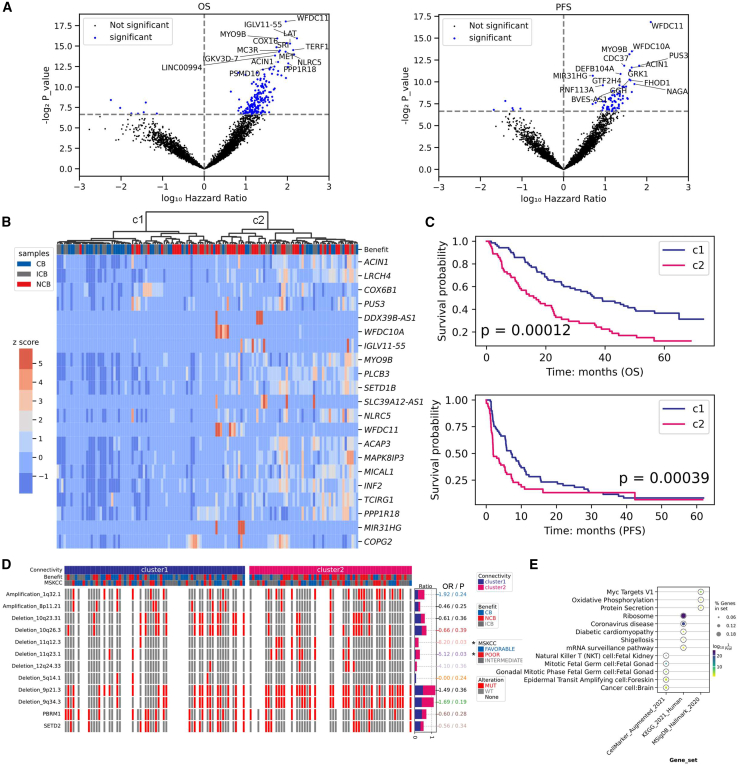
Survival and exploratory analysis using gene connectivity (positive correlation) calculated on the subcohort of tumor primary sites from patients followed after immunotherapy by nivolumab (A) Volcano plots of gene connectivity association with patient overall survival (OS) and progression-free survival (PFS) (Cox’s proportional hazards model, *p* value < 0.01). (B) Unsupervised hierarchical clustering of samples into two groups (c1: 29 CB, 24 ICB, and 17 NCB patients and c2: 15 CB, 19 ICB, and 29 NCB patients) based on the connectivity of 21 genes significantly associated with both OS and PFS. Of these 21 genes, expression values of 8 genes (“*SLC39A12-AS1*,” “*WFDC10A*,” “*MYO9B*,” “*TCIRG1*,” “*WFDC11*,” “*MIR31HG*,” “*DDX39B-AS1*,” and “*IGLV11-55*”) were also associated with survival data. (C) Survival analysis between the clusters c1 (blue) and c2 (pink) of samples. *p* values were calculated using the log rank tests. (D) Distribution of chromosomal mutations and gene mutations between the two clusters c1 and c2. Fisher’s test was conducted, and *p* values less than 0.05 were considered as significant (odds ratio [OR] was provided). MSKCC refers to Memorial Sloan Kettering Cancer Center prognostic model, widely used for outcome prediction of treatments. Chromosome gain and loss were counted for chromosomal mutations, and somatic point mutations (including splice sites, frameshift, missense, nonsense, and in-frame indel) were counted for gene mutation. (E) Gene ontology over-representation analysis. Genes were selected as the union of significant genes whose connectivity was associated with OS or PFS. Gene sets from KEGG, MSigDB hallmark, and Cellmarker databases were used. For each pathway, the color of each circle represents the adjusted *p* value, and the size of circles indicates the percentage of selected genes in the gene sets.

Gene degree, or connectivity, has been linked to cancer subtypes and survival outcomes.^12^^,^^16^ In weighted networks, gene connectivity refers to the sum of connection weights with other genes. We hypothesized that gene connectivity could differentiate patient treatment responses. To test this, we separately generated gene connectivity matrices from positive and negative correlations of ssGCNs to prevent the mixing of opposing associations. From positive correlation matrix, we identified 21 genes whose connectivity was significantly associated with OS and PFS in the pN subcohort (Figure 3A; Cox model: *p* value < 0.01; Table S2). Notably, only 8 of these genes were also linked to survival based on their expression values. Using 21 genes’ connectivity, unsupervised clustering divided samples into two groups, including cluster 1 (29 CB, 24 ICB, and 17 NCB patients) and cluster 2 (15 CB, 19 ICB, and 29 NCB patients) (Fisher’s exact test: *p* value = 0.0203). Cluster 2 exhibited higher gene connectivity on average (Figure 3B) and significantly worse survival probability for both PFS and OS compared to cluster 1 (Figure 3C; log rank tests: *p* value < 0.01). Next, we investigated whether genomic mutation and clinical features differed between the two clusters. Cluster 2 had significantly higher frequencies of chromosomal losses at 11q12.3 and 11q23.1, along with greater intratumor heterogeneity (ITH) (Figure 3D; Figure S6; Fisher’s exact test: *p* value < 0.05). Notably, 11q23 deletion has been linked with poor prognosis in several cancers,^21^^,^^22^ and ITH has been associated with tumor progression and response to immunotherapy.^23^ Gene ontology analysis revealed that the 21 highly connected genes were enriched in cancer- and immune-related pathways such as MYC targets v1, oxidative phosphorylation, ribosome pathways, and natural killer (NK) T cell gene set (a cell population predictive of the response to anti-tumor treatments) (Figure 3E).^24^^,^^25^ Among these genes, *NLRC5* and *PLCB3*, known for their involvement in ccRCC progression and tumor immunity, were also identified.

A similar analysis of gene connectivity from negative correlations identified 48 significant genes, where greater connectivity was associated with worse survival in cluster 2 (Figure S7). Notably, clustering based on positive and negative correlation-derived connectivity overlapped substantially, with 46 patients common between the two cluster 1 groups (70 and 56 patients) and 53 patients common between the two cluster 2 groups (63 and 77 patients) (Figure S7G). To study the relevance of these findings in tumor metastases, we analyzed the mN subcohort and obtained 9 and 17 genes (based on positive and negative associations, respectively) whose higher connectivity was significantly associated with lower OS and PFS (Table S2; Figures S8A and S8B). Interestingly, no overlap was found between these genes and those identified from the pN subcohort.

The same approach was applied to the pE and mE subcohorts. Consistently, higher gene connectivity correlated with worse survival outcomes, and clustering patterns were similar between positive and negative correlation-derived connectivity (Figures S9 and S10).

Overall, these findings demonstrate that the increase in gene connectivity—whether from positive or negative gene-gene correlations—is associated with poorer prognosis and shorter treatment recurrence. Moreover, the genes linked to survival or recurrence varied depending on the tumor’s primary or metastatic location.

### Highly negative gene-gene associations in patients without CBs

**Figure 4:**
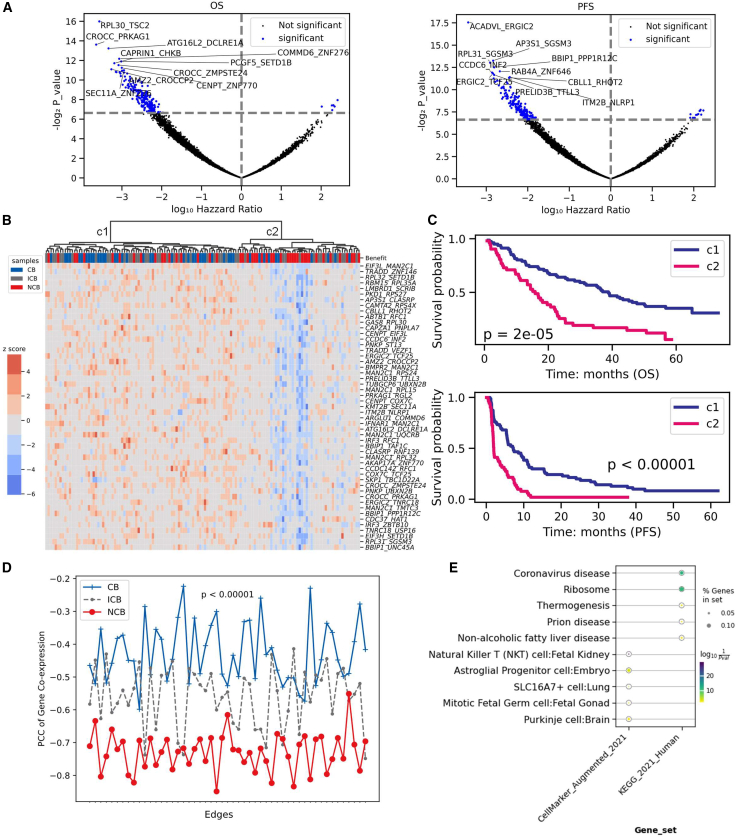
Survival analysis of gene-gene associations using the subcohort of tumor primary sites from patients followed after immunotherapy by nivolumab (A) Volcano plots of gene-gene weight association with overall survival (OS) and progression-free survival (PFS) (Cox’s proportional hazards model, *p* value < 0.01). (B) Unsupervised hierarchical clustering of samples into two groups (c1: 34 CB, 29 ICB, and 18 NCB patients; c2: 10 CB, 14 ICB, and 28 NCB patients) using 51 edge weights significantly associated with both OS and PFS. (C) Survival analysis between the clusters c1 (blue) and c2 (pink) of samples. *p* values were calculated using the log rank tests. (D) Distributions of Pearson correlation coefficients (PCCs) for pN samples from the clinical benefit (CB, blue), intermediate clinical benefit (ICB, gray), and nonclinical benefit (NCB, red) groups. The Wilcoxon rank-sum test was conducted between CB and NCB patients. (E) Gene ontology overrepresentation analysis. Genes were selected as the union of significant genes whose edge weight was associated with OS and PFS. Gene sets from KEGG, MSigDB hallmark, and Cellmarker databases were used. For each pathway, the color of each circle represents the adjusted *p* value, and the size of circles indicates the percentage of selected genes in the gene sets.

Investigating gene pairwise associations in sample-specific networks has become a popular method for identifying cancer subtypes.^13^^,^^26^ To assess perturbations in gene-gene associations, we first focused on edges shared by all samples. In ssGCNs of the pN subcohort, we obtained 238,804 edges and filtered them based on variance, retaining the top 10,000 most varied edges. Among these, 214 and 224 significant edges were significantly associated with OS and PFS values, respectively (Cox model: *p* value < 0.01), with 51 edges intersecting between both (Figure 4A; Table S3). A small subset of genes from these edges overlapped with those previously identified based on connectivity (Figure S11). Unsupervised clustering of these edges revealed that cluster 2 (10 CB, 14 ICB, and 28 NCB patients) exhibited stronger negative gene associations (Figure 4B) and was significantly associated with worse OS and PFS compared to cluster 1 (34 CB, 29 ICB, and 18 NCB patients; Fisher’s exact test: *p* value = 0.00065; Figure 4C; log rank test: *p* value < 0.01). However, no significant differences in genomic mutation or clinical features were observed between these clusters (Figure S12).

Further analysis of edge weights across patient categories revealed that NCB patients exhibited stronger negative correlations compared to CB patients (Figure 4D; Wilcoxon rank-sum test: *p* value < 0.01). It raised the question of whether transcription factors (TFs) regulated genes from these edges. Enrichment analysis identified three significant TFs (*MZF1*, *ZNF692*, and *RBCK1*), previously associated with cancer progression and prognosis in ccRCC (Table S4).^27^^,^^28^^,^^29^ Genes from these edges associated with both OS and PFS were overrepresented in ribosome pathways and NK T cell gene sets (Figure 4E), with some known cancer-related genes (*PRELID3B*-*TTLL3*, *BMPR2*-*MAN2C1*, etc.) among them.^30^^,^^31^^,^^32^

In the mN subcohort, six edges were significantly associated with both OS and PFS values (Table S3). Clustering based on these edges identified cluster 2 with significantly shorter OS and PFS (Figures S13A and S13B). Similarly, 5 of these edges were highly negatively co-expressed in NCB patients compared with CB patients (Figure S13C). Functional enrichment analysis revealed similar pathway involvement as observed in the pN subcohort, while no significant differences in somatic mutation and clinical features were observed between the two clusters (Figures S13D–S13F).

In the pE and mE subcohorts, the same approach identified 40 and 10 significant edges, respectively, which also stratified samples into two clusters with distinct survival outcomes (Figures S14A and S14B). A visualization of the selected edges across all four subcohorts is provided (Figure S15). Overall, highly negative gene-gene associations were linked to poor prognoses. Notably, the genes from significant edges weakly overlapped (46.9% for pN) with those identified based on their connectivity, indicating that edges and gene connectivity offer complementary insights into patient survival predictions. Furthermore, differences in significant edges between primary and metastatic tumor sites underscore the necessity of tumor site-specific analyses in cancer research.

### Pathway entropy and centrality scores

Given that both gene connectivity and gene-gene associations revealed differences in patient response to treatments, we next asked whether the complexity and topological features of biological pathways were also associated with patient survival. A previous study has shown that the complexity of signaling pathways is linked to survival in pan-cancer molecular data.^33^ To explore this, we developed a tool to calculate topology-based pathway scores at the sample-specific level. This tool first extracted pathway networks from our ssGCNs using Kyoto Encyclopedia of Genes and Genomes (KEGG) gene sets, followed by the computation of pathway entropy and centrality scores. While entropy measures the randomness or complexity of a network, eigenvector centrality reflects the transitive influence of genes, closeness centrality indicates the average shortest distance from one gene to the other, and edge betweenness centrality measures the influence of edges within a network.

**Figure 5:**
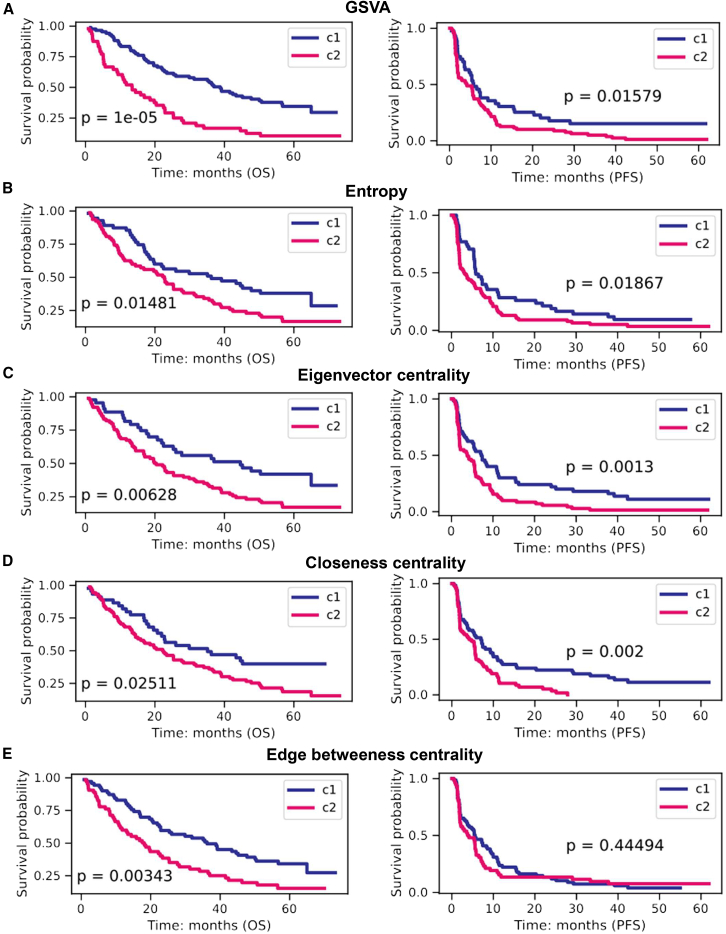
Survival analysis of biological pathway entropy and centrality scores using the subcohort of tumor primary sites from patients followed after immunotherapy by nivolumab Several survival analyses were carried out with separation of the patient cohort according to topological pathway scores: use of 12 (OS) and 5 (PFS) significant pathways based on GSVA scores (A), use of 28 (OS) and 7 (PFS) significant pathways based on entropy scores (B), use of 8 (OS) and 10 (PFS) significant pathways based on gene eigenvector centrality scores (C), use of 16 (OS) and 3 (PFS) significant pathways based on gene closeness centrality scores (D), and use of 8 (OS) and 10 (PFS) significant pathways based on edge betweenness centrality scores (E). *p* values were calculated using the log rank tests.

To assess whether these pathway scores captured specific biologically meaningful information, we identified pathways significantly associated with OS or PFS values using the Cox model (*p* value < 0.05). We applied the same method to select significant pathways based on gene set variation analysis (GSVA) scores, but notably, there was little overlap between pathways identified by topology-based scores and those derived from GSVA using gene expression values (Figure S16). We then used the significant pathway scores to stratify patients into two clusters to assess their predictive power (Figure S17). In the pN subcohort, all categories of pathway scores successfully clustered patients according to OS (log rank test: *p* value < 0.05) (Figure 5, left). The most robust classifications were achieved with GSVA and edge betweenness centrality scores. For treatment response, all pathway score categories except edge betweenness centrality were significantly associated with PFS (Figure 5, right; log rank test: *p* value < 0.05), with eigenvector centrality showing the highest significance. Consistent with the patterns of gene connectivity and edges, patients with better survival exhibited lower entropy scores (Figure S17B).

Regarding the biological pathways involved, some were consistently associated with survival across multiple score categories, while others were identified by only one type of score. The phosphatidylinositol pathway, known to play a central role in ccRCC,^34^ was notably found by all scores, except GSVA. The erythroblastic oncogene B (ERBB) signaling pathway, having a vital role in the initiation and progression of ccRCC,^35^ was identified by GSVA and the eigenvector centrality score. Regarding specific pathways, the eigenvector centrality score revealed some of the main pathways associated with the pathogenesis of kidney cancer, such as mTOR, ascorbate and aldarate metabolism, unsaturated fatty acids, and mismatch repair.^36^^,^^37^^,^^38^^,^^39^ Additionally, the circadian rhythm pathway linked to ccRCC prognosis was identified by the closeness centrality score.^40^

The relevance of pathway entropy and centrality scores in predicting survival or recurrence was further confirmed in the mN, pE, and mE subcohorts (Figures S19 and S20). Overall, our findings demonstrate that pathway topology features provide complementary prognostic insights beyond conventional gene expression-based enrichment methods. The observed differences between topology-based and GSVA-derived pathways underscore the importance of integrating network-based approaches for a more comprehensive understanding of treatment response in ccRCC.

Combining network features and gene expression values in ML models better predicts immunotherapy response.

**Figure 6:**
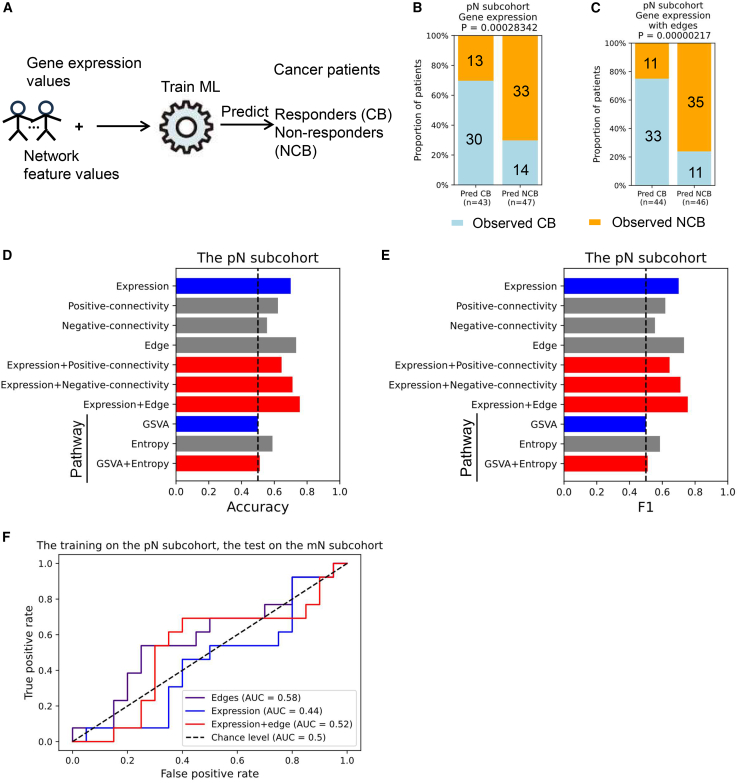
Prediction of drug response for immunotherapy-treated patients using gene expression values and network features (A) Overall scheme of LOOCV predictions of a logistic regression ML model (class weight was set to “balanced”) based on gene expression value and network features as the input. (B–E) Predicted responders (Pred CB) and predicted nonresponders (Pred NCB) are plotted against real responders (light blue) and nonresponders (orange). The two-sided Fisher’s exact test was used to compute statistical significance. Gene expression values were used as the input (B). The combination of expression values and edges was the input (C). The accuracy (D) and F1 scores (E) were computed for gene expression (blue), network features (gray), and their combinations (red). (F) Across study prediction. The pN subcohort (*n* = 90) was used as the training set (30 features selected for both edges and expression, 50 features for their combination during the training process), and the mN subcohort was used as the test set (*n* = 33). The area under the curve (AUC) of the receiver operating characteristics curve was used here as a performance metric. We assumed that the pN subcohort shared biological mechanisms partially with the mN subcohort.

Gene network information from existing databases can enhance survival predictions in patients with cancer.^41^ We set out to investigate whether SSN features could improve the performance of gene expression-based ML models in predicting immunotherapy response (Figure 6A). For this analysis, we focused on 90 samples from the pN subcohort, excluding ICB patients, and classified 44 CB patients as responders and 46 NCB patients as nonresponders.

Using logistic regression (LR) classification models (Figure S21 for model comparison, default hyperparameters) with leave-one-out cross-validation (LOOCV), we assessed predictive performance using gene expression, network features, and their combinations. The best-performing model combined gene expression and edges, achieving an accuracy of 0.755 and an F1 score of 0.75 (Figures 6B–6E, Fisher’s exact test, *p* value < 0.05; Figure S22 for feature selection; Figure S23; Tables S5 and S6). While gene expression alone provided a reasonable prediction (Table S6; accuracy: 0.7), integrating edges improved performance (accuracy: 0.755). Pathway entropy-based models also outperformed GSVA-based models (Figures 6D and 6E). This trend held across support vector machine model and deep neural network model, though not for random forest (RF) models (Figure S24). We noted that hyperparameter tuning did not generally improve the prediction performance (Tables S7 and S8).

To test model generalizability, we trained LR and RF models (default hyperparameters) on the pN samples and tested the prediction performance on the mN samples (33 patients with 13 responders and 20 nonresponders), assuming that pN patients and mN patients partially shared biological mechanisms in immunotherapy treatment response. We compared the predictive performance of treatment response models based on expression or edge features alone and the combined expression and edge feature set using the area under the curve of the receiver operating characteristics curve (Figures 6F and S25). We observed that the best performance in the across-study approach was achieved using edge features alone, followed by the expression and edge combination, and finally by expression features alone (hyperparameter tuning did not generally improve the prediction performance as presented in Figure S25). As transcriptomic profiles are known to differ substantially between primary tumor sites and metastases,^19^^,^^42^ we anticipated a global decrease in the performance of the ML models. This was indeed observed when comparing results from the across-study evaluation to the LOOCV performance obtained independently on either primary or metastatic tumor data. Nevertheless, this across-study training-test set design suggests that edge features capture unique aspects of gene regulation from the primary tumor site that are also predictive of treatment response at metastatic sites.

These findings suggest that integrating sample-level network features with gene or pathway markers could enhance ML model performance in predicting responses of patients with ccRCC to immunotherapy.

### Validation of pathway entropy and centrality scores in another cohort

**Figure 7:**
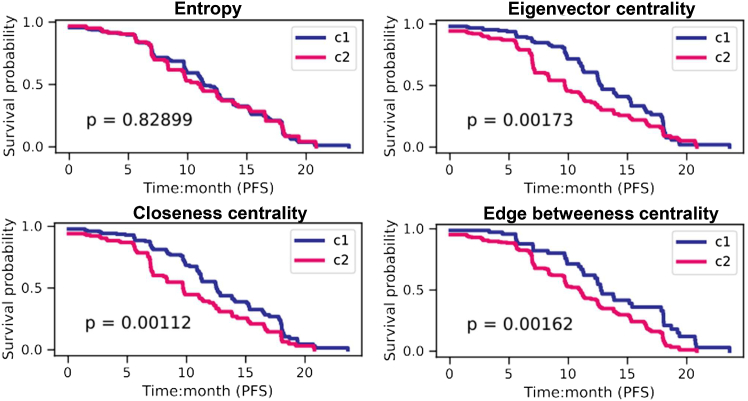
Survival analysis using biological pathway entropy and centrality scores calculated using an independent patient cohort from Mozter et al. Clusters c1 (blue) and c2 (pink) of samples were defined using an unsupervised hierarchical clustering of pathway scores significantly associated with patient PFS values. *p* values were calculated using the log rank tests.

Having shown the ability of sample-specific pathway scores to predict the susceptibility of patients to nivolumab, we extended this analysis to an independent cohort of 354 patients with ccRCC followed after treatment with the therapeutic combination avelumab and axitinib (an immune checkpoint inhibitor and antiangiogenic therapy).^43^ Following the same approach used for the pN cohort, we inferred sample-specific gene networks and calculated pathway scores to cluster samples into two groups (Figure S26). Respectively with eigenvector, closeness, and edge betweenness scores, clustering revealed a significant difference in treatment response (PFS) between two clusters, using 10, 5, or 6 significant pathways (Figures 7 and S26). These pathways were already known to be deregulated in ccRCC such as those associated with the metabolism of amino acids and fatty acids or those involved with DNA repair.^44^^,^^45^^,^^46^^,^^47^ While no significance in survival was found with pathway entropy, the consistent divergence of metabolic pathways between clusters suggests a key role in ccRCC progression (Figure S26).

To conclude, we validated the relevance of topology-based pathway scores derived from ssGCNs in an independent cohort of patients with ccRCC treated with combination immunotherapy. These scores effectively stratified patients, reinforcing their potential as predictive biomarkers.

## Discussion

Detection of regulatory perturbations in sample-specific gene networks inferred from expression data has contributed to progress in personalized medicine by refining the stratification of patients into cancer subtypes.^13^^,^^14^^,^^16^^,^^26^^,^^48^ Our study introduced an innovative computational framework for SSN analysis, facilitating the use of multiscale network features in cancer research. Our approach enabled a refined characterization of gene networks for patients with ccRCC and revealed distinct gene co-expression patterns linked to immunotherapy response.

Network similarity facilitates the comparison of gene networks, allowing for the clustering of networks that group patients with similar gene regulation patterns. While network distance has been effective in identifying tumor types in partial correlation-based networks,^13^ we detected a lack of sensitivity when applying it to Pearson correlation-based networks to classify patients based on treatment responses. To enhance its efficacy, we improved network distance by comparing one sample to a selected group of patients with known medical outcomes. Our adjusted network distance revealed a high-resolution way to associate each sample-specific co-expression network with the patient’s clinical outcome.

Specific network markers such as node degree and edge weight further showed that higher gene connectivity and stronger negative gene pairwise associations were prevalent in patients with poorer survival probability and worse treatment response. This finding suggests that heightened activity within cancer-associated networks reflects dysregulated gene interactions that promote tumor progression, rather than effective biological response. Notably, the observed increase in connectivity and edges was observed only in a subset of differential networks between patients, rather than in complete networks derived for patients. This distinction is crucial, as it indicates that the observed changes are specific to tumor-associated activity instead of general biological activity. Supporting this, many genes identified as network markers in our study have been previously implicated in tumor progression, metastasis, and resistance to antitumor treatments. Additionally, changes in these network features were associated with deletion_11q12.3 and deletion_11q23.1, and ITH in the subcohort pN, which were previously linked to cancer progression and prognosis.^21^^,^^22^^,^^23^ Furthermore, an increase in connectivity and co-expression may indicate changes in the tumor microenvironment, as they are enriched in NK T cells.^24^^,^^25^ This suggests that network-based markers capture distinct biological processes beyond traditional differentially expressed genes, providing novel and complementary predictors of treatment response in patients with ccRCC.

The topology of gene co-expression networks, characterized by varied gene connectivity and edges, could also be examined through the lens of biological pathway networks. Entropy-based measures of signaling pathways have already been proposed to assess their activation state for survival analysis in pan-cancer studies.^49^ To extend this concept, we calculated sample-specific entropy and topology-based pathways scores, alongside the classic GSVA pathway score, to identify pathway co-expression perturbation significantly associated with treatment response. While some pathways were consistently identified by several scoring methods, others were unique to a specific score, illustrating the complementary nature of these approaches. Concerning pathway deregulation linked to nivolumab response, the eigenvector centrality score was effective for samples from primary and metastatic sites of ccRCC tumors, supplemented by the closeness and the edge betweenness centrality scores for metastases. Our findings suggest that entropy and topological pathway scores offer diverse and valuable perspectives to detect clinically relevant pathways, capturing complexity change or structural changes in the pathway co-expression network.

Traditional gene expression analysis also has the capacity to distinguish patients in treatment response, and we compared its predictive performance with gene connectivity and edges. Using the same method, we selected 64 genes for the pN subcohort and 65 genes for the mN subcohort based on expression values and performed unsupervised clustering (Figure S26; log rank tests: pN-OS, *p* value = 0.00015, pN-PFS, *p* value = 0.02559; mN-OS, *p* value = 0.00367; mN-PFS, *p* value = 0.00037). When comparing gene expression with gene connectivity or edges, we observed that edges exhibited higher predictive performance in the pN subcohort (Figure 4C), whereas gene connectivity was more predictive in the mN subcohort (Figures S8A and S8B). Furthermore, these network features not only demonstrated their ability in predicting patients’ CBs but also enhanced the prediction performance of gene expression-based ML models. Specifically, incorporating edges into gene expression-based ML models increased its prediction accuracy from 0.70 to 0.75. Although our pathway entropy provided a slight improvement over GSVA-based ML model due to the limited number of selected pathways, their predictive effectiveness was validated using another independent dataset. Consistent with other network-based prediction models,^50^^,^^51^ our results suggest that combining SSN features with gene expression enables more accurate predictions of patient response to immunotherapy. Nevertheless, the robustness and generalizability of our integrated ML models will need to be confirmed in additional independent patient cohorts.

In terms of potential novel predictors of treatment response in patients with ccRCC, we observed a substantial discrepancy in these lists of genes carrying co-expression patterns between primary and metastatic sites, aligning with previous studies that reported significant changes in expression profiles between primary tumor sites and metastases.^19^^,^^42^ Furthermore, through pathway network analysis, we revealed that the deregulations of the ccRCC primary site associated with PFS values were rather captured by the phosphatidylinositol, ERBB, and mTOR signaling pathways and by the ascorbate and aldarate metabolism and the mismatch repair system, while those of metastases were linked to cytokine inflammation, sphingolipid metabolism, aminoacyl-tRNA biosynthesis, and citrate-tricarboxylic acid (TCA) cycle pathways, as well as the nucleotide excision process. These findings underscore the site-specific co-expression patterns and biological pathways that are differentially associated with patient survival depending on whether the sample originates from a primary or metastatic site, consistent with the diversity of molecular characteristics observed between primary and metastatic sites in advanced kidney cancers.^19^ This specificity suggests the critical role of tumor site location in disease progression and treatment response, emphasizing the importance of considering the biopsy or surgery site in the development of predictive models and therapeutic strategies for ccRCC.

In conclusion, our approach demonstrates sample-specific gene co-expression network features as alternative markers to predict survival and treatment response in patients with advanced kidney cancer. It also enables the identification of perturbed pathways based on sample-specific networks and facilitates the routine application of sample-specific pathway network-based scores. Most importantly, our computational framework for investigating gene network features linked to patient treatment responses provides a valuable tool to support the personalization of therapies in the clinic.

### Limitations of the study

Our approach has several limitations and perspectives. The sample size of clinical cohorts is limited, and detailed network markers observed from our analysis need to be further validated in other independent clinical drug trials and ultimately for different cancer types and treatments. Also, the small sample size may cause overfitting issue in the training process for ML models. Moreover, our approach may require the control of the heterogeneity of samples in a group to maintain common edges for identifying specific edges. Also, our evaluation of the predictive potential of pathway-based scores within ML models, as presented in Figures 6D, 6E, S21, S23, and S24, should be considered preliminary, as it was limited to GSVA (expression based) and entropy (network topology based) scores. Our primary objective was to demonstrate that a pathway-based score capturing gene network topology could complement traditional expression-based scores. However, future work should focus on the optimal selection and integration of diverse pathway-based scores to develop more effective ML models for predicting treatment outcomes. Furthermore, potential confounding variables such as age, sex, and prior treatments were not adjusted for feature selections, and the difference in age was significantly detected between two clusters based on edges in the pN subcohort (Figure S27; 63.22 vs. 57.86; Student’s t test: *p* = 0.007). Finally, the biological meaning of co-expression patterns needs to be further defined. The change in co-expression networks from bulk RNA sequencing (RNA-seq) data may be influenced by the cellular content of the tumor immune microenvironment, tumor heterogeneity, or intercellular communication. The advent of single-cell RNA-seq datasets from patient tumors may provide an opportunity to explore network change at the scale of cellular subtypes to unravel intracellular factors from those in the microenvironment sources driving changes in co-expression.

## Resource availability

### Lead contact

Further information and requests for resources and reagents should be directed to and will be fulfilled by the lead contact, Christophe Battail (christophe.battail@cea.fr).

### Materials availability

This study did not generate new unique reagents.

### Data and code availability

• Data: processed data of gene expression used in this study can be found in the Zenodo repository: https://doi.org/10.5281/zenodo.15723817.
• Code: all code to this article is available in the Zenodo repository: https://doi.org/10.5281/zenodo.15723817.
• Other items: any additional information required to re-analyze the data in this article is available from the lead contact upon request.

## Acknowledgments

Part of the computations were carried out using the GRICAD infrastructure (https://gricad.univ-grenoble-alpes.fr), which is supported by Grenoble research communities. The authors also appreciate the valuable suggestions of Dr. Guilherme Ferraz de Arruda and Dr. Alberto Aleta on the network construction method. This work was supported by the KATY project, which has received funding from the European Union’s Horizon 2020 research and innovation program under grant agreement no. 101017453, by the CANVAS project, which has received funding from the 10.13039/100018693Horizon Europe twinning program under grant agreement no. 101079510, and by the DIGPHAT project (Multi-scale and longitudinal data modeling in pharmacology: toward digital pharmacological twins), which has received funding from the French research initiative “France 2030” through the program PEPR Digital Health under ANR grant agreement no. 22-PESN-0017. This work was also supported by the IDEX outgoing international mobility grant of 10.13039/100012952Université Grenoble Alpes.

## Author contributions

L.Y. and C.B. conceived the study. L.Y. developed and performed the analysis. L.Y. and C.B. wrote the manuscript. P.T. contributed to the methodologies. N.M.-T., M.E., and Y.M. contributed to the revision of the manuscript.

## Declaration of interests

The authors declare no competing interests.

## STAR★Methods

### Key resources table

**Table:** 

REAGENT or RESOURCE	SOURCE	IDENTIFIER
**Deposited data**		

Analyses, and resources related to gene network for ccRCC patients	This paper	Zendo: https://doi.org/10.5281/zenodo.15723817
ccRCC Bulk RNA-seq	Braun et al.^8^	N/A
Normal kidney cortex tissue Bulk RNA-seq	GTEx	https://www.gtexportal.org/home/downloads/adult-gtex/bulk_tissue_expression
Cancer-related Genes	Cancer Gene Census^52^	https://cancer.sanger.ac.uk/census#cl_search
ccRCC Bulk RNA-seq	Motzer et al.^43^	N/A

**Software and algorithms**		

SWEET	Chen et al.^16^	https://github.com/SysMednet/SWEET
Network Distance	Huang et al.^13^	N/A
Numpy	Numpy	https://numpy.org/news/#releases
Pandas	PyData	https://pandas.pydata.org/
Seaborn	PyData	https://seaborn.pydata.org/
Matplotlib	Matplotlib	https://matplotlib.org/
Lifelines	Lifelines	https://lifelines.readthedocs.io/en/latest/
scikit-survival	scikit-survival	https://scikit-survival.readthedocs.io/en/stable/
PyComplexHeatmap	PyComplexHeatmap	https://github.com/DingWB/PyComplexHeatmap
Gseapy	GSEAPY	https://gseapy.readthedocs.io/en/latest/introduction.html
ChEA3	ChEA3	https://maayanlab.cloud/chea3/
GSVA	BiocManager	https://github.com/rcastelo/GSVA
Igraph	Conda	https://python.igraph.org/en/stable/
Venny4Py	Venny4Py	https://github.com/timyerg/venny4py
Sklearn	Scikit-learn	https://scikit-learn.org/stable/
ccRCC Bulk RNA-seq	Motzer et al.^43^	N/A

### Experimental model and study participant details

This study did not involve the recruitment of human participants or animal models. All analyses were conducted using publicly available datasets. Details of the data sources, including sample characteristics and preprocessing methods, are provided in the STAR Methods, method details and data and code availability. The original datasets, obtained from the Braun et al.,^8^ and Motzer et al.,^43^ had been previously approved for public use by the respective data providers.

### Method details

#### Data collection

Gene expression, genomic mutation, and clinical data of 309 ccRCC patients were obtained from the publication by Braun et al. (CheckMate 009, 010, 025).^8^ Clinical outcomes collected from the Braun et al. study classified patients into three categories: Patients with objective responses (complete or partial), or stable disease with tumor shrinkage and PFS of at least 6 months were classified as having clinical benefit (CB); Patients with progressive disease and PFS less than 3 months were classified as having no clinical benefit (NCB); All other patients were classified as intermediate clinical benefit (ICB). Sequencing data were generated prior to treatment, and survival data was collected after treatment. The expression data of these samples were split into four groups based on the treatment and cancer site in patients (Table S1). Ensembl gene ID was converted to gene symbol using a human gene annotation file (release v43) from the GENCODE database version 43^53^ and only immune genes, mitochondrial genes, long non-coding RNA genes, and protein-coding genes were kept for gene network inference. To validate the constructed networks, RNA-seq data from 85 normal kidney cortex tissue samples were obtained from the GTEx portal, and cancer-related genes were collected from the Cancer Gene Census database.^52^ Additionally, data from an independent ccRCC cohort were downloaded from the publication by Motzer et al.,^43^ including expression profiles of 354 patients treated with avelumab (anti PD-L1) and axitinib (anti-angiogenic). Gene expression values were normalized using the log_2_ transformation of transcripts per million (TPM).

#### Gene network inference

In this study, we used the recently developed SWEET method to construct sample-specific weighted gene co-expression networks (ssGCNs).^16^ An aggregated network (*N*_*ij*_^*G*^) was first constructed using gene expression of all samples within a specific category. Subsequently, a perturbed network (*N*_*ij*_^*G-S*^) was generated by removing one specific sample from the aggregated network (Figure 1A). Specifically, a sample-specific network $N_{ij}^{\left(S\right)}$ was defined as

(Equation 1) $$N_{ij}^{\left(S\right)}=W^{\left(S\right)}\times num\times K\times \left(N_{ij}^{\left(G\right)}-N_{ij}^{\left(G-S\right)}\right)+N_{ij}^{\left(G-S\right)}$$

where num was the total number of samples except the target sample in a group, $W^{\left(S\right)}$ was the sample weight, and K was a balance factor ranging from 0 to 1. The best performance was achieved with K = 10% as shown in Figure S1 and by the SWEET paper. The parameter K was a scale factor used to enlarge the differential correlation between the aggregated matrix and the perturbed matrix. The sample weight $W^{\left(S\right)}$ was added to the equation of sample-specific networks to neutralize the network edge number bias.^16^ $W^{\left(S\right)}$ was calculated as

(Equation 2) $$W^{\left(S\right)}=\frac{\left(\mu_{PCC}^{\left(S_{P}\right)}-\min\left({PCC}^{S}\right)+x\right)}{\left(\max\left({PCC}^{S}\right)-\min\left({PCC}^{S}\right)+x\right)}$$

where $\mu_{PCC}^{\left(S_{P}\right)}$ was the mean of Pearson Correlation Coefficient (PCC) between one specific sample S and the other samples, ${PCC}^{S}$ was the set of PCCs between two patients and *x* was a constant term added to avoid division by zero that we set to 0.01. To reduce the noise within the networks, the significance level of confidence scores for edges was assessed using *Z* score normalization, with a *Z* score threshold of 2.58, corresponding to a two-sided *p*-value of 0.01.

#### Network distance

Network distance (*Nd*) was introduced by Huang et al. as a measure of network similarity, primarily used to identify cancer types.^13^ *Nd* was defined as the ratio of the number of overlapped edges to the number of union edges between two partial correlation-based sample networks,

(Equation 3) $$Nd=\frac{\left(E_{i}\cap E_{j}\right)}{\left(E_{i}\cup E_{j}\right)}$$

where $E_{i}$ and $E_{j}$ represented the sets of edges from sample-specific networks. These edges correspond to direct interactions between genes.

However, due to the difference between Pearson correlation and partial correlation, network distance may not be suitable for subtyping patients when using Pearson correlation-based gene networks. Befitting the divergence of treatment response, we proposed an adjusted network distance, which calculated the similarity between an individual sample network and an aggregated network of a specific group. Adjusted distances were calculated using clinical benefits (CB) patients (Equation 4) or non-clinical benefits (NCB) patients aggregated networks (Equation 5). Also, the difference between CB distance and NCB distance was used for further analysis (Equation 6).

(Equation 4) $${Nd}_{cb}=\frac{\left(E_{i}\cap E_{cb}\right)}{\left(E_{i}\cup E_{cb}\right)}$$

(Equation 5) $${Nd}_{ncb}=\frac{\left(E_{i}\cap E_{ncb}\right)}{\left(E_{i}\cup E_{ncb}\right)}$$

(Equation 6) $$\Delta Nd=\frac{\left(E_{i}\cap E_{cb}\right)}{\left(E_{i}\cup E_{cb}\right)}-\frac{\left(E_{i}\cap E_{ncb}\right)}{\left(E_{i}\cup E_{ncb}\right)}$$

#### Network features

We explored two key network features: gene connectivity (weighted node degree) and gene-gene association (edges) (Figure 1B). Gene connectivity, which quantifies the total strength of all the associations of one gene with other genes, was calculated using the Python package igraph (version 0.10.4).^54^ To accurately identify whether the source of gene connectivity was from positive or negative regulations, networks were divided into two: one containing positive correlation edges and the other containing negative correlation edges. To reduce the computation time, the analysis was limited to the top 5,000 most varied genes based on their connectivity.

To assess the impact of edges, we focused on common edges across all samples within a cohort and selected the top 10,000 most variable edges based on their weights. Furthermore, genes and edges were filtered using a univariate Cox regression model, retaining those with a *p*-value less than 0.01. To align with the definition of clinical benefits, network features were preserved if they were significantly associated with both overall survival (OS) and progression free survival (PFS).

#### Pathway entropy and topology scores

Sample-specific pathway networks were extracted from sample networks using gene sets of pathways from the KEGG database.^55^ To enable the calculation of pathway scores, both positive and negative edge values (converted to their absolute values) were considered. Given that the noise elimination within ssGCNs led to a varying number of edges, pathway entropy was calculated based on the distribution of edges^56^ as follows:

(Equation 7) $$H=-\sum_{k=1}^{m}p\left(k\right)\log_{2}\left(p\left(k\right)\right)$$

where *p(k)* is the probability of an edge inside a selected pathway network, and *m* is the total number of edges inside that pathway network. The probability *p(k)* of each edge was determined by dividing the weight of the edge by the sum of all the edge weights inside the pathway network.

Additional pathway topological scores were calculated based on the average of gene eigenvector centrality, gene closeness centrality, and edge betweenness centrality.^57^ Alongside our entropy and topology scores, sample-level pathway scores based on gene expression were calculated using the gene set variation analysis (GSVA 1.46.0) method implemented in R (version: 4.2.3).^58^

#### Clustering of samples and survival analysis

Unsupervised hierarchical clustering was conducted using the Ward method and cosine metrics. To facilitate comparisons of treatment responses, we divided the samples into two clusters. To assess survival probability between clusters, Kaplan-Meier curves were plotted, and log rank tests were performed to determine whether the survival distribution of the two clusters were significantly different with a *p* value of 0.05. These analyses were conducted using the Python packages lifelines 0.27.7 and scikit-survival 0.21.0.^59^^,^^60^

#### Gene sets and transcription factor (TF) enrichment analysis

The Molecular Signatures Database (MSigDB) hallmark, the KEGG canonical pathways, and the cell marker (augmented 2021) were obtained from the Human MSigDB website and Enrichr libraries.^55^^,^^61^^,^^62^ For over-representation analysis, significant pathways were identified using the Python package gseapy 1.0.5^63^ and an adjusted *p*-value threshold of 0.05 was selected as a threshold. For the enrichment of transcription factors (TF), an online query was conducted on the CHEA3 online tool.^64^

#### Measuring the performance of machine learning (ML) predictions

To evaluate the prediction performance based on gene expression and network feature values, we used the Logistic Regression (LR) model, and also tested Random Forest (RF) model, Support Vector Classifier (SVC) models, and deep neural network (DNN) model.For leave-one-out cross-validation prediction, we used selectKbest function with f_classif parameter to select best features in the training process, and evaluated the performance using accuracy and F1 score. For gene expression, network features, and their combinations, the number of features (K) was selected based on prediction accuracy of models with K from 10 to 100 with a 10 step. For across study validation, we used the pN subcohort as the training set and used the mN subcohort as the test set, and the area under the receiver operating characteristic curve (AUC) was used as the main performance metric.

For the combination of gene expression and network features, gene expression matrix was horizontally merged with network features before feature selection in the training process. GSVA pathway matrix was merged with pathway entropy scores horizontally as well. These combined features were selected by selectedKbest function in the training process. We assumed that selected features from different categories could be complementary to the other to improve the performance of ML models. For hyperparameter tuning in LR models, we conducted 5-fold cross-validation in a training dataset with C ranging from 0.1 to 1 with a step of 0.1, and used GridSearchCV^65^ to identify optimal hyperparameters. The Grid range for other models can found in the Table S8. All analyses were implemented in Scikit-learn in Python.^65^

#### Validation of pathway scores

To assess whether our pathway scores were effective in other cohorts, we selected the data from 354 ccRCC patients treated with the combination of avelumab (anti-PD-L1) and axitinib (anti-angiogenic).^43^ For each sample in this cohort, sample-specific gene networks were constructed and pathway networks were then derived as described above.

### Quantification and statistical analyses

Sample specific networks were constructed using the SWEET method, and network features were extracted and generated accordingly. For adjusted network distance, the Cox model was used to test the association between the distance and survival data. For gene connectivity, edges, and pathway scores, the Cox model was used to filter out irrelevant features, and those features associated with both OS and PFS were used as the input of clustering using the function clustermap from the Python package seaborn. We performed Fisher’s exact test and computed the *p* value to observe the distribution of patients in two clusters. We also compared the distribution of somatic mutations between two clusters using Fisher’s exact test and compared the clinical features between two clusters using wilcoxon rank-sum test. Gene set enrichment analysis was performed using the function enrichr from the Python package GSEAPY. The specific test is mentioned in each context.
