# Supplementary material for: Sample-specific network analysis identifies gene co-expression patterns of immunotherapy response in clear cell renal cell carcinoma
Source: iScience. 2025 Jul 5;28(8):113061. doi: 10.1016/j.isci.2025.113061 (PMC12309972; doi:10.1016/j.isci.2025.113061)
Supplement: Document S1. Figures S1–S28 and Tables S1–S8 [file mmc1.pdf]

iScience, Volume *28*

Supplemental information

Sample-specific network analysis identifies gene co-expression patterns of immunotherapy response in clear cell renal cell carcinoma

Liangwei Yin, Pietro Traversa, Mohamed Elati, Yamir Moreno, Natalia Marek-Trzonkowska, and Christophe Battail

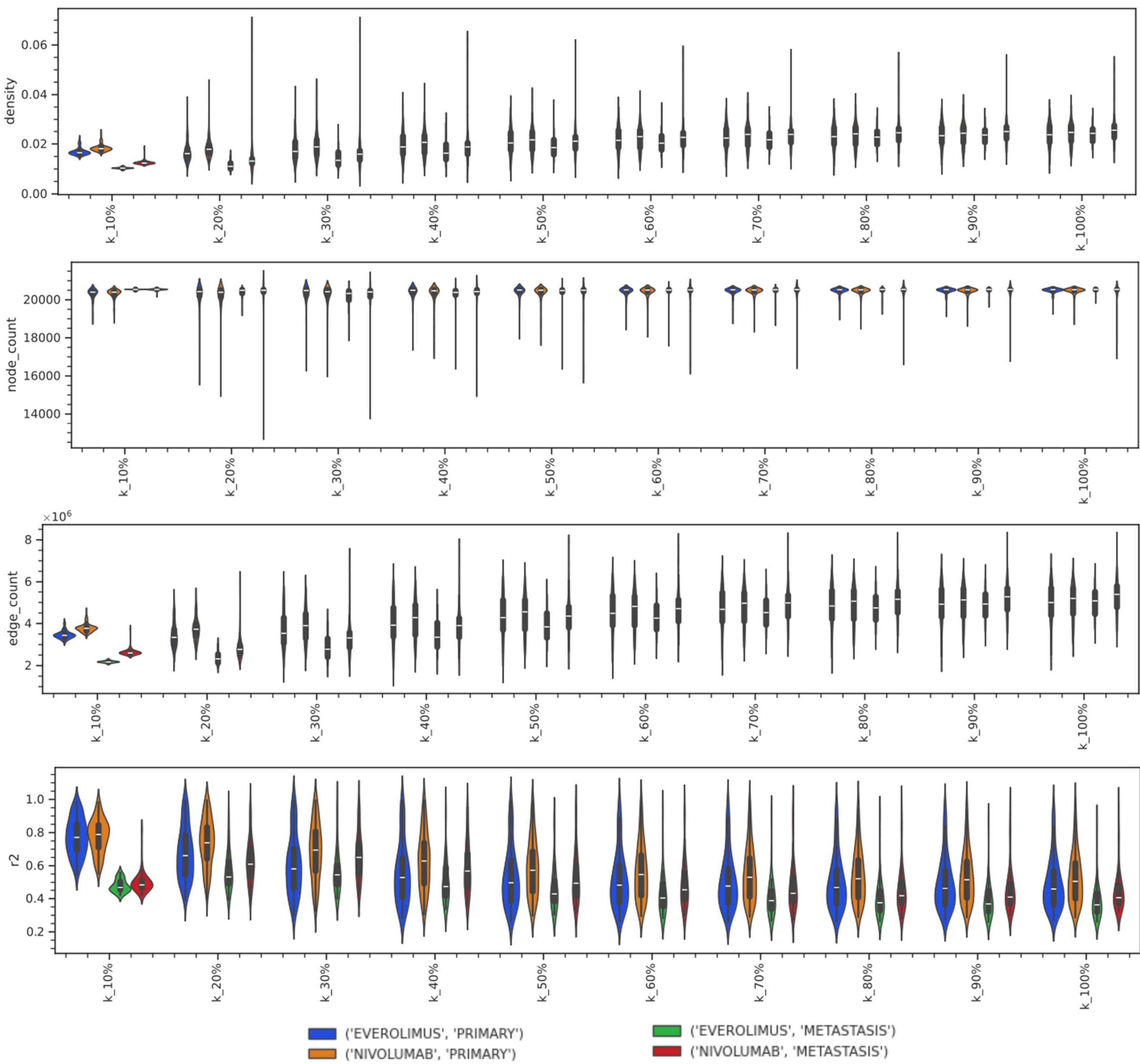

Figure S1. Network characteristics of ssGCNs constructed with different balance parameter k (from 0.1 to 1) in our four subcohorts, related to Figure 1. Network density, number of nodes, number of edges, and the fitness of scale free topology were present above. When k was at 10%, ssGCNs achieved the best network density and determination coefficients R^2 of scale free topology, which would be more similar to realistic biological network. Specifically, when k was set to 10%, ssGCNs of pE, pN, mE, nE achieved 1.68%, 1.83%, 1.03%, 1.25% network density, 20,273, 20,302, 20,538, 20302 node count, 3,458,841, 3,782,518, 2,176,807, 2,640,410 edge count and 0.772, 0.776, 0.477, 0.496 R^2 coefficients. The 10% k was used from all the downstream analysis.

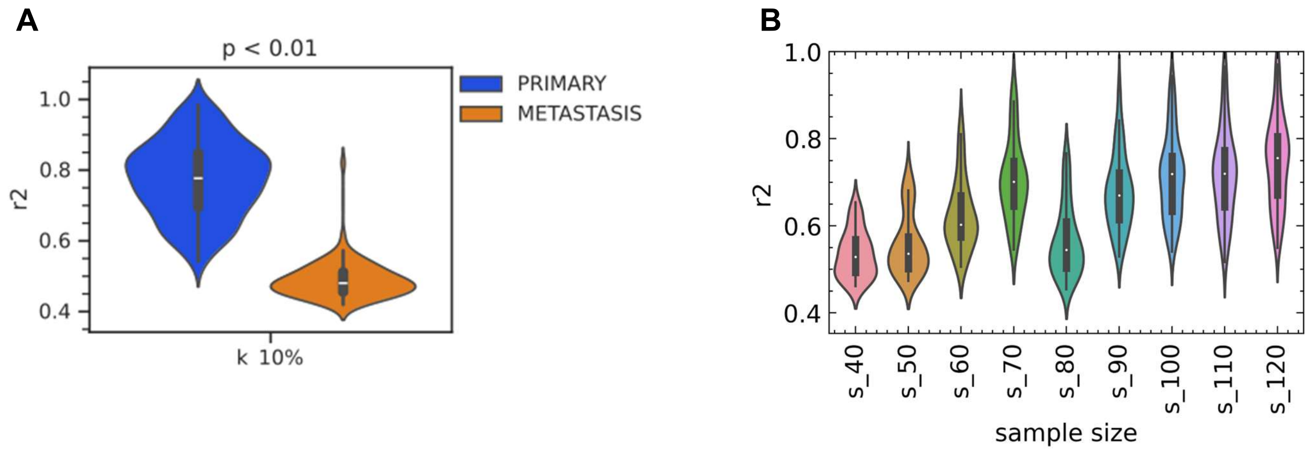

Supplementary Figure S2. Analysis of the influence of cohort sample size on the scale free topology nature of networks using the pN group, related to Figure 1. (A) The comparison of R^2 of ssGCNs between metastasis tumor in primary sites and metastasis. (B) The distribution of R^2 for ssGCNs constructed from different group size. The nature of scale free topology for networks was accessed by determination coefficient R^2. The closer R^2 is to 1, the degree distribution of a network follows a power law. Here, we used the biggest subcohort pN as the stimulation cohort to do the test. For different sample size, ssGCNs were constructed with a set number of randomly picked samples from pN and then R^2 of these ssGCNs was calculated. This figure provided that R^2 of network achieved higher values with larger group sample size.

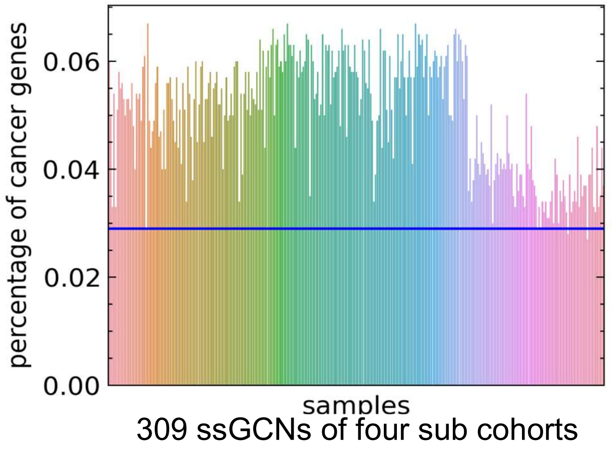

Figure S3. Enrichment analysis of cancer related gene in top 1000 high connectivity genes between normal samples and our tumor samples, related to Figure 1. Cancer related genes were extracted from the Cancer Gene Census database. The blue line indicates the percentage (2.9%) of cancer related gene in the top 1000 nodes of kidney cortex network. Out of our 309 ssGCNs, 304 ssGCNs were found to have a higher enrichment of cancer related genes inside their networks.

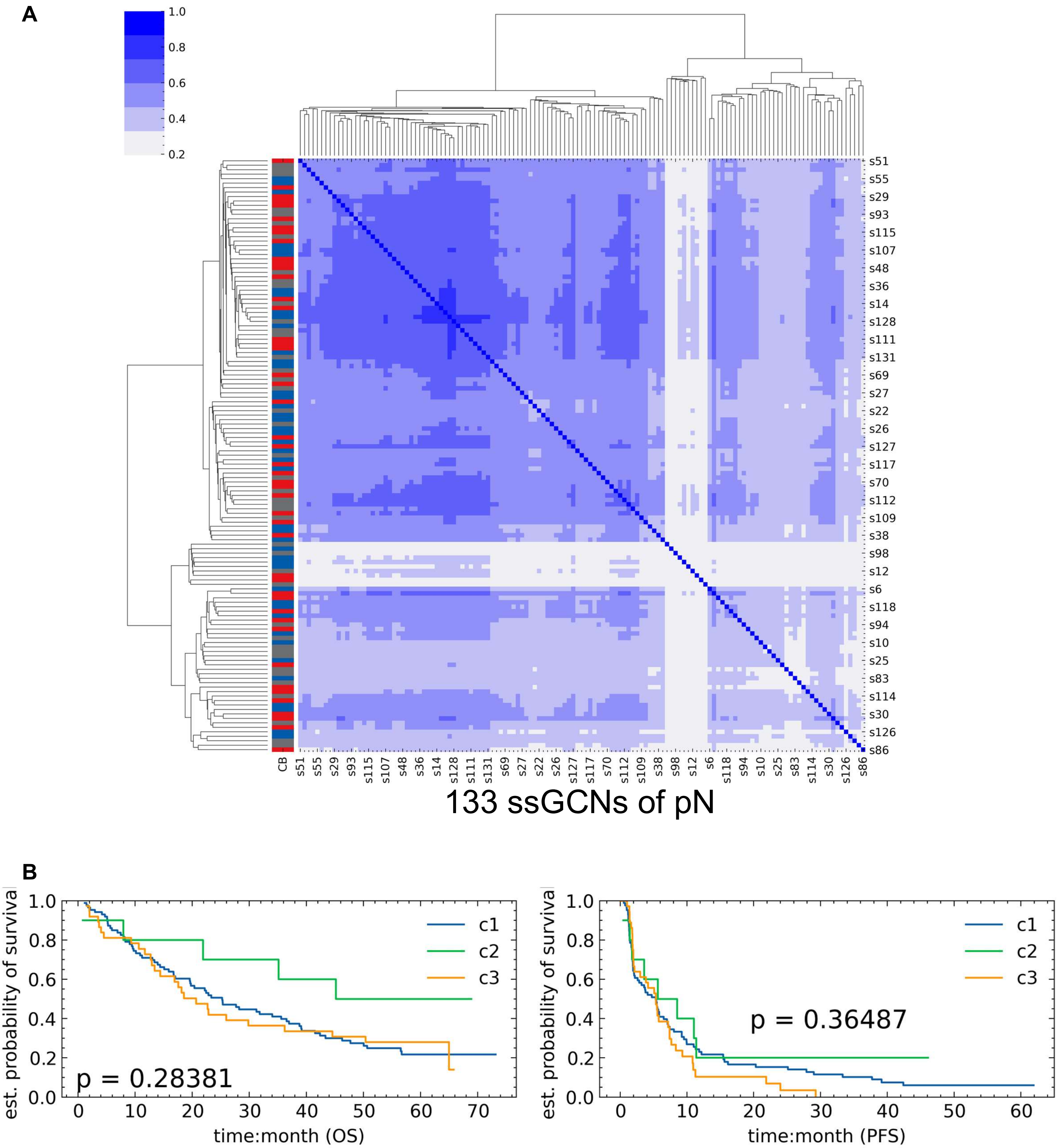

Figure S4. Patient clustering using network distance between samples of pN and survival analysis, related to Figure 2. (A) Patient clustering of pN samples. Similarity matrix, calculated by network distance, was used as the basis of hierarchical clustering. (B) Survival analysis. Three clusters were inferred from the clustering result above and their survival probability were measured using the log rank test (p values were obtained here).

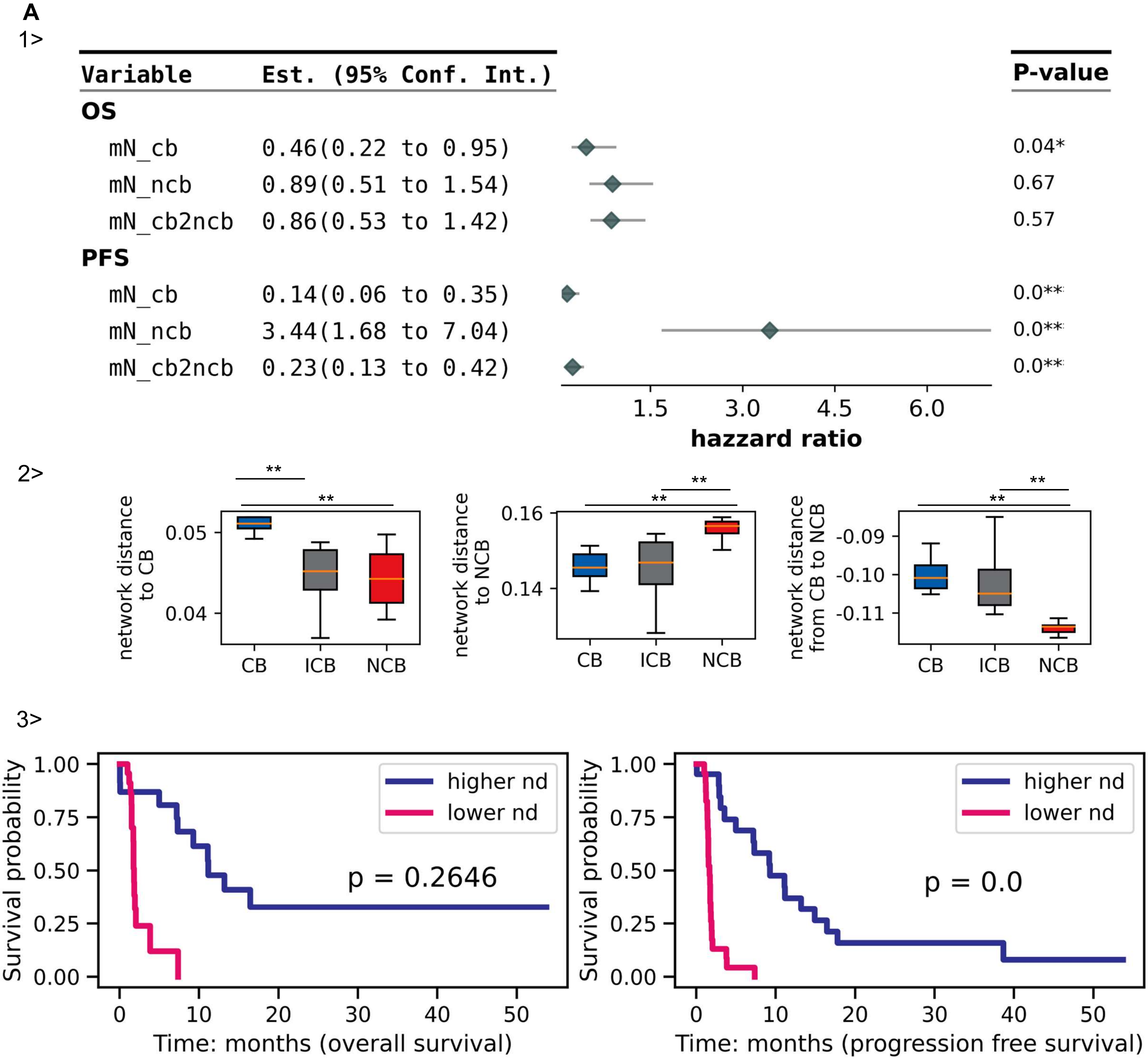
A
1>
Variable
Est. (95% Conf. Int.)
P-value
OS
mN_cb 0.46(0.22 to 0.95) 0.04*
mN_ncb 0.89(0.51 to 1.54) 0.67
mN_cb2ncb 0.86(0.53 to 1.42) 0.57
PFS
mN_cb 0.14(0.06 to 0.35) 0.0**
mN_ncb 3.44(1.68 to 7.04) 0.0**
mN_cb2ncb 0.23(0.13 to 0.42) 0.0**
1.5
3.0
4.5
6.0
hazzard ratio
2>
network distance to CB
0.05
0.04
**
**
CB
ICB
NCB
network distance to NCB
0.16
0.14
**
**
CB
ICB
NCB
network distance from CB to NCB
-0.09
-0.10
-0.11
**
**
CB
ICB
NCB
3>
Survival probability
1.00
0.75
0.50
0.25
0.00
higher nd
lower nd
p = 0.2646
0
10
20
30
40
50
Time: months (overall survival)
Survival probability
1.00
0.75
0.50
0.25
0.00
higher nd
lower nd
p = 0.0
0
10
20
30
40
50
Time: months (progression free survival)

B

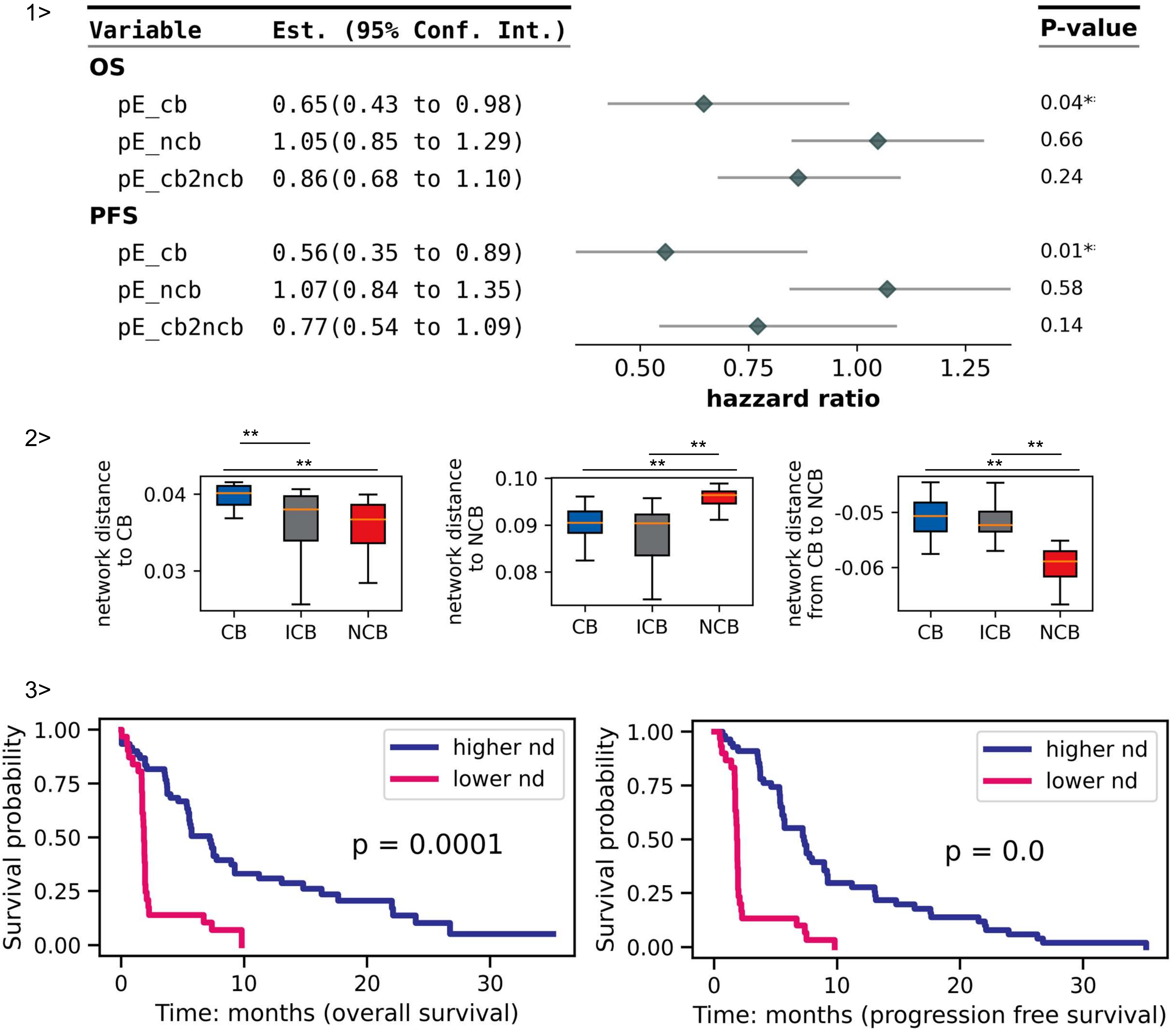
1>
Variable
Est. (95% Conf. Int.)
P-value
OS
pE_cb 0.65(0.43 to 0.98) 0.04*
pE_ncb 1.05(0.85 to 1.29) 0.66
pE_cb2ncb 0.86(0.68 to 1.10) 0.24
PFS
pE_cb 0.56(0.35 to 0.89) 0.01*
pE_ncb 1.07(0.84 to 1.35) 0.58
pE_cb2ncb 0.77(0.54 to 1.09) 0.14
0.50
0.75
1.00
1.25
hazzard ratio
2>
network distance to CB
0.04
0.03
CB
ICB
NCB
**
network distance to NCB
0.10
0.09
0.08
network distance from CB to NCB
-0.05
-0.06
3>
Survival probability
1.00
0.75
0.50
0.25
0.00
higher nd
lower nd
p = 0.0001
0
10
20
30
Time: months (overall survival)
p = 0.0
Time: months (progression free survival)

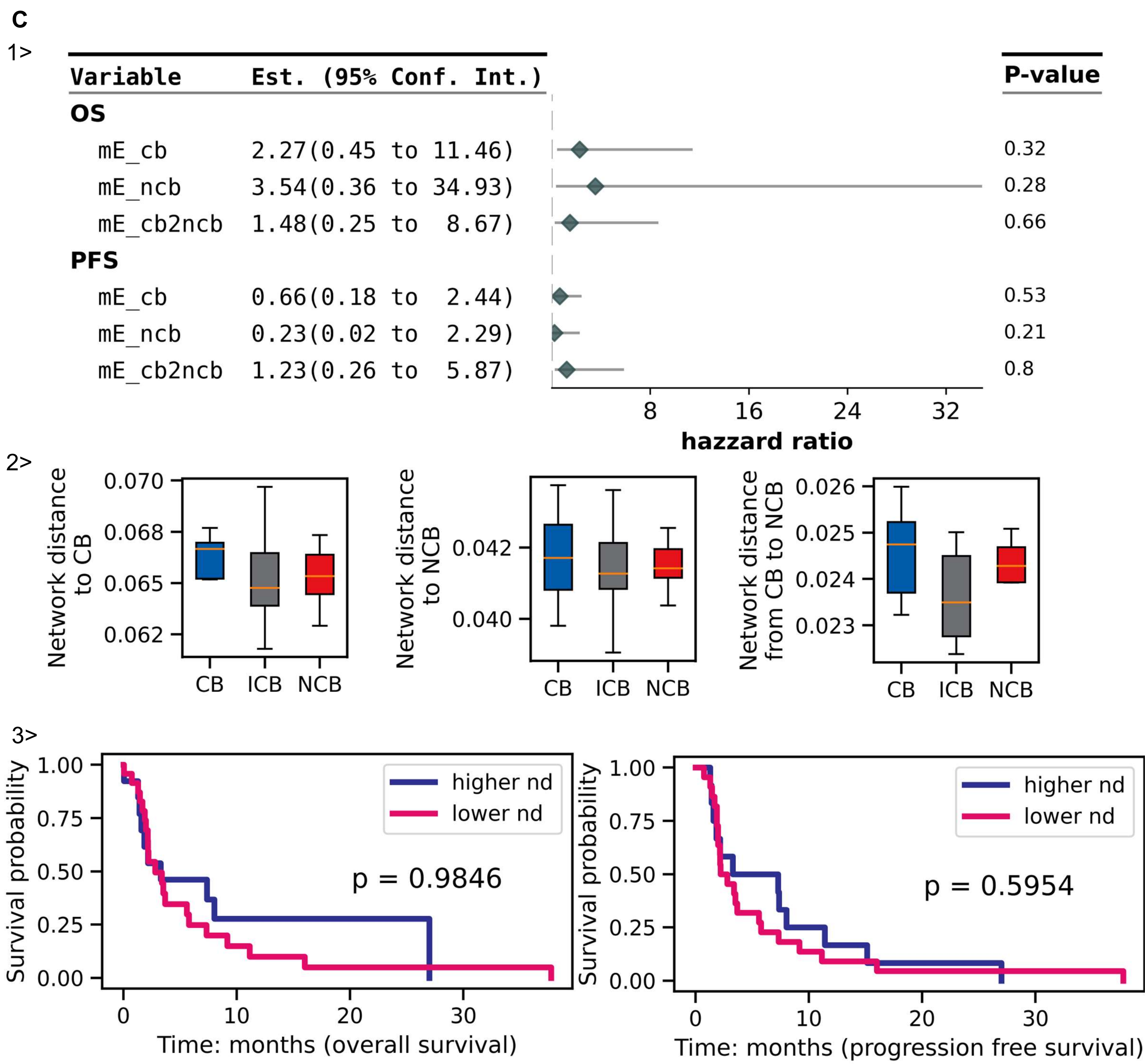

Figure S5. Adjusted network distance in mN (A), pE (B), mE (C), related to Figure 2.

1>. A forest plot of the univariate cox regression result using adjusted network distance. 2>. Comparison of adjusted network distance between samples with or without clinical benefits. Wilcoxon sum rank test was conducted. 3>. Survival analysis using network distance adjusted with CB to NCB. P value is from the log-rank test. Survival analysis using network distance adjusted with CB to NCB. Samples were divided into two groups (higher nd and lower nd groups) based on the median value of network distance adjusted with CB to NCB. P value is from the log-rank test. (**: p value < 0.01; *: p value < 0.05)

Noted that mN, mE subcohorts have smaller sample size (47, 37), which may lead to that adjusted network distance was not effective here.

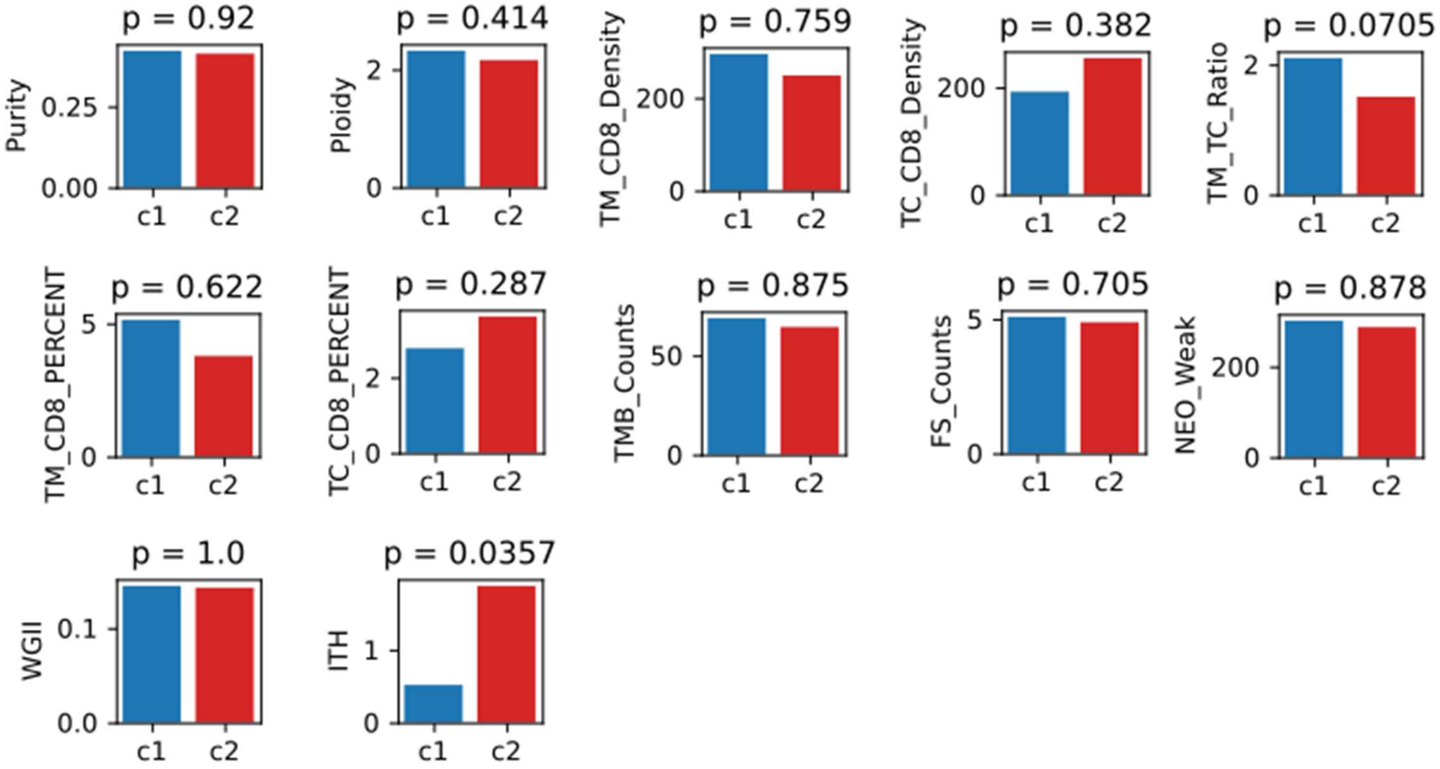

Figure S6. Comparison between two clusters generated from their positive correlation-based gene connectivity of pN, related to Figure 3.
Wilcoxon rank-sum test was conducted and p values below 0.05 were taken as significant. Cluster c1 has lower gene connectivity on average and significantly higher survival probability.

Clinical information of pN samples were retrieved from supplementary data of Braun 2020 paper.

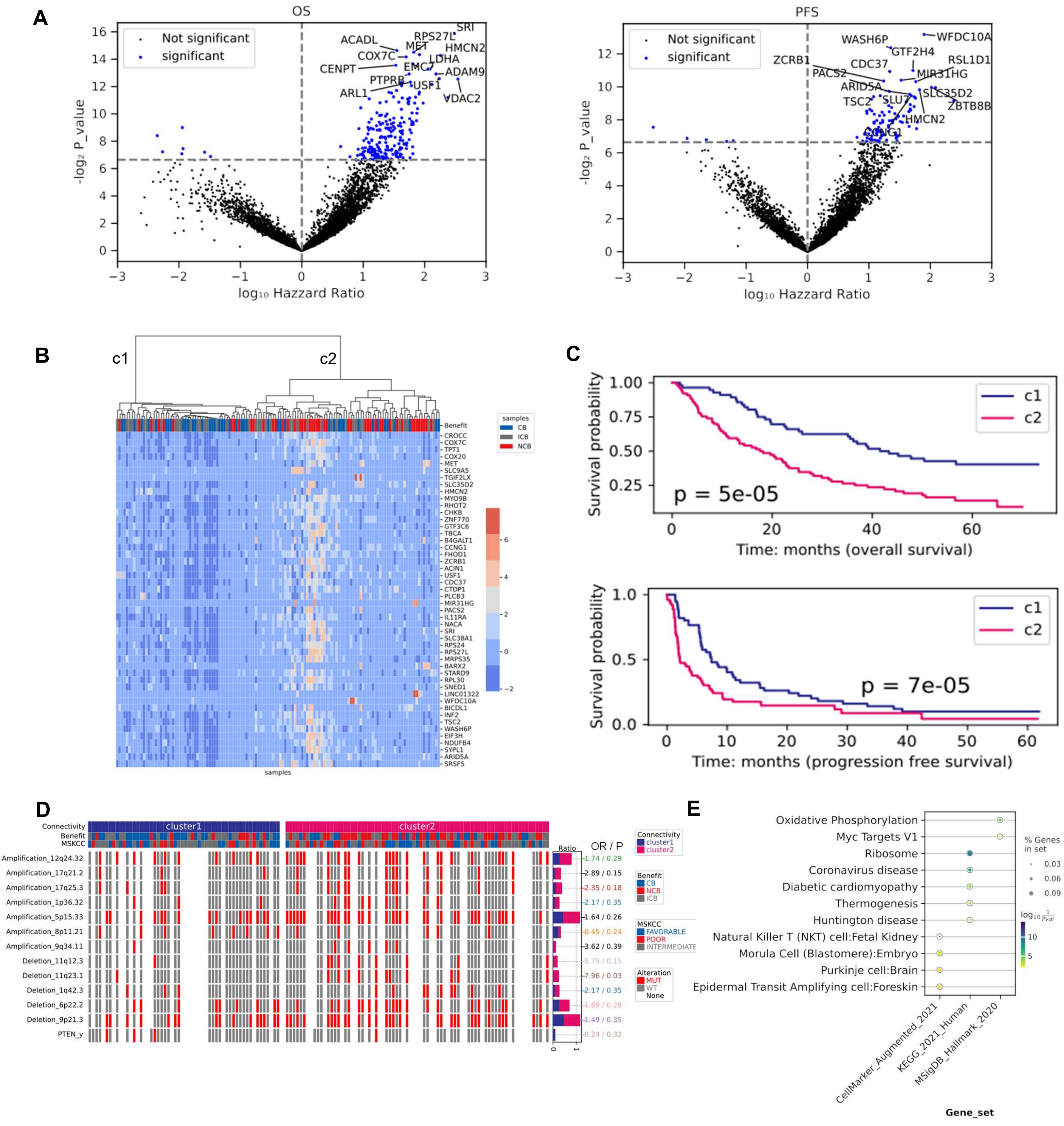

A
OS
Not significant
significant
SRI
RPS27L
ACADL
MET
HMCN2
COX7C
LDHA
CENPT
EMC7
ADAM9
PTPRB
USF1
ARL1
VDAC2
-log₂ P_value
log₁₀ Hazzard Ratio
PFS
WFDC10A
WASH6P
GTF2H4
RSL1D1
ZCRB1
CDC37
PACS2
MIR31HG
ARID5A
SLC35D2
TSC2
SLU7
ZBTB8B
HMCN2
CCNG1
B
c1
c2
Benefit
samples
CB
ICB
NCB
CROCC
COX7C
TPT1
COX20
MET
SLC9A5
TGIF2LX
SLC35D2
HMCN2
MYO9B
RHOT2
CHKB
ZNF770
GTF3C6
TBCA
B4GALT1
CCNG1
FHOD1
ZCRB1
ACIN1
USF1
CDC37
CTDP1
PLCB3
MIR31HG
PACS2
IL11RA
NACA
SRI
SLC38A1
RPS24
RPS27L
MRPS35
BARX2
STARD9
RPL30
SNED1
LINC01322
WFDC10A
BICDL1
INF2
TSC2
WASH6P
EIF3H
NDUFB4
SYPL1
ARID5A
SRSF5
C
Survival probability
c1
c2
p = 5e-05
Time: months (overall survival)
p = 7e-05
Time: months (progression free survival)
D
Connectivity
Benefit
MSKCC
cluster1
cluster2
Amplification_12q24.32
Amplification_17q21.2
Amplification_17q25.3
Amplification_1p36.32
Amplification_5p15.33
Amplification_8p11.21
Amplification_9q34.11
Deletion_11q12.3
Deletion_11q23.1
Deletion_1q42.3
Deletion_6p22.2
Deletion_9p21.3
PTEN_y
Ratio
OR / P
1.74 / 0.29
2.89 / 0.15
2.35 / 0.18
2.17 / 0.35
1.64 / 0.26
0.45 / 0.24
3.62 / 0.39
5.79 / 0.15
7.96 / 0.03
2.17 / 0.35
1.69 / 0.28
1.49 / 0.35
0.24 / 0.32
Connectivity
cluster1
cluster2
Benefit
CB
NCB
ICB
MSKCC
FAVORABLE
POOR
INTERMEDIATE
Alteration
MUT
WT
None
E
Oxidative Phosphorylation
Myc Targets V1
Ribosome
Coronavirus disease
Diabetic cardiomyopathy
Thermogenesis
Huntington disease
Natural Killer T (NKT) cell:Fetal Kidney
Morula Cell (Blastomere):Embryo
Purkinje cell:Brain
Epidermal Transit Amplifying cell:Foreskin
% Genes in set
CellMarker_Augmented_2021
KEGG_2021_Human
MSigDB_Hallmark_2020
Gene_set

F

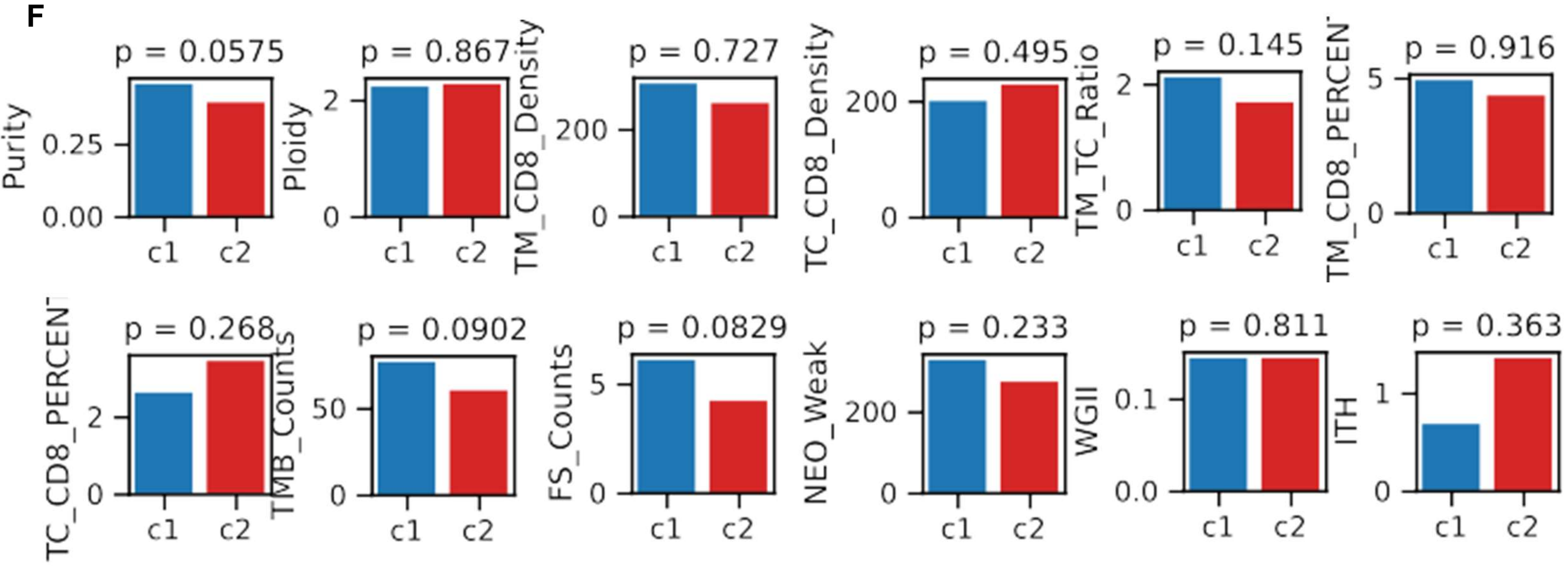

G

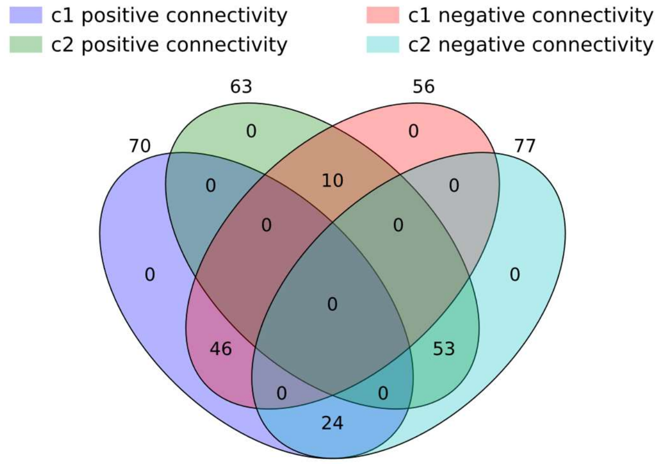

Figure S7. Comparison between two clusters generated from their negative correlation-based gene connectivity of pN, related to Figure 3.
(A). Volcano plot of significant genes related to overall survival and progression free survival. P value below 0.01 was taken as significant. (B). Hierarchy clustering of samples based on selected genes. Genes were used if they were significantly related to both OS and PFS. Two clusters were preferred (c1: 26 CB, 20 ICB, 10 NCB patients; c2: 18 CB, 23 ICB, 36 NCB patients; Fisher's exact test: p-value =0.0009). Out of 48 selected genes, 5 of them ('MIR31HG', 'BARX2', 'LINC01322', 'WFDC10A', 'MYO9B') in terms of their expression values were associated with survival data. (C). Survival analysis between cluster c1 (blue) and c2 (pink). P values were from the log rank test. (D). Distribution of chromosomal and gene mutation between clusters. Fisher test was conducted and p value less than 0.05 was considered as significant. (E). Overall representation analysis. Genes were selected as the union of significant genes related to OS and PFS. (F). Comparison of clinical features between two clusters. (G). Comparisons of patients between clusters from positive correlation-based gene connectivity and clusters from negative correlation-based gene connectivity.

Six genes ('ACIN1', 'INF2', 'MIR31HG', 'MYO9B', 'PLCB3', 'WFDC10A') were found in both positive and negative associations. MSKCC, referring to Memorial Sloan–Kettering Cancer Center prognostic model, is a clinical risk stratification score for metastatic renal cell carcinoma to classify patients into favorable, intermediate, or poor risk groups, supporting treatment decisions and survival prediction.

A

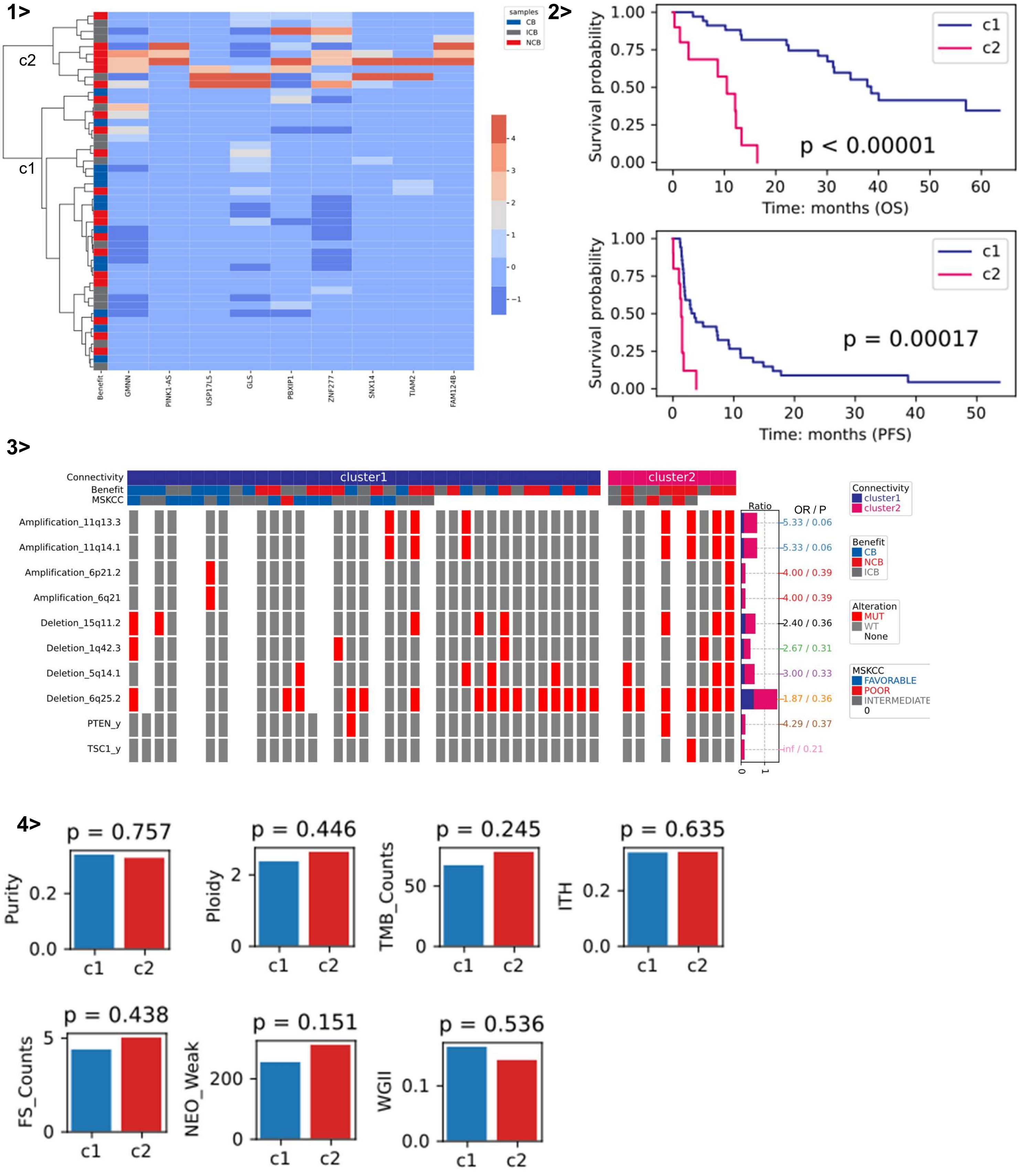

1>
c2
c1
samples
CB
ICB
NCB
Benefit
GMNN
PINK1-AS
USP17L5
GLS
PBXIP1
ZNF277
SNX14
TIAM2
FAM124B
2>
Survival probability
p < 0.00001
Time: months (OS)
c1
c2
p = 0.00017
Time: months (PFS)
3>
Connectivity
Benefit
MSKCC
cluster1
cluster2
Amplification_11q13.3
Amplification_11q14.1
Amplification_6p21.2
Amplification_6q21
Deletion_15q11.2
Deletion_1q42.3
Deletion_5q14.1
Deletion_6q25.2
PTEN_y
TSC1_y
Ratio
OR / P
5.33 / 0.06
5.33 / 0.06
4.00 / 0.39
4.00 / 0.39
2.40 / 0.36
2.67 / 0.31
3.00 / 0.33
1.87 / 0.36
4.29 / 0.37
inf / 0.21
Connectivity
cluster1
cluster2
Benefit
CB
NCB
ICB
Alteration
MUT
WT
None
MSKCC
FAVORABLE
POOR
INTERMEDIATE
0
4>
p = 0.757
Purity
p = 0.446
Ploidy
p = 0.245
TMB_Counts
p = 0.635
ITH
p = 0.438
FS_Counts
p = 0.151
NEO_Weak
p = 0.536
WGII

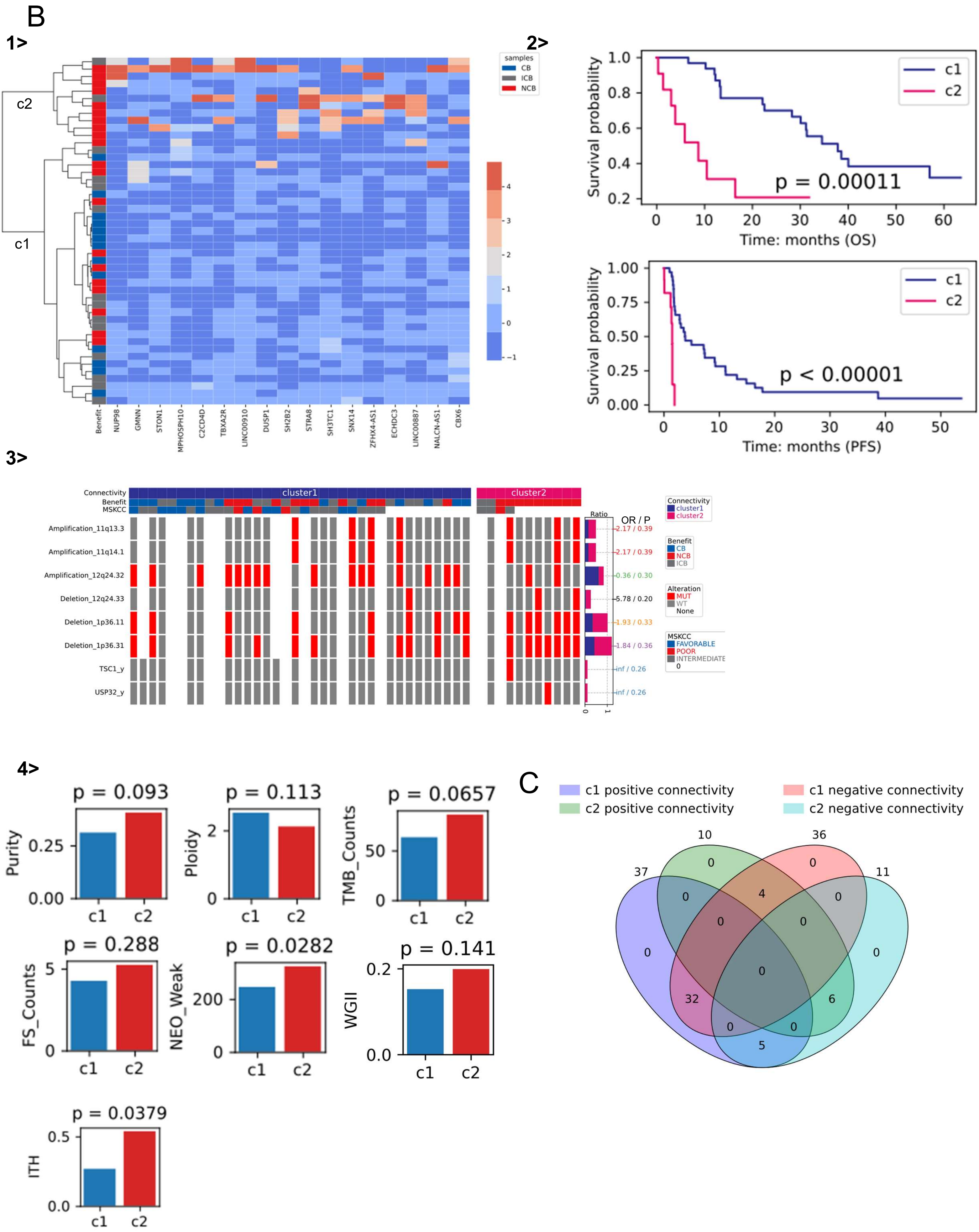
B
1>
c2
c1
samples
CB
ICB
NCB
Benefit
NUP98
GMNN
STON1
MPHOSPH10
C2CD4D
TBXA2R
LINC00910
DUSP1
SH2B2
STRA8
SH3TC1
SNX14
ZFHX4-AS1
ECHDC3
LINC00887
NALCN-AS1
CBX6
2>
Survival probability
Time: months (OS)
p = 0.00011
c1
c2
Survival probability
Time: months (PFS)
p < 0.00001
c1
c2
3>
Connectivity
Benefit
MSKCC
cluster1
cluster2
Amplification_11q13.3
Amplification_11q14.1
Amplification_12q24.32
Deletion_12q24.33
Deletion_1p36.11
Deletion_1p36.31
TSC1_y
USP32_y
Ratio
OR / P
2.17 / 0.39
2.17 / 0.39
0.36 / 0.30
5.78 / 0.20
1.93 / 0.33
1.84 / 0.36
inf / 0.26
inf / 0.26
Connectivity
cluster1
cluster2
Benefit
CB
NCB
ICB
Alteration
MUT
WT
None
MSKCC
FAVORABLE
POOR
INTERMEDIATE
0
4>
p = 0.093
Purity
p = 0.113
Ploidy
p = 0.0657
TMB_Counts
p = 0.288
FS_Counts
p = 0.0282
NEO_Weak
p = 0.141
WGII
p = 0.0379
ITH
c1
c2
C
c1 positive connectivity
c2 positive connectivity
c1 negative connectivity
c2 negative connectivity
37
10
36
11
4
32
6
5

Figure S8. Comparison between two clusters generated from their positive correlation (A) and negative correlation (B) based gene connectivity of mN. (C) Comparisons of patients between clusters from positive correlation-based gene connectivity and clusters from negative correlation-based gene connectivity, related to Figure 3. 1>. Hierarchy clustering of samples based on selected genes. Genes were selected if they were significantly related to both OS and PFS (9, and 17 genes were obtained from positive and negative based gene connectivity). Two clusters (c1 and c2) were preferred. Out of these genes, expression values of 1, and 6 of them ('USP17L5'; 'LINC00887', 'ZFHX4-AS1', 'TBXA2R', 'ECHDC3', 'NALCN-AS1', 'LINC00910') were associated with survival data. 2>. Survival analysis between cluster c1 (blue) and c2 (pink). P values were from the log rank test. 3>. Distribution of chromosomal and gene mutation between clusters. Fisher test was conducted and p value less than 0.05 was considered as significant. 4>. Comparison of clinical features between the two clusters.
Noted that we did not enrich any pathway for over representation analysis and two genes ('GMNN', 'SNX14') were found in both positive and negative associations.

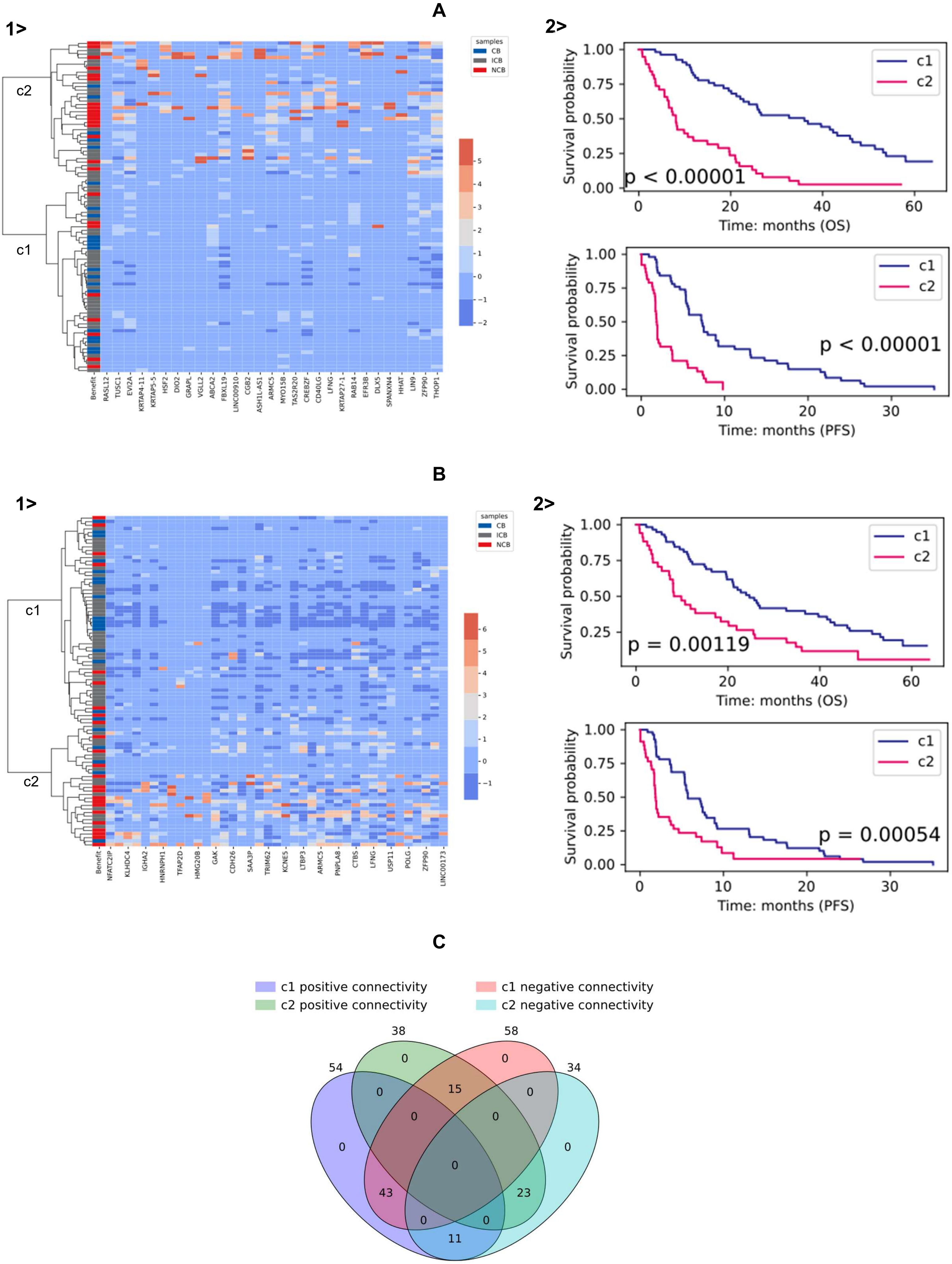

A
1>
c2
c1
samples
CB
ICB
NCB
Benefit
RASL12
TUSC1
EVI2A
KRTAP4-11
KRTAP5-5
HSF2
DIO2
GRAPL
VGLL2
ABCA2
FBXL19
LINC00910
CGB2
ASH1L-AS1
ARMC5
MYO15B
TAS2R20
CREBZF
CD40LG
LFNG
KRTAP27-1
RAB14
EFR3B
DLX5
SPANXN4
HHAT
LIN9
ZFP90
THOP1
2>
Survival probability
Time: months (OS)
p < 0.00001
Time: months (PFS)
p < 0.00001
c1
c2
B
1>
c1
c2
samples
CB
ICB
NCB
Benefit
NFATC2IP
KLHDC4
IGHA2
HNRNPH1
TFAP2D
HMG20B
GAK
CDH26
SAA3P
TRIM62
KCNE5
LTBP3
ARMC5
PNPLA8
CTBS
LFNG
USP11
POLG
ZFP90
LINC00173
2>
Survival probability
Time: months (OS)
p = 0.00119
Time: months (PFS)
p = 0.00054
c1
c2
C
c1 positive connectivity
c2 positive connectivity
c1 negative connectivity
c2 negative connectivity
54
38
58
34
15
43
23
11

Figure S9. Comparison between two clusters generated from their positive correlation (A) and negative correlation (B) based gene connectivity of pE. (C). Comparisons of patients between clusters from positive correlation-based gene connectivity and clusters from negative correlation-based gene connectivity, related to Figure 3. 1>. Hierarchy clustering of samples based on selected genes. Genes were selected if they were significantly related to both OS and PFS (29, and 39 genes were obtained from positive and negative based gene connectivity). Two clusters (c1 and c2) were preferred. 2>. Survival analysis between cluster c1 (blue) and c2 (pink). P values were from the log rank test.

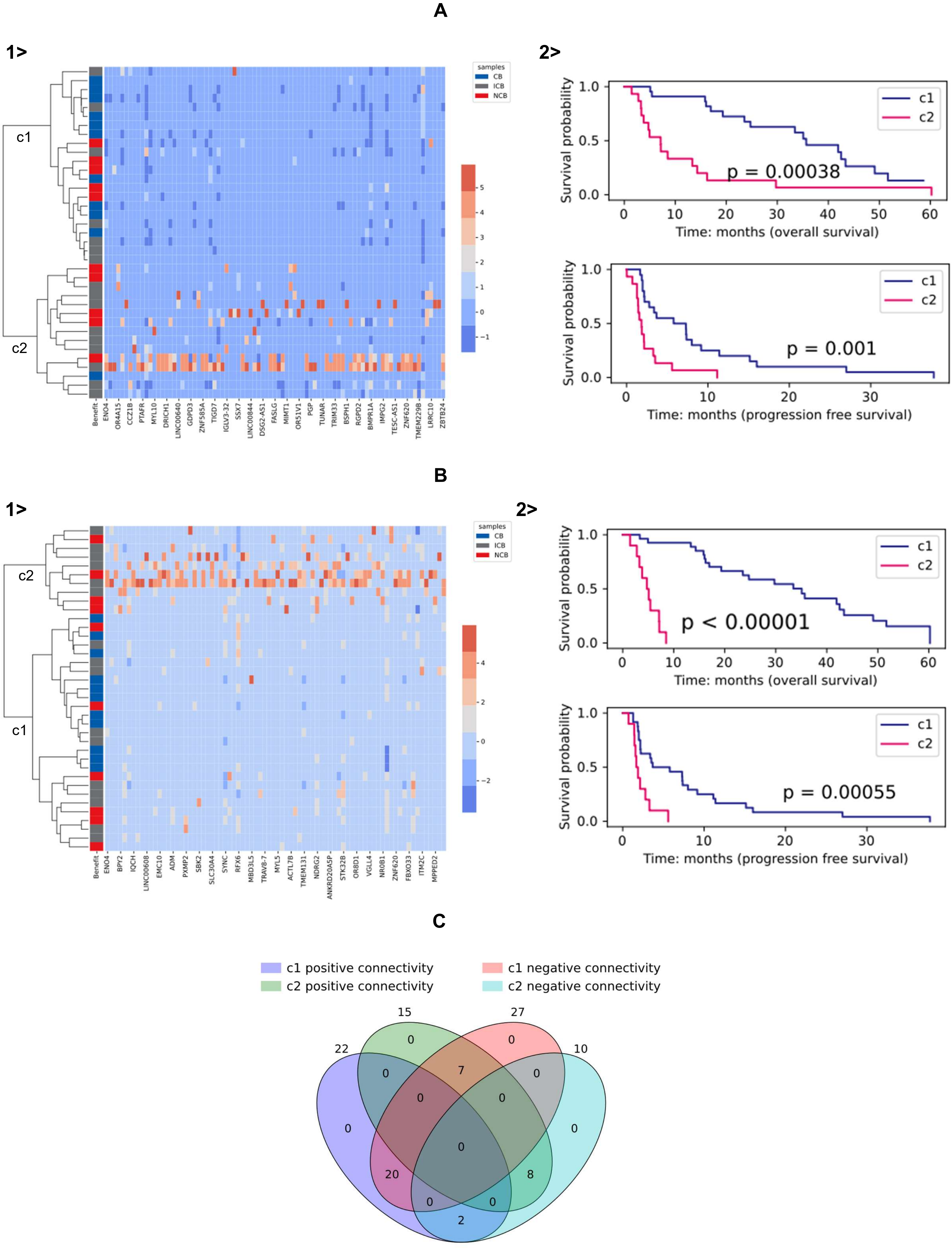
A
1>
2>
c1
c2
samples
CB
ICB
NCB
Survival probability
Time: months (overall survival)
p = 0.00038
Time: months (progression free survival)
p = 0.001
B
1>
2>
p < 0.00001
p = 0.00055
C
c1 positive connectivity
c2 positive connectivity
c1 negative connectivity
c2 negative connectivity
22
15
27
10

Figure S10. Comparison between two clusters generated from their positive correlation (A) and negative correlation (B) based gene connectivity of mE. (C). Comparison of patients between clusters from positive correlation-based gene connectivity and clusters from negative correlation-based gene connectivity, related to Figure 3. 1>. Hierarchy clustering of samples based on selected genes. Genes were selected if they were significantly related to either OS or PFS (78, and 85 genes were obtained from positive and negative based gene connectivity). No common gene was found between OS significant genes and PFS significant genes. Two clusters (c1 and c2) were preferred. 2>. Survival analysis between cluster c1 (blue) and c2 (pink). P values were from log rank tests.

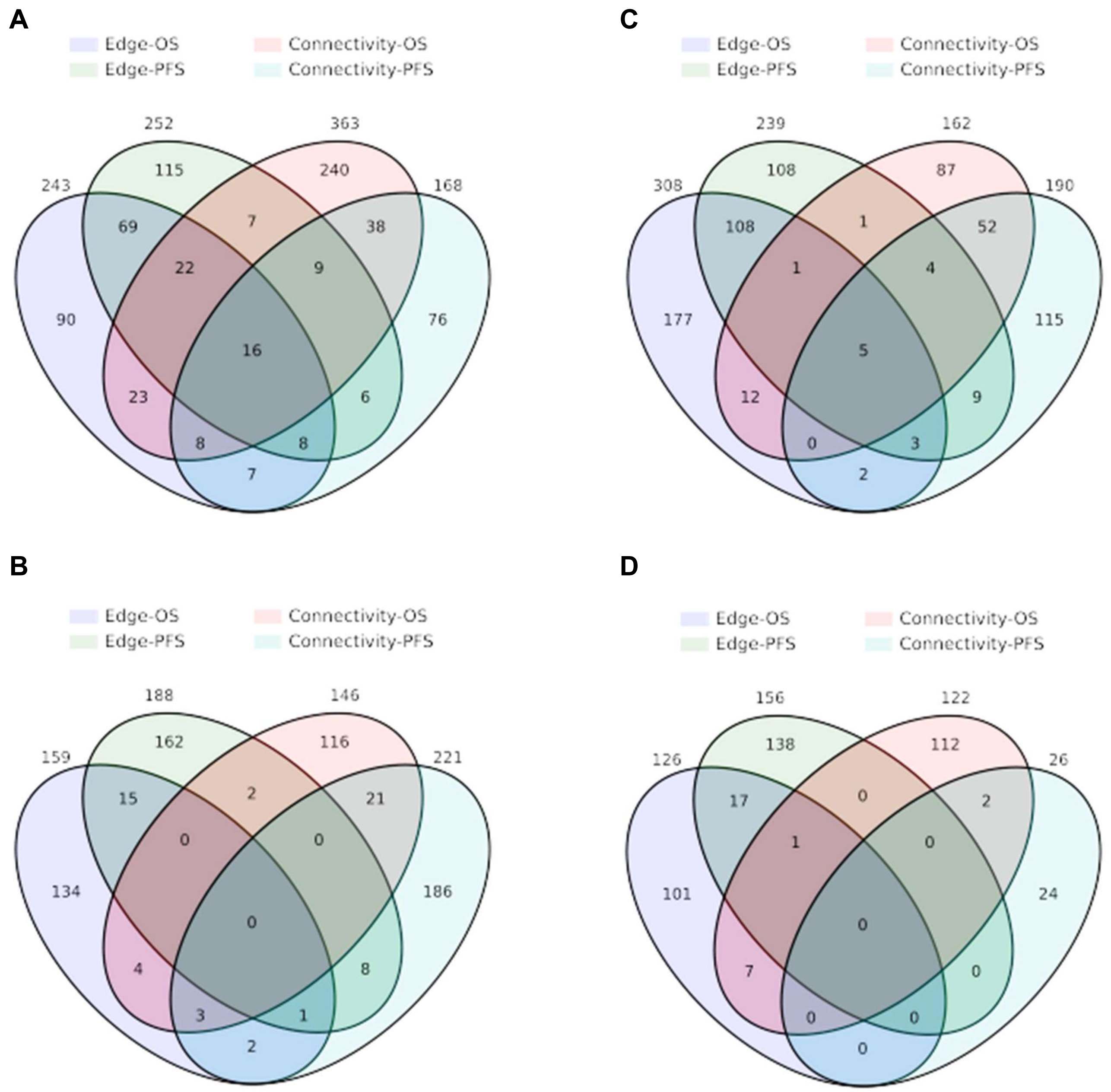

Figure S11. Venn plots for genes from gene connectivity and edges, related to Figure 3, 4 . The number of overlapping genes between edges and connectivity were indicated above. (A). pN. (B). mN. (C). pE. (D). mE.

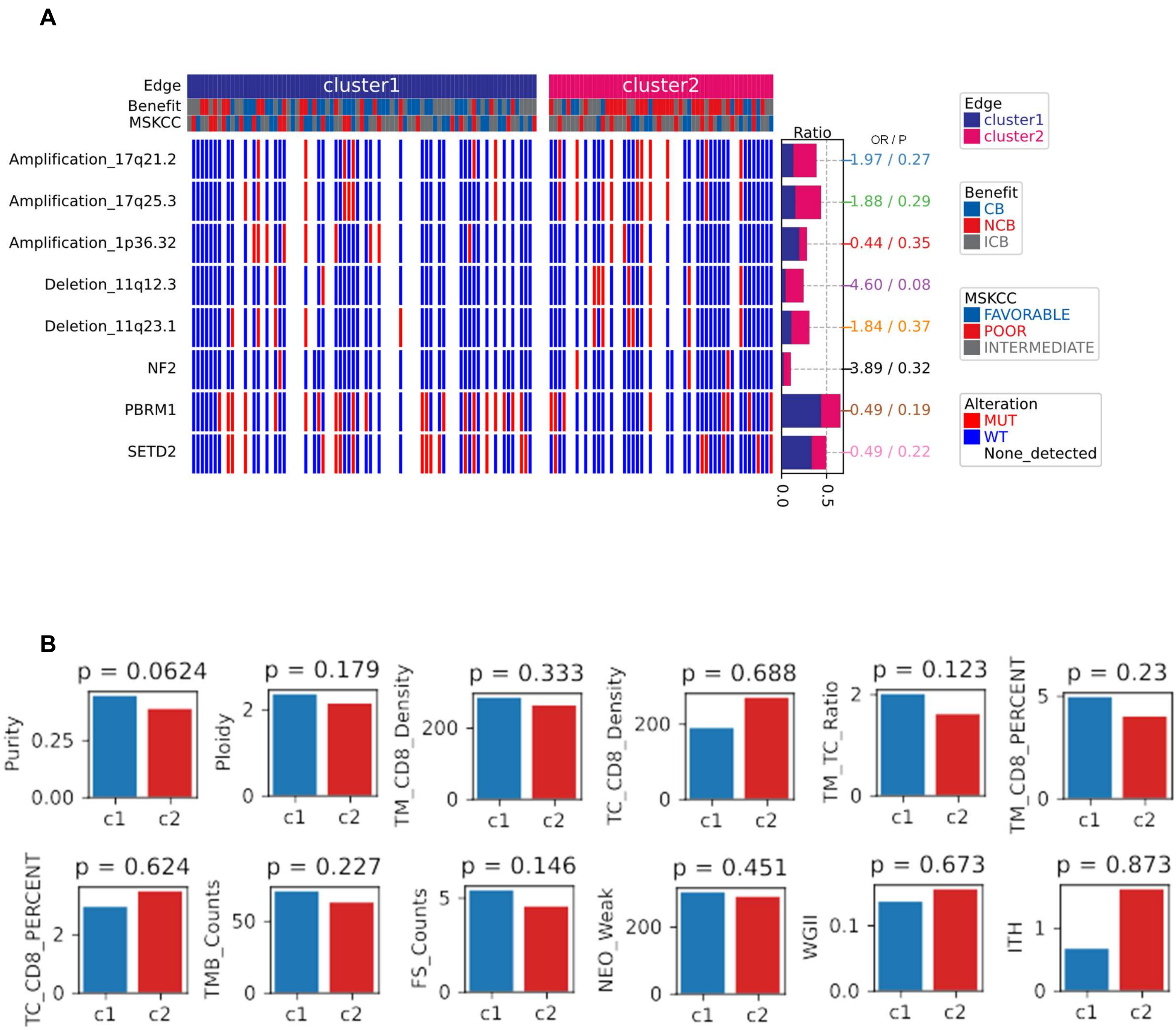

Figure S12. Comparison of genomic mutation and clinical features between two clusters generated from selected edges of pN ssGCNs, related to Figure 4. A. Mutation plot. B. Bar plots for clinical features. Wilcoxon rank sum tests were conducted and p values below 0.05 were taken as significant. Gene pairs in cluster c1 have higher Pearson correlation on average and cluster c1 were associated with higher survival probability.

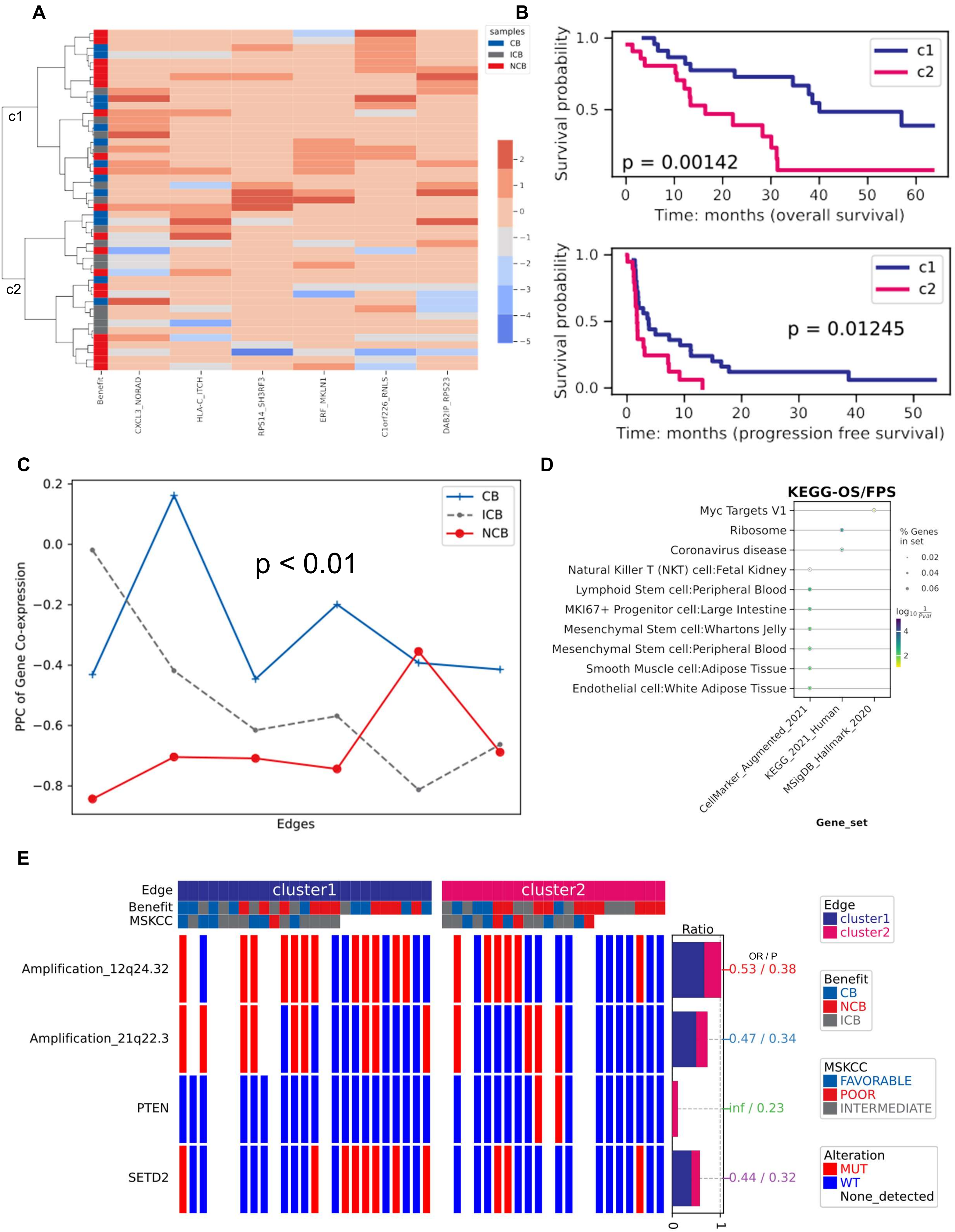

A
B
C
D
E
c1
c2
samples
CB
ICB
NCB
Benefit
CXCL3_NORAD
HLA-C_ITCH
RPS14_SH3RF3
ERF_MKLN1
C1orf226_RNLS
DAB2IP_RPS23
Survival probability
p = 0.00142
Time: months (overall survival)
p = 0.01245
Time: months (progression free survival)
PPC of Gene Co-expression
p < 0.01
Edges
KEGG-OS/FPS
Myc Targets V1
Ribosome
Coronavirus disease
Natural Killer T (NKT) cell:Fetal Kidney
Lymphoid Stem cell:Peripheral Blood
MKI67+ Progenitor cell:Large Intestine
Mesenchymal Stem cell:Whartons Jelly
Mesenchymal Stem cell:Peripheral Blood
Smooth Muscle cell:Adipose Tissue
Endothelial cell:White Adipose Tissue
% Genes in set
CellMarker_Augmented_2021
KEGG_2021_Human
MSigDB_Hallmark_2020
Gene_set
Edge
cluster1
cluster2
Benefit
MSKCC
Amplification_12q24.32
Amplification_21q22.3
PTEN
SETD2
Ratio
OR / P
0.53 / 0.38
0.47 / 0.34
inf / 0.23
0.44 / 0.32
FAVORABLE
POOR
INTERMEDIATE
Alteration
MUT
WT
None_detected

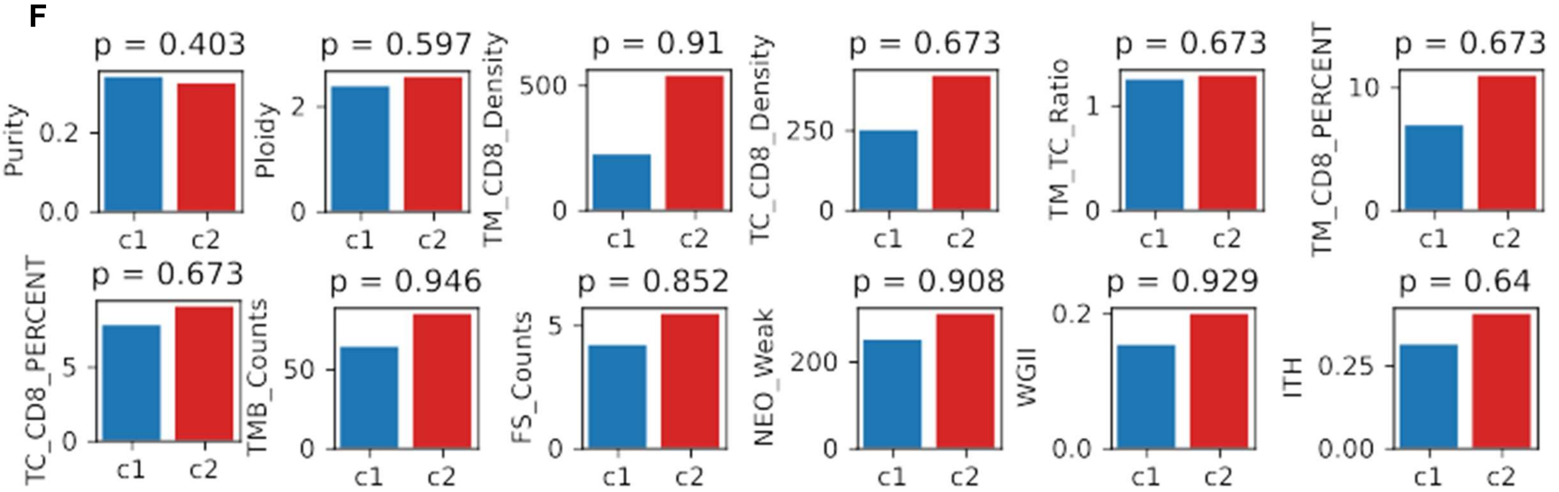

Figure S13. Edges classified samples of mN, related to Figure 4. (A). Hierarchy clustering of samples using 6 edges significantly associated with both OS and PFS. (B). Survival analysis between two clusters c1 (blue) and c2 (pink). P values were from the log rank test. (C). Distributions of pearson correlation coefficients (PCC) for mN samples of CB (blue), ICB (grey) and NCB (red). Wilcoxon rank sum tests were conducted between CB and NCB patients. (D). Overall representation analysis. Genes were selected as the union of genes from significant edges significantly related to OS and PFS. (E). Mutation plot. Fisher test was conducted. (F). Bar plots for clinical features. Wilcoxon rank sum tests were conducted. p values below 0.05 were taken as significant.

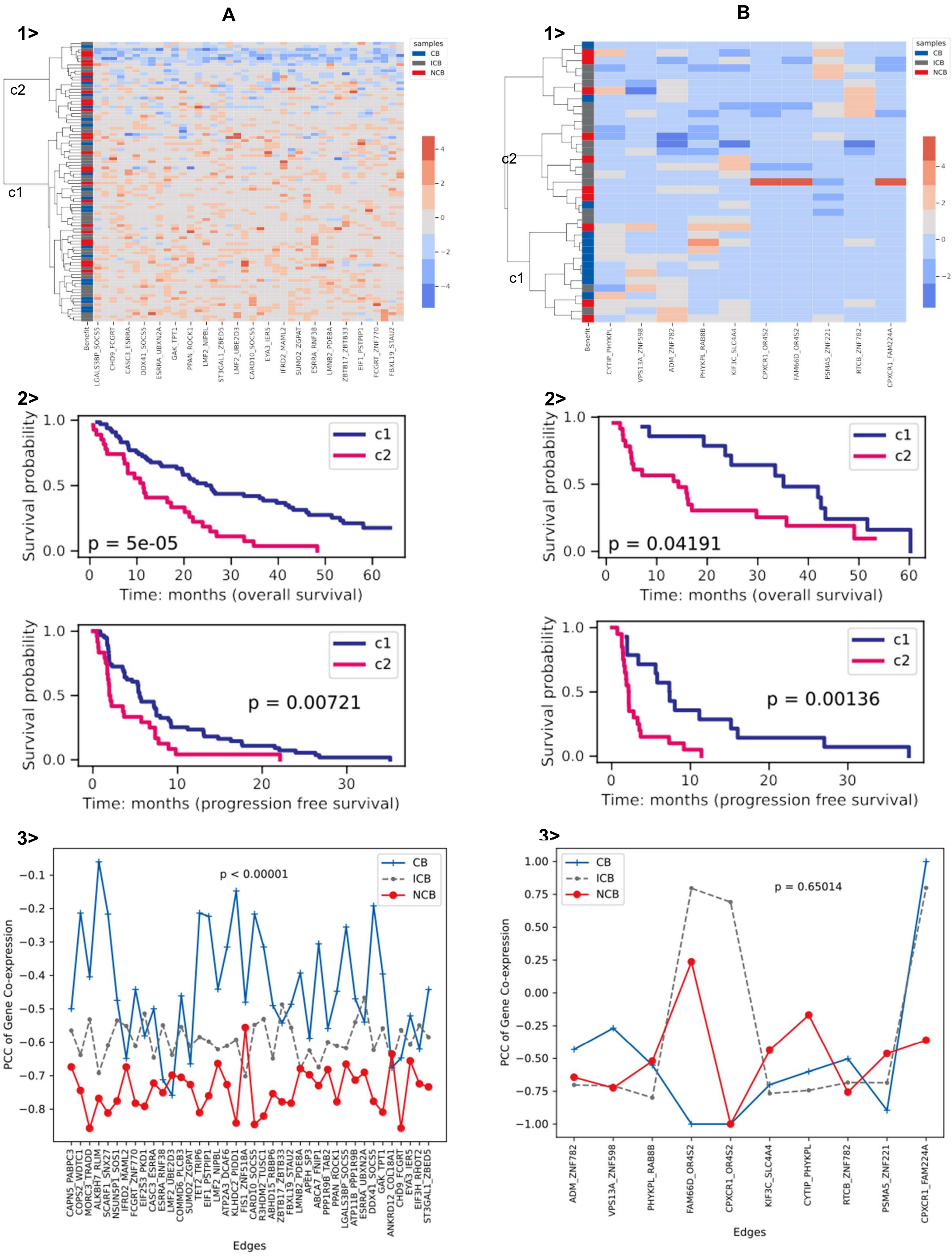

A
B
1>
samples
CB
ICB
NCB
c2
c1
Benefit
LGALS3BP_SOCS5
CHD9_FCGRT
CASC3_ESRRA
DDX41_SOCS5
ESRRA_UBXN2A
GAK_TPT1
PPAN_ROCK1
LMF2_NIPBL
ST3GAL1_ZBED5
LMF2_UBE2D3
CARD10_SOCS5
EYA3_IER5
IFRD2_MAML2
SUMO2_ZGPAT
ESRRA_RNF38
LMNB2_PDE8A
ZBTB17_ZBTB33
EIF1_PSTPIP1
FCGRT_ZNF770
FBXL19_STAU2
CYTIP_PHYKPL
VPS13A_ZNF598
ADM_ZNF782
PHYKPL_RAB8B
KIF3C_SLC4A4
CPXCR1_OR4S2
FAM66D_OR4S2
PSMA5_ZNF221
RTCB_ZNF782
CPXCR1_FAM224A
2>
Survival probability
Time: months (overall survival)
Time: months (progression free survival)
p = 5e-05
p = 0.00721
p = 0.04191
p = 0.00136
3>
PCC of Gene Co-expression
Edges
p < 0.00001
p = 0.65014
CAPN5_PABPC3
COPS2_WDTC1
MORC3_TRADD
ALKBH7_RLIM
SCARF1_SNX27
NSUN5P1_SOS1
IFRD2_MAML2
FCGRT_ZNF770
EIF2S3_PKD1
CASC3_ESRRA
ESRRA_RNF38
LMF2_UBE2D3
COMMD6_PLCB3
SUMO2_ZGPAT
TET2_PSTPIP1
EIF1_PSTPIP1
LMF2_NIPBL
ATP2A3_DCAF6
KLHDC2_PIDD1
FIS1_ZNF518A
CARD10_SOCS5
R3HDM2_TUSC1
ABHD15_RBBP6
ZBTB17_ZBTB33
FBXL19_STAU2
LMNB2_PDE8A
APEH_SP3
ABCA7_FNIP1
PPP1R9B_TAB2
PPAN_ROCK1
LGALS3BP_SOCS5
ATP11B_PPP1R9B
ESRRA_UBXN2A
DDX41_SOCS5
GAK_TPT1
ANKRD12_COL18A1
CHD9_FCGRT
EYA3_IER5
EIF3H_RHOT2
ST3GAL1_ZBED5

Figure S14. Edges classified samples of pE (A) and mE (B), related to Figure 4. 1>. Hierarchy clustering of samples using 40/ 10 edges significantly associated with both OS and PFS. 2>. Survival analysis between two clusters c1 (blue) and c2 (pink). P values were from the log rank test. 3>. Distributions of Pearson correlation coefficients (PCC) for mN samples of CB (blue), ICB (grey) and NCB (red). Wilcoxon rank-sum tests were conducted between CB and NCB patients.

A

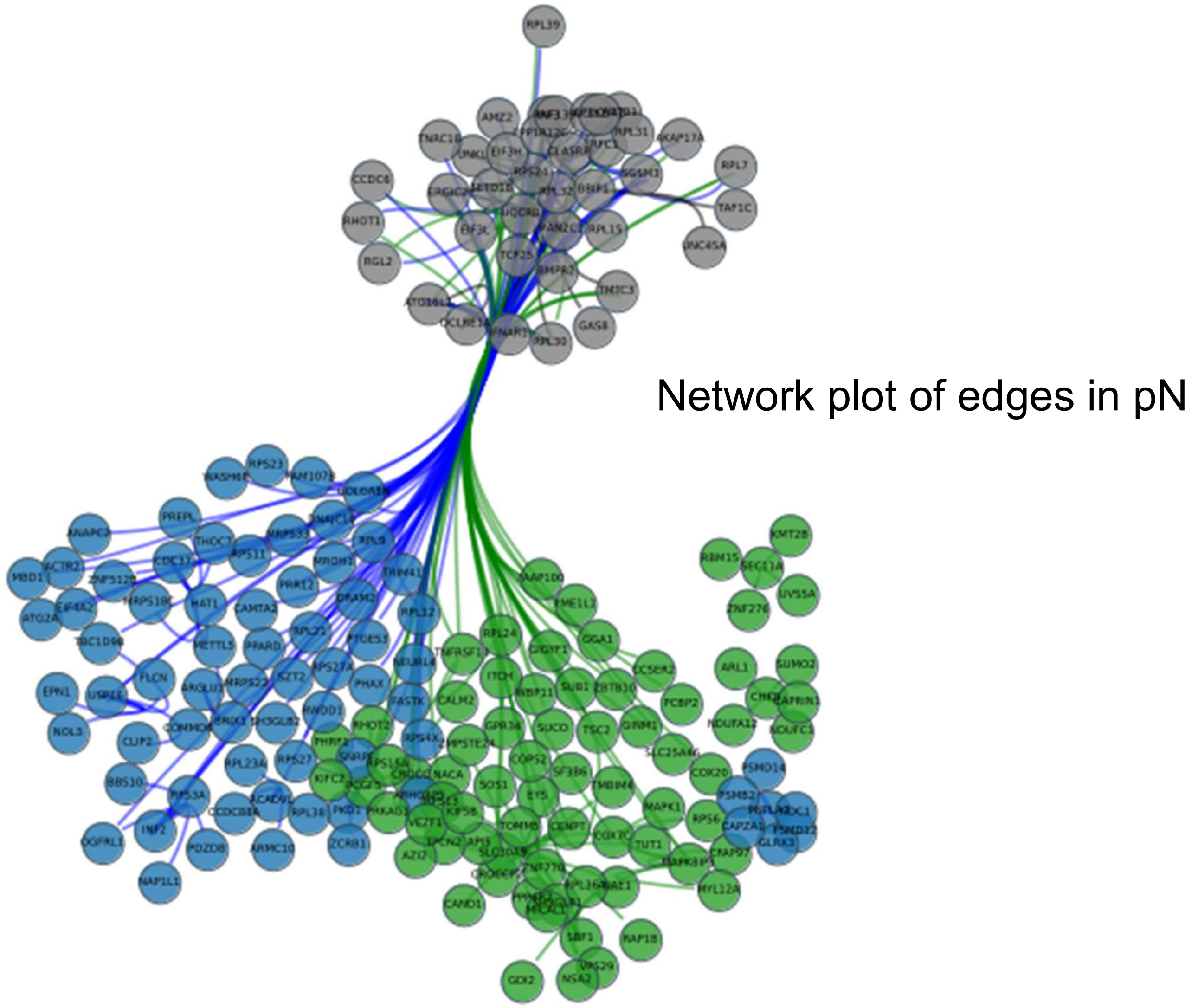

B

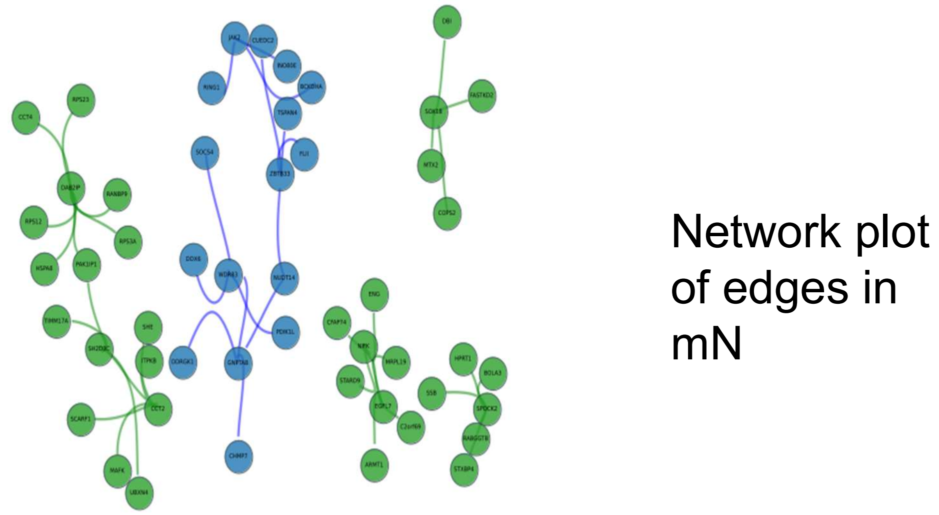

C

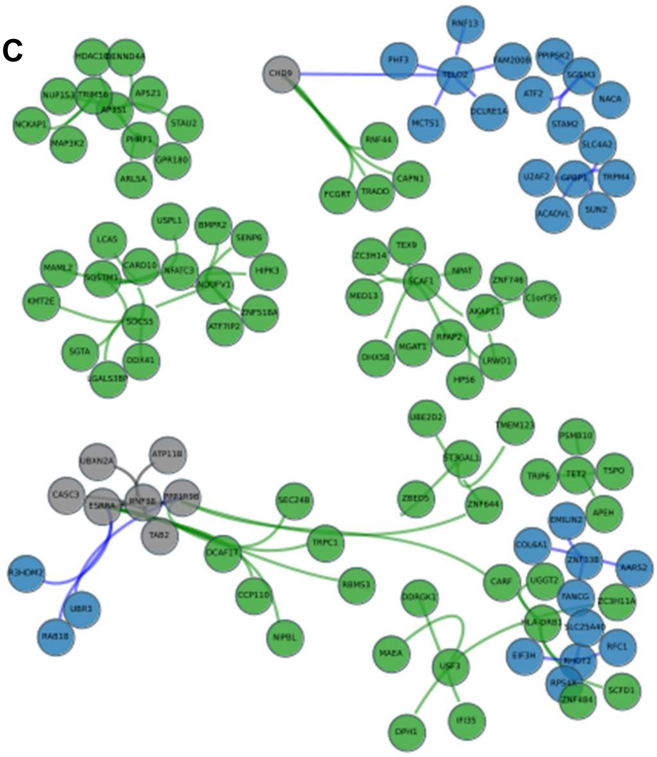

Network plot of edges in pE

D

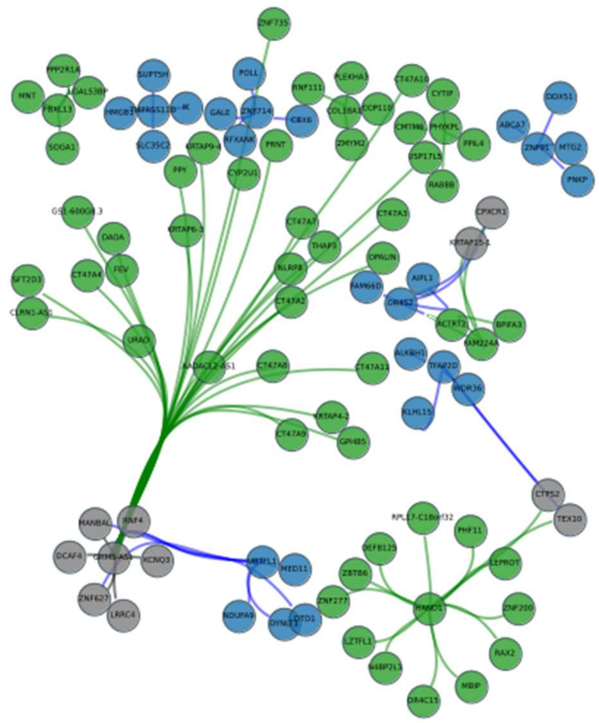

Network plot of edges in mE

Figure S15. Network plots of selected edges significantly associated with either OS or PFS, related to Figure 4. Genes, showing more than 3 times across edges, were displayed here. Black color indicated genes from both OS and PFS, and green / blue color refers that genes related with only OS or PFS. (A). pN. (B). mN. (C). pE. (D). mE. Noted that for mN and Me, significant edges were selected by p value 0f 0.05.

OS significant pathways (left) and PFS significant pathways (right)

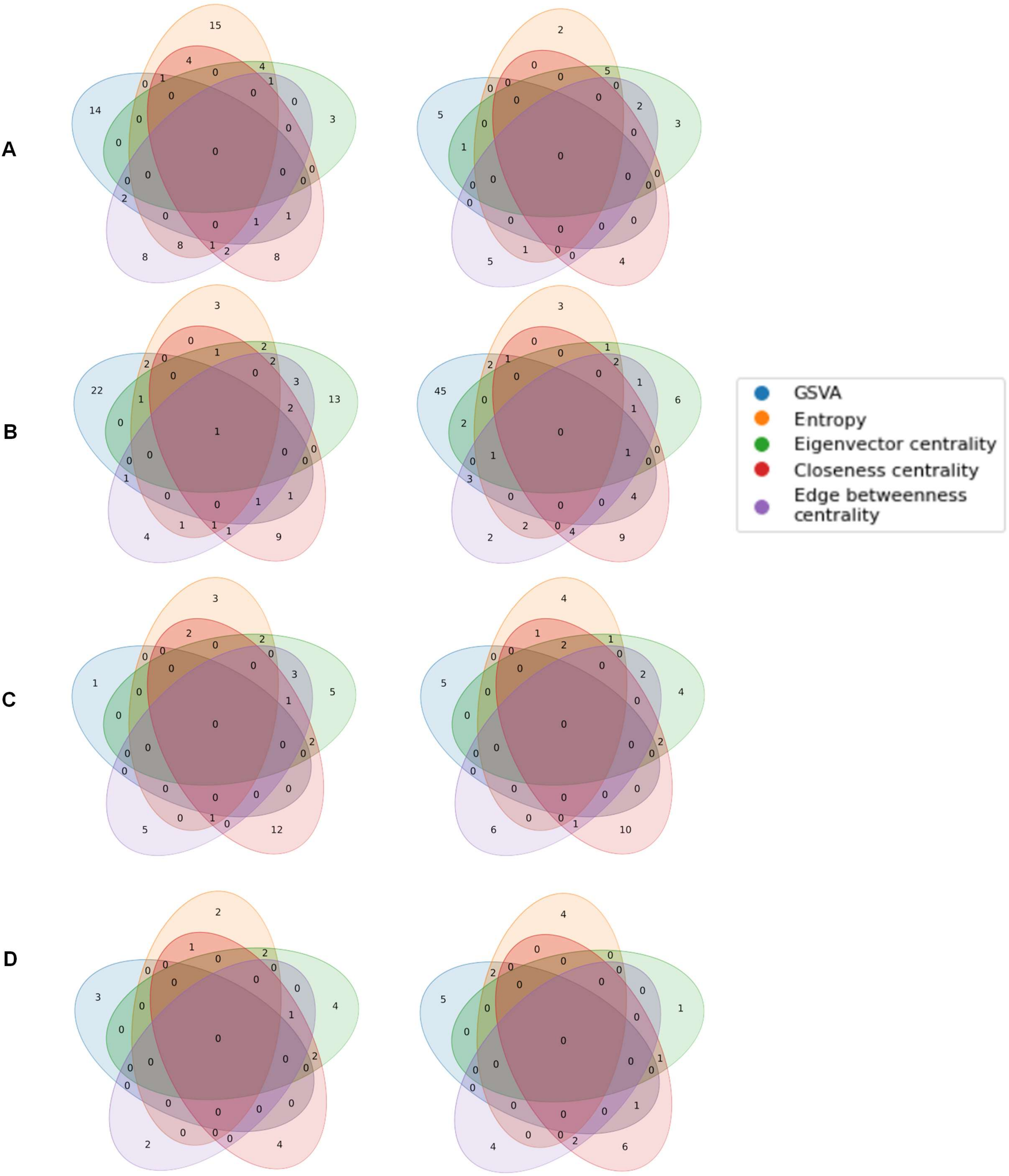

Figure S16. Venn plot of signficant pathways in the subcohorts: pN (A), mN (B), pE (C), mE (D), related to Figure 5.

The significant pathways were selected if their p values were less than 0.05 from the cox regression models. These significant pathways were barely overlapped with the others and it may be explained that they were calculated based on gene expression, network complexity and the influence of genes or edges.

A

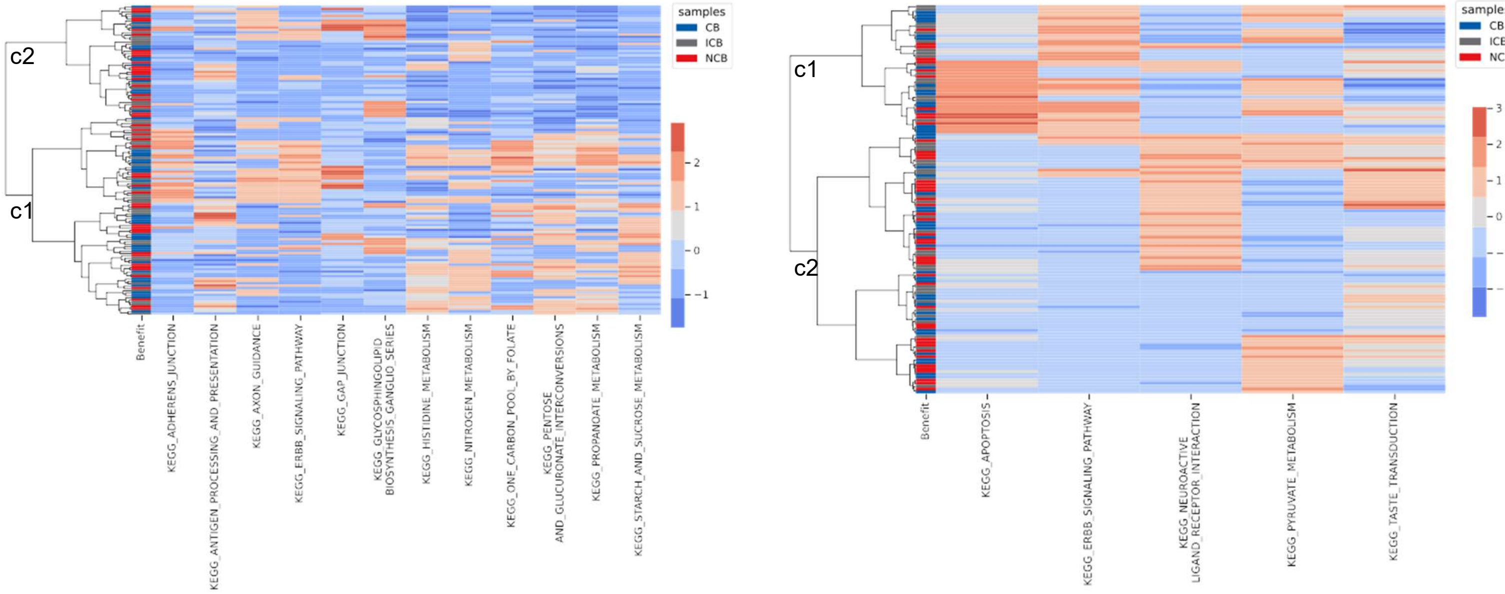

c2
c1
samples
CB
ICB
NCB
Benefit
KEGG_ADHERENS_JUNCTION
KEGG_ANTIGEN_PROCESSING_AND_PRESENTATION
KEGG_AXON_GUIDANCE
KEGG_ERBB_SIGNALING_PATHWAY
KEGG_GAP_JUNCTION
KEGG_GLYCOSPHINGOLIPID BIOSYNTHESIS_GANGLIO_SERIES
KEGG_HISTIDINE_METABOLISM
KEGG_NITROGEN_METABOLISM
KEGG_ONE_CARBON_POOL_BY_FOLATE
KEGG_PENTOSE AND_GLUCURONATE_INTERCONVERSIONS
KEGG_PROPANOATE_METABOLISM
KEGG_STARCH_AND_SUCROSE_METABOLISM
KEGG_APOPTOSIS
KEGG_NEUROACTIVE LIGAND_RECEPTOR_INTERACTION
KEGG_PYRUVATE_METABOLISM
KEGG_TASTE_TRANSDUCTION

B

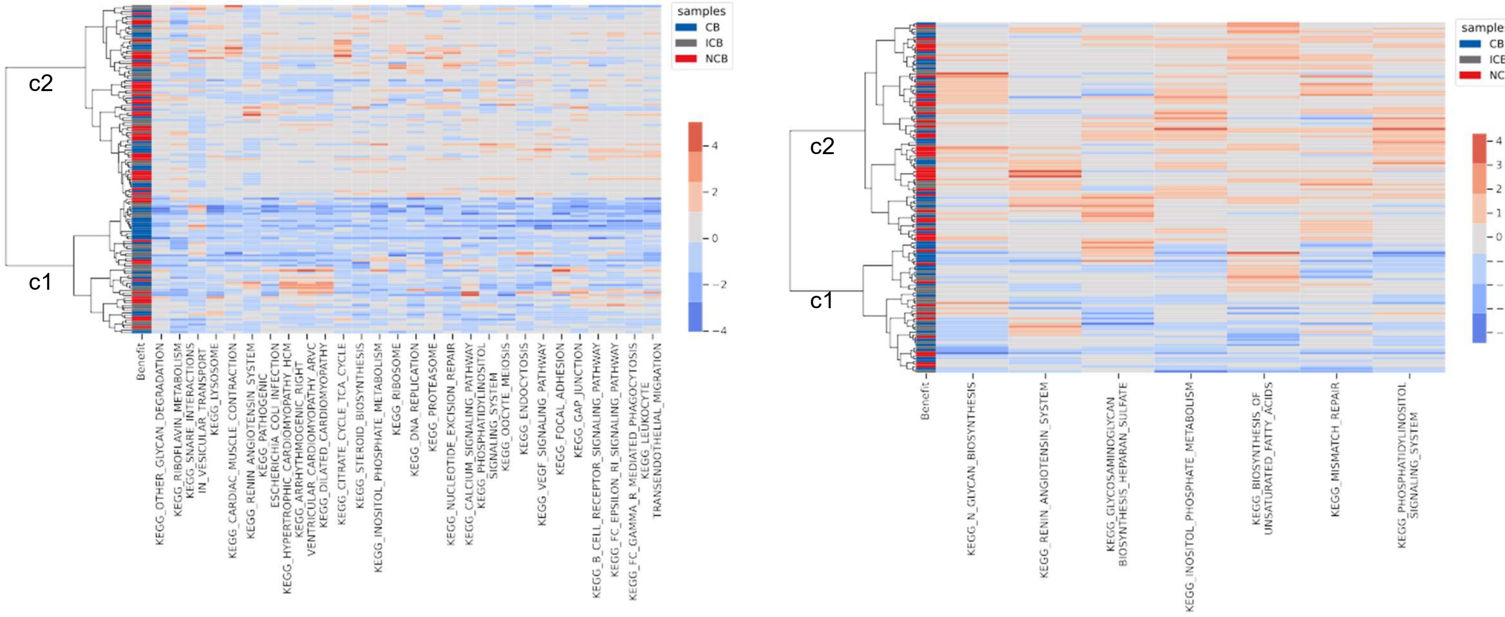

c2
c1
samples
CB
ICB
NCB
Benefit
KEGG_OTHER_GLYCAN_DEGRADATION
KEGG_RIBOFLAVIN_METABOLISM
KEGG_SNARE_INTERACTIONS IN_VESICULAR_TRANSPORT
KEGG_LYSOSOME
KEGG_CARDIAC_MUSCLE_CONTRACTION
KEGG_RENIN_ANGIOTENSIN_SYSTEM
KEGG_PATHOGENIC ESCHERICHIA_COLI_INFECTION
KEGG_HYPERTROPHIC_CARDIOMYOPATHY_HCM
KEGG_ARRHYTHMOGENIC_RIGHT VENTRICULAR_CARDIOMYOPATHY_ARVC
KEGG_DILATED_CARDIOMYOPATHY
KEGG_CITRATE_CYCLE_TCA_CYCLE
KEGG_STEROID_BIOSYNTHESIS
KEGG_INOSITOL_PHOSPHATE_METABOLISM
KEGG_RIBOSOME
KEGG_DNA_REPLICATION
KEGG_PROTEASOME
KEGG_NUCLEOTIDE_EXCISION_REPAIR
KEGG_CALCIUM_SIGNALING_PATHWAY
KEGG_PHOSPHATIDYLINOSITOL SIGNALING_SYSTEM
KEGG_OOCYTE_MEIOSIS
KEGG_ENDOCYTOSIS
KEGG_VEGF_SIGNALING_PATHWAY
KEGG_FOCAL_ADHESION
KEGG_GAP_JUNCTION
KEGG_B_CELL_RECEPTOR_SIGNALING_PATHWAY
KEGG_FC_EPSILON_RI_SIGNALING_PATHWAY
KEGG_FC_GAMMA_R_MEDIATED_PHAGOCYTOSIS
KEGG_LEUKOCYTE TRANSENDOTHELIAL_MIGRATION
KEGG_N_GLYCAN_BIOSYNTHESIS
KEGG_GLYCOSAMINOGLYCAN BIOSYNTHESIS_HEPARAN_SULFATE
KEGG_BIOSYNTHESIS_OF UNSATURATED_FATTY_ACIDS
KEGG_MISMATCH_REPAIR

C

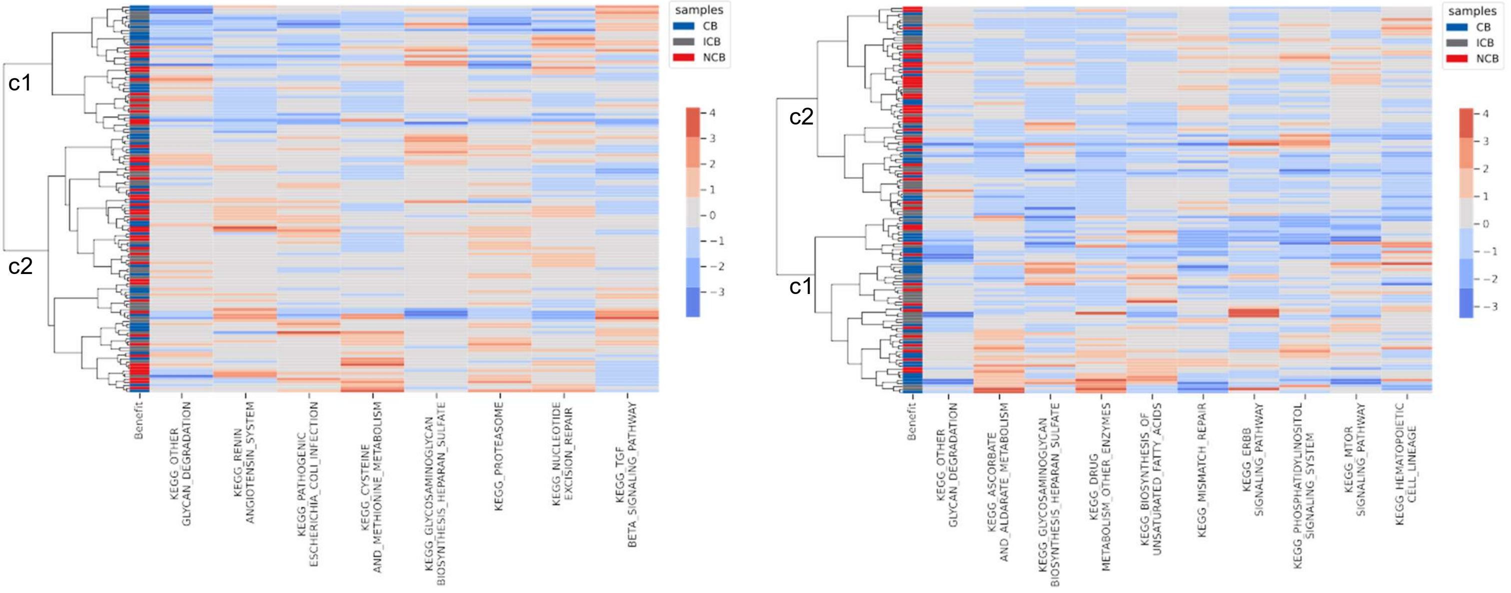

c1
c2
samples
CB
ICB
NCB
Benefit
KEGG_OTHER GLYCAN_DEGRADATION
KEGG_RENIN ANGIOTENSIN_SYSTEM
KEGG_PATHOGENIC ESCHERICHIA_COLI_INFECTION
KEGG_CYSTEINE AND_METHIONINE_METABOLISM
KEGG_GLYCOSAMINOGLYCAN BIOSYNTHESIS_HEPARAN_SULFATE
KEGG_PROTEASOME
KEGG_NUCLEOTIDE EXCISION_REPAIR
KEGG_TGF BETA_SIGNALING_PATHWAY
KEGG_ASCORBATE AND_ALDARATE_METABOLISM
KEGG_DRUG METABOLISM_OTHER_ENZYMES
KEGG_BIOSYNTHESIS_OF UNSATURATED_FATTY_ACIDS
KEGG_MISMATCH_REPAIR
KEGG_ERBB SIGNALING_PATHWAY
KEGG_PHOSPHATIDYLINOSITOL SIGNALING_SYSTEM
KEGG_MTOR SIGNALING_PATHWAY
KEGG_HEMATOPOIETIC CELL_LINEAGE

D

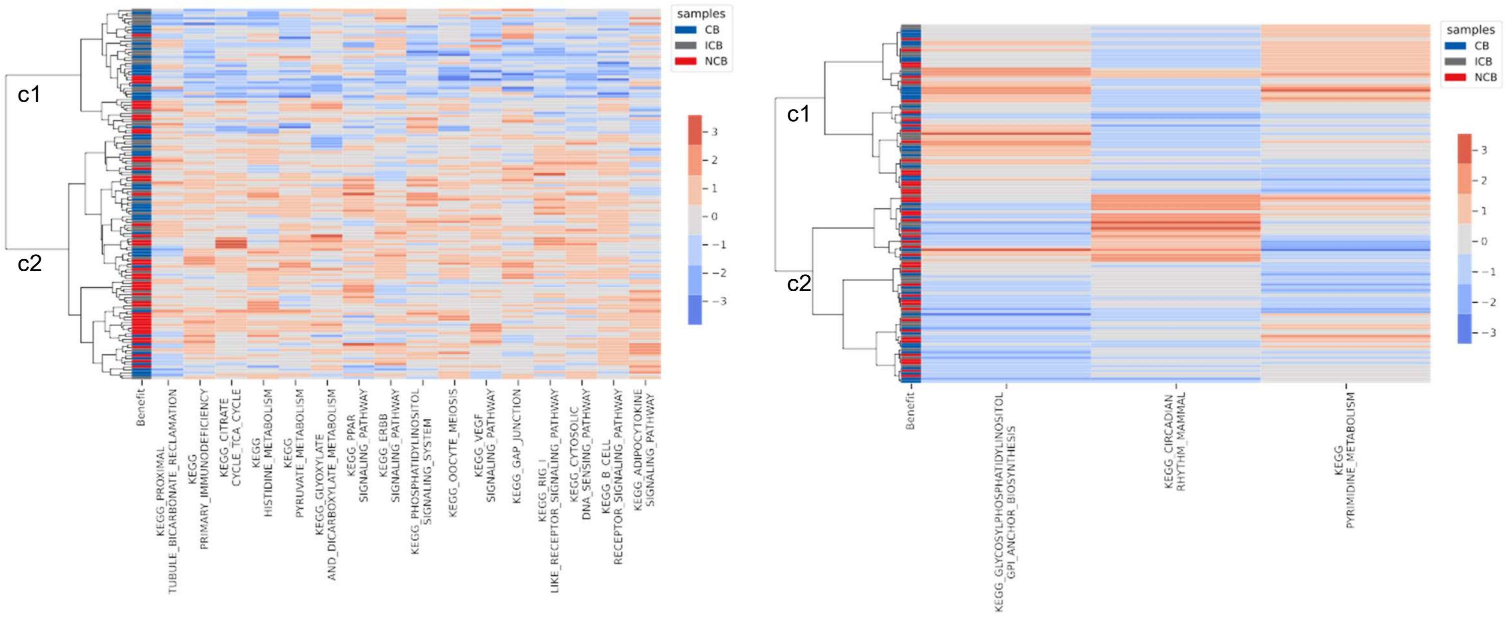

E

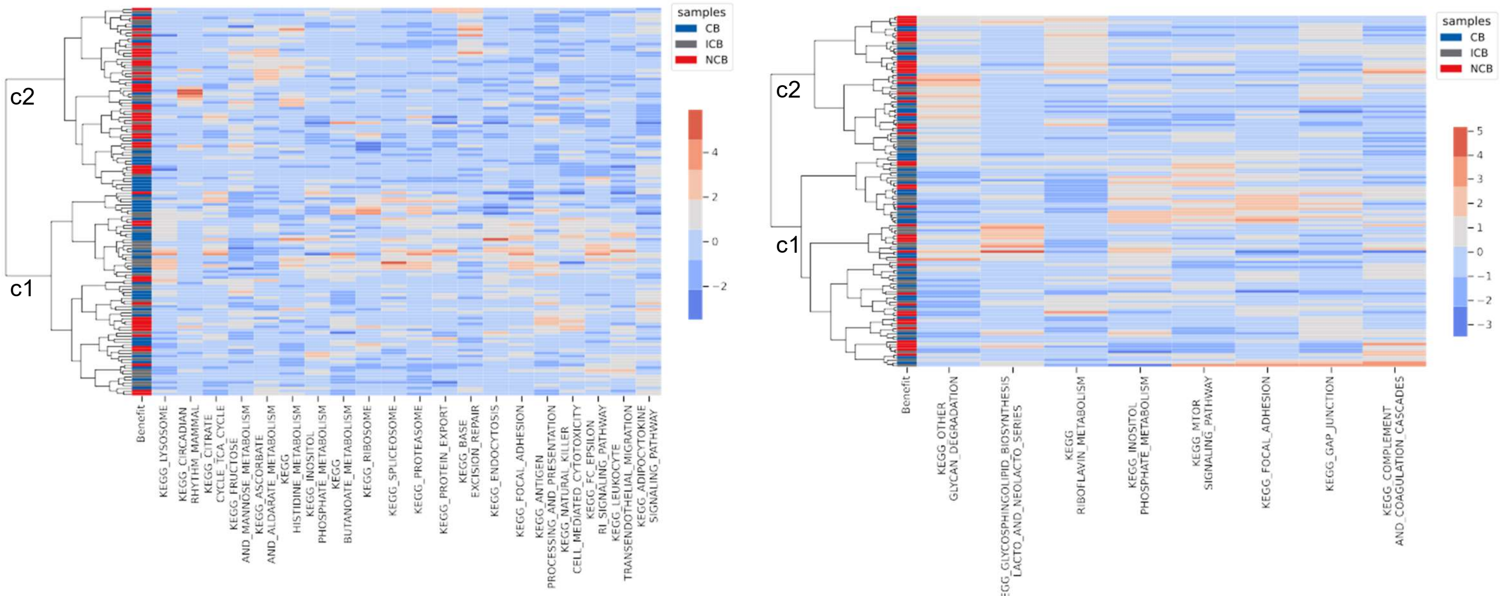

Figure S17. Clustermap of pN samples using pathway scores, related to Figure 5. (A). 12 (OS)/ 5 (PFS) significant pathways based on GSVA. (B). 28 (OS)/ 7 (PFS) significant pathways based on entropy. (C). 8 (OS)/ 10 (PFS) significant pathways based on gene eigenvector centrality scores. (D). 16 (OS)/ 3 (PFS) significant pathways based on gene closeness centrality scores. (E).20 (OS)/ 8 (PFS) significant pathways based on edge betweenness centrality score. Note that irrelevant pathways were filtered out for the clustering step in pathway analysis.

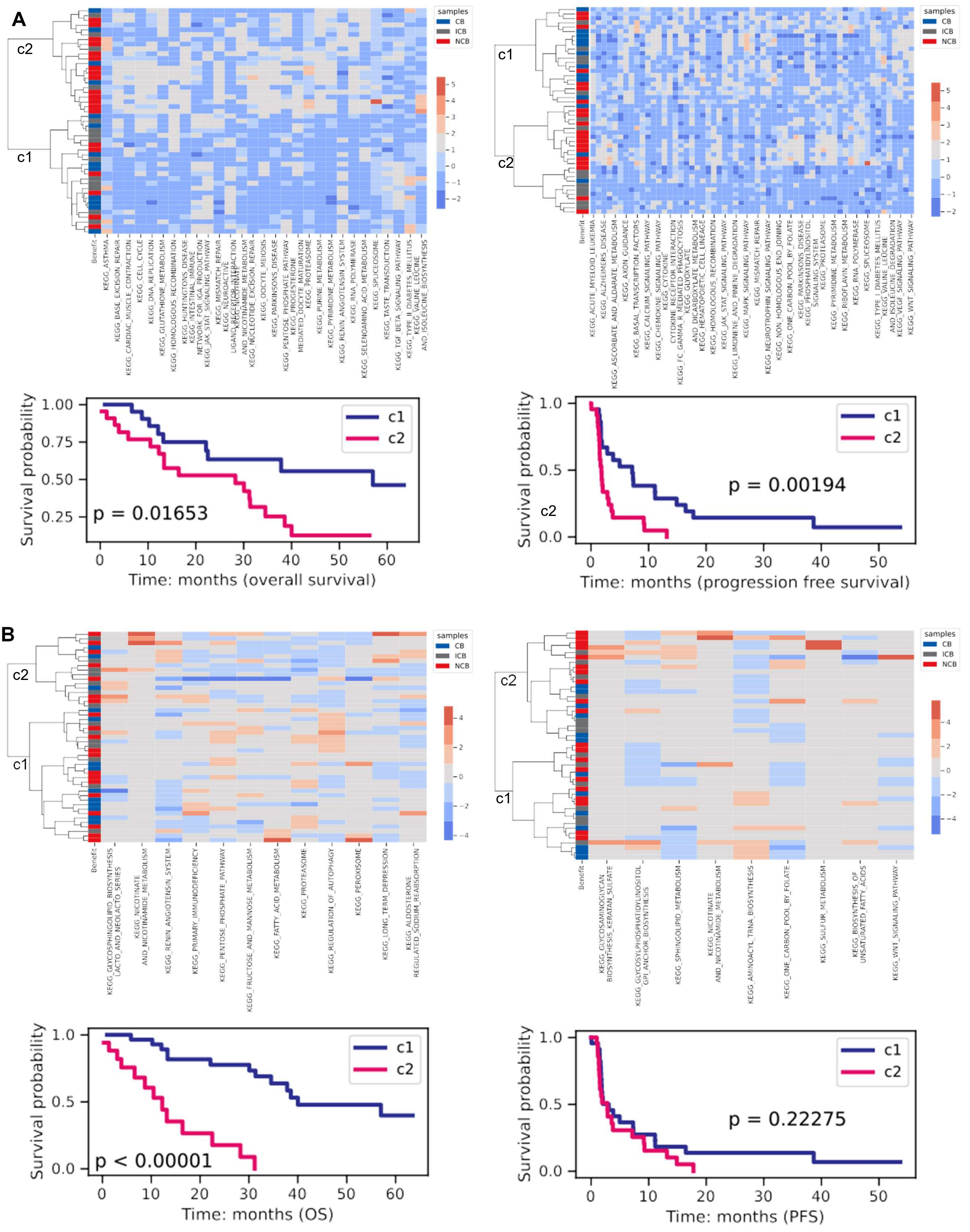
A
B
samples
CB
ICB
NCB
Benefit
c1
c2
Survival probability
Time: months (overall survival)
Time: months (progression free survival)
p = 0.01653
p = 0.00194
Time: months (OS)
Time: months (PFS)
p < 0.00001
p = 0.22275

C

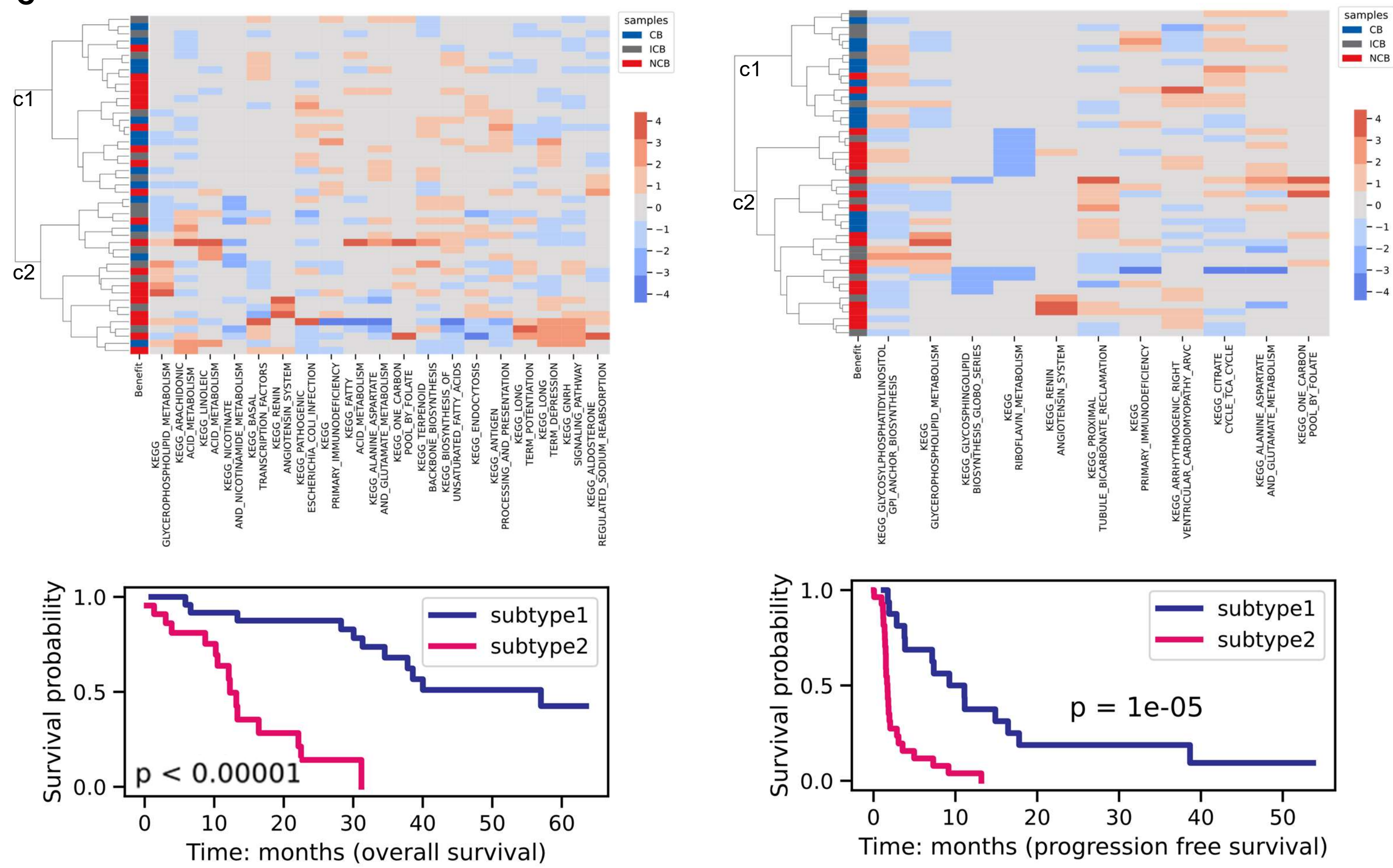

samples
CB
ICB
NCB
c1
c2
Benefit
KEGG_GLYCEROPHOSPHOLIPID_METABOLISM
KEGG_ARACHIDONIC_ACID_METABOLISM
KEGG_LINOLEIC_ACID_METABOLISM
KEGG_NICOTINATE_AND_NICOTINAMIDE_METABOLISM
KEGG_BASAL_TRANSCRIPTION_FACTORS
KEGG_RENIN_ANGIOTENSIN_SYSTEM
KEGG_PATHOGENIC_ESCHERICHIA_COLI_INFECTION
KEGG_PRIMARY_IMMUNODEFICIENCY
KEGG_FATTY_ACID_METABOLISM
KEGG_ALANINE_ASPARTATE_AND_GLUTAMATE_METABOLISM
KEGG_ONE_CARBON_POOL_BY_FOLATE
KEGG_TERPENOID_BACKBONE_BIOSYNTHESIS
KEGG_BIOSYNTHESIS_OF_UNSATURATED_FATTY_ACIDS
KEGG_ENDOCYTOSIS
KEGG_ANTIGEN_PROCESSING_AND_PRESENTATION
KEGG_LONG_TERM_POTENTIATION
KEGG_LONG_TERM_DEPRESSION
KEGG_GNRH_SIGNALING_PATHWAY
KEGG_ALDOSTERONE_REGULATED_SODIUM_REABSORPTION
KEGG_GLYCOSYLPHOSPHATIDYLINOSITOL_GPI_ANCHOR_BIOSYNTHESIS
KEGG_GLYCEROPHOSPHOLIPID_METABOLISM
KEGG_GLYCOSPHINGOLIPID_BIOSYNTHESIS_GLOBO_SERIES
KEGG_RIBOFLAVIN_METABOLISM
KEGG_RENIN_ANGIOTENSIN_SYSTEM
KEGG_PROXIMAL_TUBULE_BICARBONATE_RECLAMATION
KEGG_PRIMARY_IMMUNODEFICIENCY
KEGG_ARRHYTHMOGENIC_RIGHT_VENTRICULAR_CARDIOMYOPATHY_ARVC
KEGG_CITRATE_CYCLE_TCA_CYCLE
KEGG_ALANINE_ASPARTATE_AND_GLUTAMATE_METABOLISM
KEGG_ONE_CARBON_POOL_BY_FOLATE
Survival probability
subtype1
subtype2
p < 0.00001
Time: months (overall survival)
p = 1e-05
Time: months (progression free survival)

D

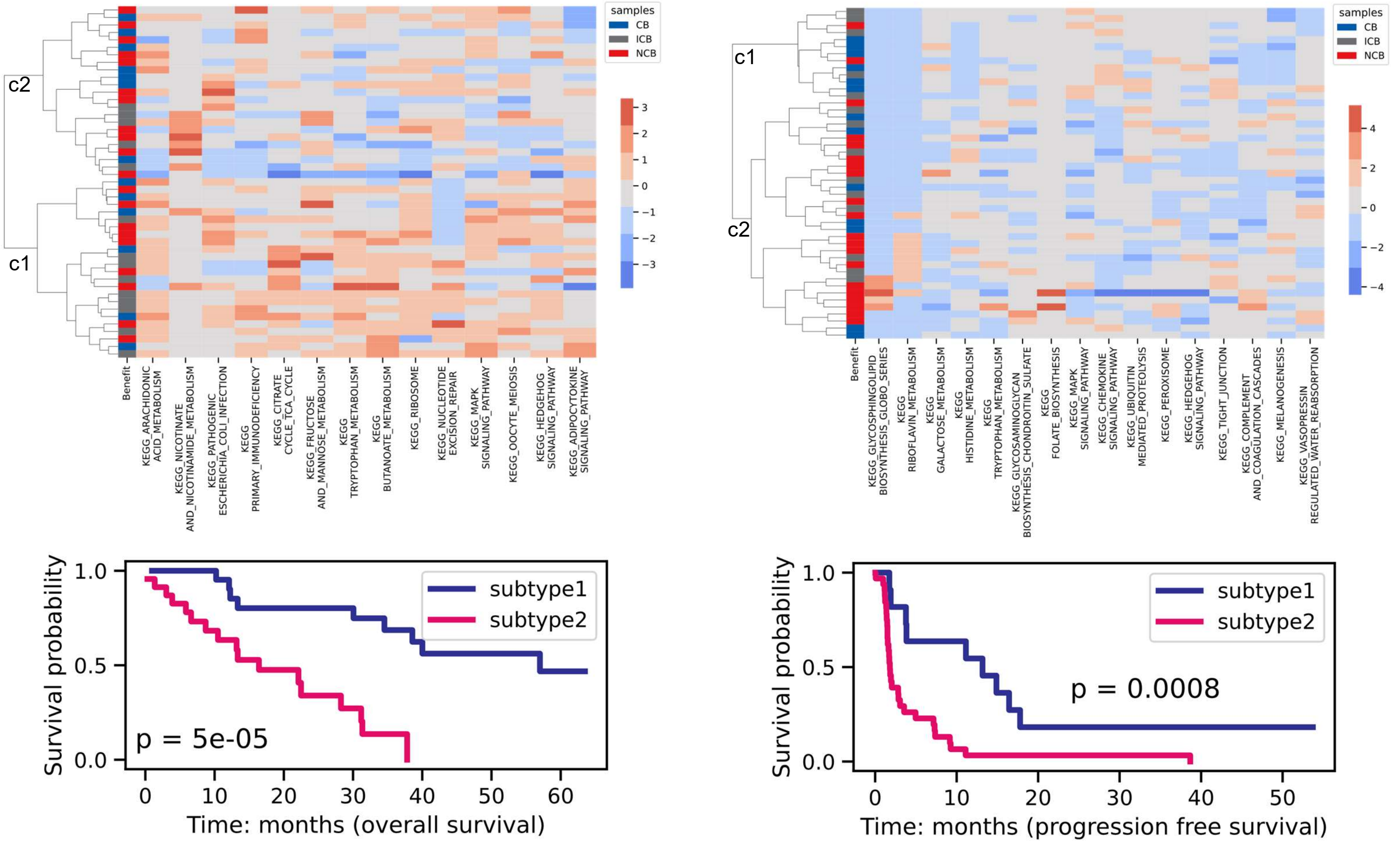

samples
CB
ICB
NCB
c1
c2
Benefit
KEGG_ARACHIDONIC_ACID_METABOLISM
KEGG_NICOTINATE_AND_NICOTINAMIDE_METABOLISM
KEGG_PATHOGENIC_ESCHERICHIA_COLI_INFECTION
KEGG_PRIMARY_IMMUNODEFICIENCY
KEGG_CITRATE_CYCLE_TCA_CYCLE
KEGG_FRUCTOSE_AND_MANNOSE_METABOLISM
KEGG_TRYPTOPHAN_METABOLISM
KEGG_BUTANOATE_METABOLISM
KEGG_RIBOSOME
KEGG_NUCLEOTIDE_EXCISION_REPAIR
KEGG_MAPK_SIGNALING_PATHWAY
KEGG_OOCYTE_MEIOSIS
KEGG_HEDGEHOG_SIGNALING_PATHWAY
KEGG_ADIPOCYTOKINE_SIGNALING_PATHWAY
KEGG_GLYCOSPHINGOLIPID_BIOSYNTHESIS_GLOBO_SERIES
KEGG_RIBOFLAVIN_METABOLISM
KEGG_GALACTOSE_METABOLISM
KEGG_HISTIDINE_METABOLISM
KEGG_TRYPTOPHAN_METABOLISM
KEGG_GLYCOSAMINOGLYCAN_BIOSYNTHESIS_CHONDROITIN_SULFATE
KEGG_FOLATE_BIOSYNTHESIS
KEGG_MAPK_SIGNALING_PATHWAY
KEGG_CHEMOKINE_SIGNALING_PATHWAY
KEGG_UBIQUITIN_MEDIATED_PROTEOLYSIS
KEGG_PEROXISOME
KEGG_HEDGEHOG_SIGNALING_PATHWAY
KEGG_TIGHT_JUNCTION
KEGG_COMPLEMENT_AND_COAGULATION_CASCADES
KEGG_MELANOGENESIS
KEGG_VASOPRESSIN_REGULATED_WATER_REABSORPTION
Survival probability
subtype1
subtype2
p = 5e-05
Time: months (overall survival)
p = 0.0008
Time: months (progression free survival)

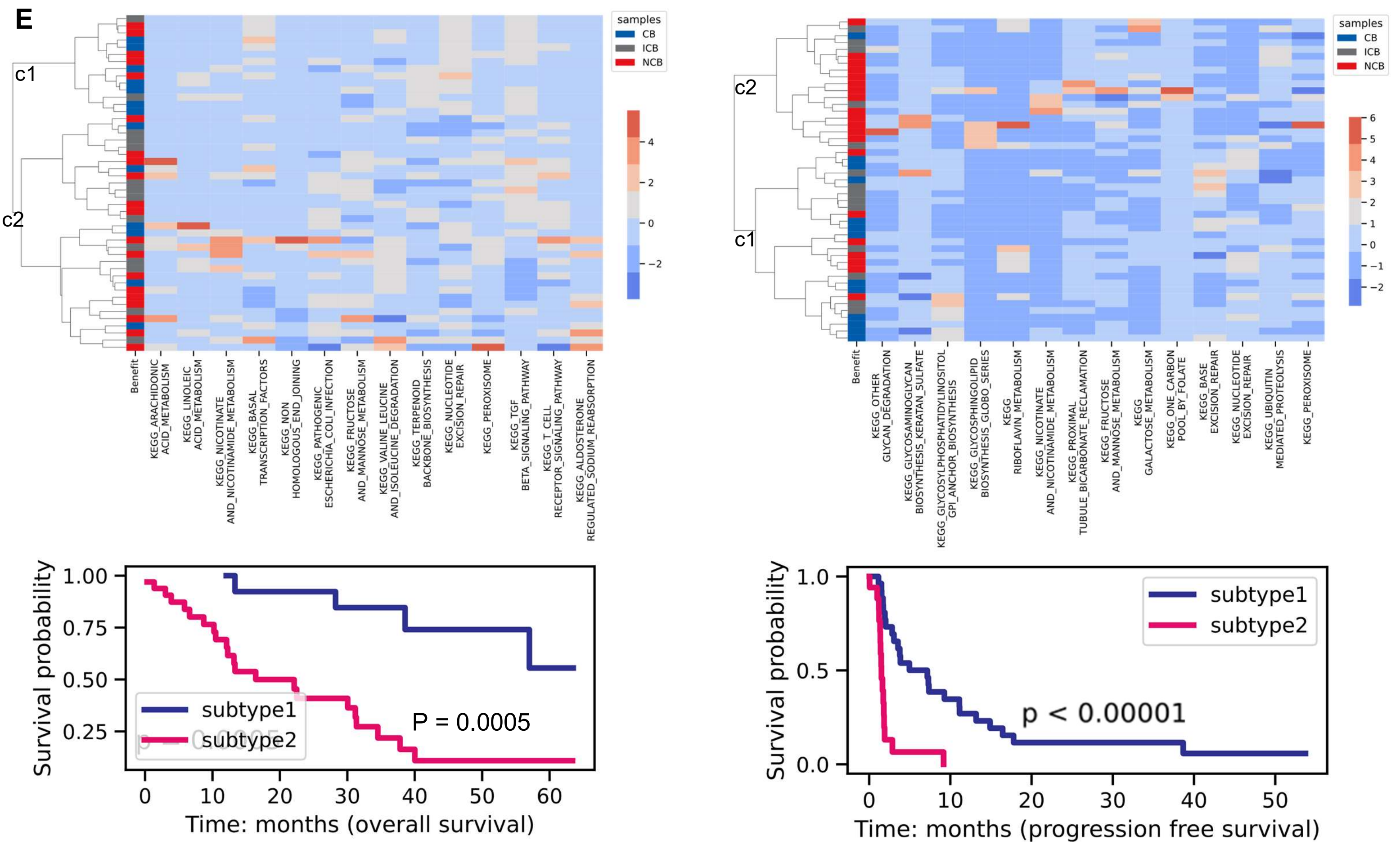

Figure S18. Clustermap and survival analysis of mN samples using pathway scores, related to Figure 5. (A). 29 (OS)/ 59 (PFS) significant pathways based on GSVA. (B). 12 (OS)/ 10 (PFS) significant pathways based on entropy. (C). 19 (OS)/ 11 (PFS) significant pathways based on gene eigenvector centrality scores. (D). 14 (OS)/ 16 (PFS) significant pathways based on gene closeness centrality scores. (E). 14 (OS)/ 14 (PFS) significant pathways based on edge betweenness centrality score.

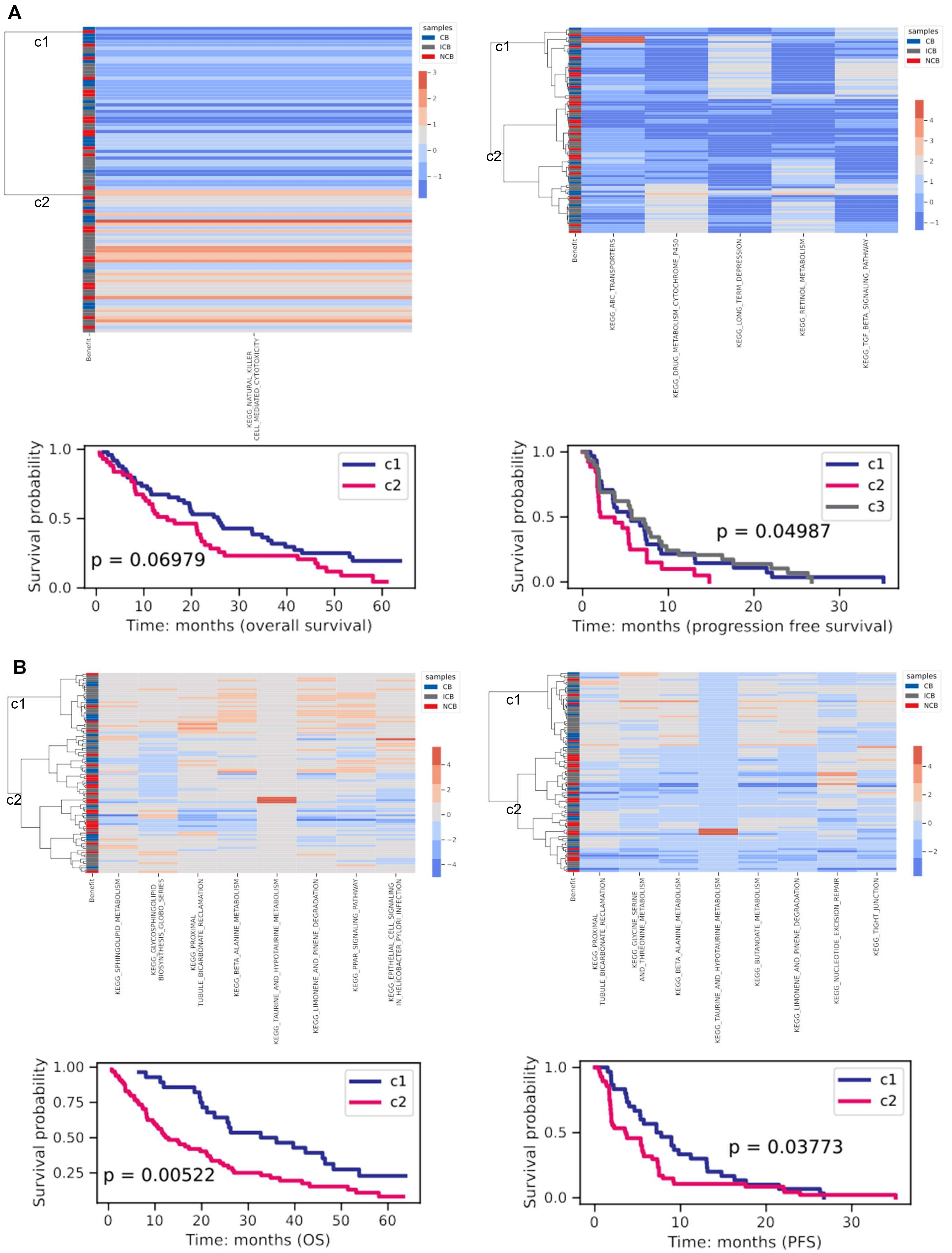

A
c1
c2
samples
CB
ICB
NCB
Benefit
KEGG_NATURAL_KILLER_CELL_MEDIATED_CYTOTOXICITY
KEGG_ABC_TRANSPORTERS
KEGG_DRUG_METABOLISM_CYTOCHROME_P450
KEGG_LONG_TERM_DEPRESSION
KEGG_RETINOL_METABOLISM
KEGG_TGF_BETA_SIGNALING_PATHWAY
Survival probability
p = 0.06979
Time: months (overall survival)
c3
p = 0.04987
Time: months (progression free survival)
B
KEGG_SPHINGOLIPID_METABOLISM
KEGG_GLYCOSPHINGOLIPID_BIOSYNTHESIS_GLOBO_SERIES
KEGG_PROXIMAL_TUBULE_BICARBONATE_RECLAMATION
KEGG_BETA_ALANINE_METABOLISM
KEGG_TAURINE_AND_HYPOTAURINE_METABOLISM
KEGG_LIMONENE_AND_PINENE_DEGRADATION
KEGG_PPAR_SIGNALING_PATHWAY
KEGG_EPITHELIAL_CELL_SIGNALING_IN_HELICOBACTER_PYLORI_INFECTION
KEGG_GLYCINE_SERINE_AND_THREONINE_METABOLISM
KEGG_BUTANOATE_METABOLISM
KEGG_NUCLEOTIDE_EXCISION_REPAIR
KEGG_TIGHT_JUNCTION
p = 0.00522
Time: months (OS)
p = 0.03773
Time: months (PFS)

C

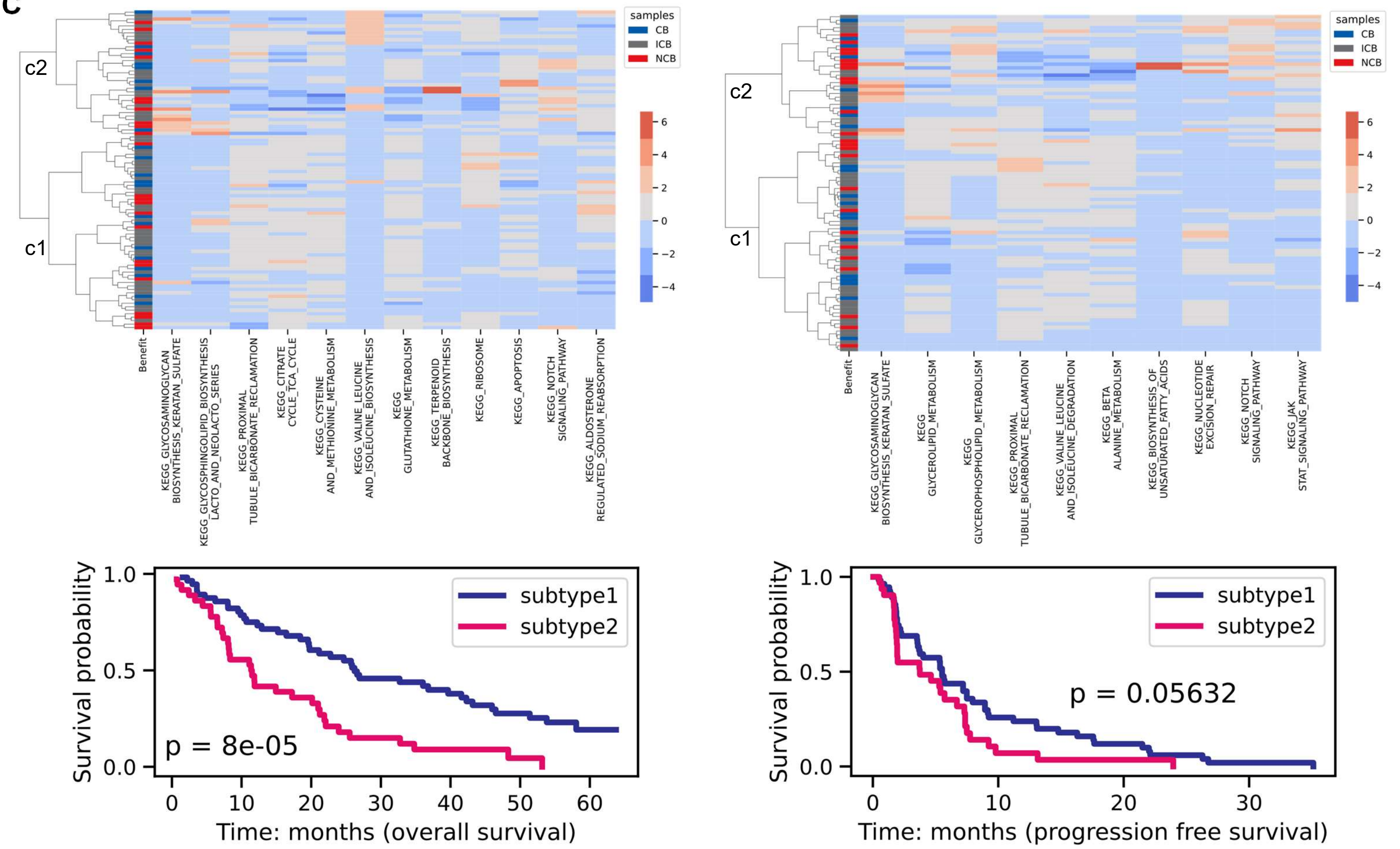
c2
c1
samples
CB
ICB
NCB
6
4
2
0
−2
−4
Benefit
KEGG_GLYCOSAMINOGLYCAN BIOSYNTHESIS_KERATAN_SULFATE
KEGG_GLYCOSPHINGOLIPID_BIOSYNTHESIS LACTO_AND_NEOLACTO_SERIES
KEGG_PROXIMAL TUBULE_BICARBONATE_RECLAMATION
KEGG_CITRATE CYCLE_TCA_CYCLE
KEGG_CYSTEINE AND_METHIONINE_METABOLISM
KEGG_VALINE_LEUCINE AND_ISOLEUCINE_BIOSYNTHESIS
KEGG GLUTATHIONE_METABOLISM
KEGG_TERPENOID BACKBONE_BIOSYNTHESIS
KEGG_RIBOSOME
KEGG_APOPTOSIS
KEGG_NOTCH SIGNALING_PATHWAY
KEGG_ALDOSTERONE REGULATED_SODIUM_REABSORPTION
KEGG_GLYCOSAMINOGLYCAN BIOSYNTHESIS_KERATAN_SULFATE
KEGG GLYCEROLIPID_METABOLISM
KEGG GLYCEROPHOSPHOLIPID_METABOLISM
KEGG_PROXIMAL TUBULE_BICARBONATE_RECLAMATION
KEGG_VALINE_LEUCINE AND_ISOLEUCINE_DEGRADATION
KEGG_BETA ALANINE_METABOLISM
KEGG_BIOSYNTHESIS_OF UNSATURATED_FATTY_ACIDS
KEGG_NUCLEOTIDE EXCISION_REPAIR
KEGG_NOTCH SIGNALING_PATHWAY
KEGG_JAK STAT_SIGNALING_PATHWAY
Survival probability
subtype1
subtype2
p = 8e-05
Time: months (overall survival)
p = 0.05632
Time: months (progression free survival)

D

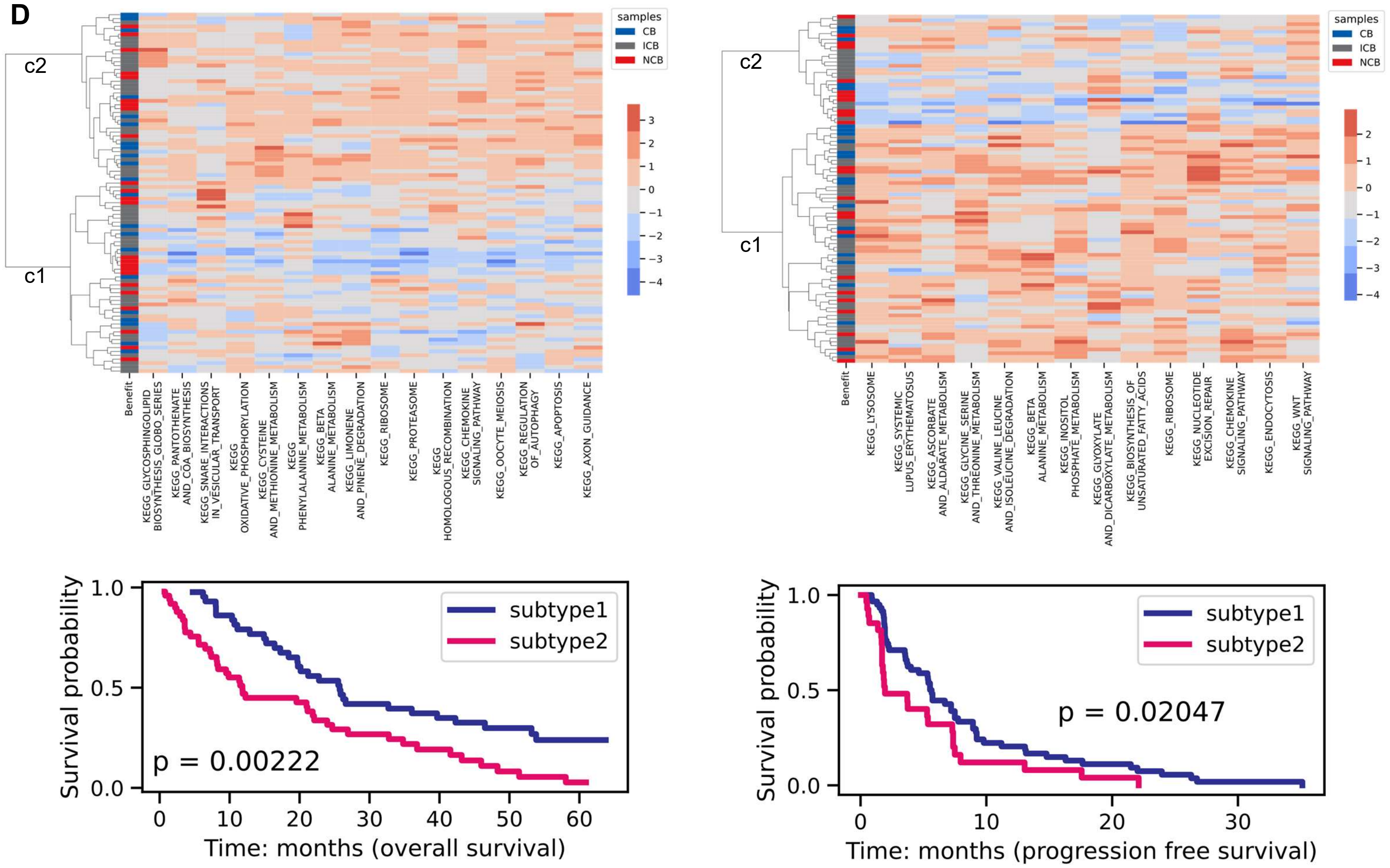
c2
c1
samples
CB
ICB
NCB
3
2
1
0
−1
−2
−3
−4
Benefit
KEGG_GLYCOSPHINGOLIPID BIOSYNTHESIS_GLOBO_SERIES
KEGG_PANTOTHENATE AND_COA_BIOSYNTHESIS
KEGG_SNARE_INTERACTIONS IN_VESICULAR_TRANSPORT
KEGG OXIDATIVE_PHOSPHORYLATION
KEGG_CYSTEINE AND_METHIONINE_METABOLISM
KEGG PHENYLALANINE_METABOLISM
KEGG_BETA ALANINE_METABOLISM
KEGG_LIMONENE AND_PINENE_DEGRADATION
KEGG_RIBOSOME
KEGG_PROTEASOME
KEGG HOMOLOGOUS_RECOMBINATION
KEGG_CHEMOKINE SIGNALING_PATHWAY
KEGG_OOCYTE_MEIOSIS
KEGG_REGULATION OF_AUTOPHAGY
KEGG_APOPTOSIS
KEGG_AXON_GUIDANCE
KEGG_LYSOSOME
KEGG_SYSTEMIC LUPUS_ERYTHEMATOSUS
KEGG_ASCORBATE AND_ALDARATE_METABOLISM
KEGG_GLYCINE_SERINE AND_THREONINE_METABOLISM
KEGG_VALINE_LEUCINE AND_ISOLEUCINE_DEGRADATION
KEGG_BETA ALANINE_METABOLISM
KEGG_INOSITOL PHOSPHATE_METABOLISM
KEGG_GLYOXYLATE AND_DICARBOXYLATE_METABOLISM
KEGG_BIOSYNTHESIS_OF UNSATURATED_FATTY_ACIDS
KEGG_RIBOSOME
KEGG_NUCLEOTIDE EXCISION_REPAIR
KEGG_CHEMOKINE SIGNALING_PATHWAY
KEGG_ENDOCYTOSIS
KEGG_WNT SIGNALING_PATHWAY
Survival probability
subtype1
subtype2
p = 0.00222
Time: months (overall survival)
p = 0.02047
Time: months (progression free survival)

E

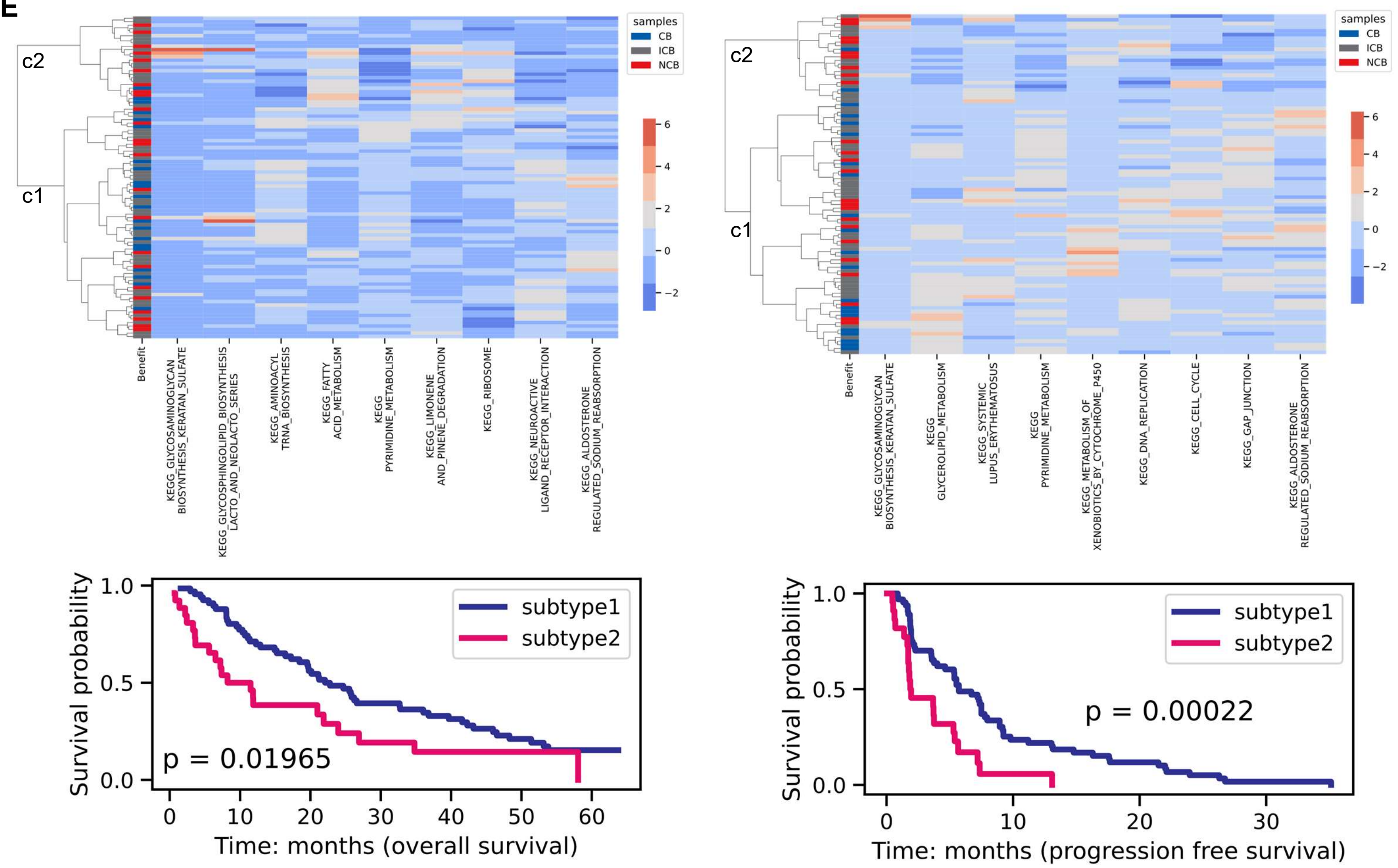

Figure S19. Clustermap and survival analysis of pE samples using pathway scores, related to Figure 5. (A). 1 (OS)/ 5 (PFS) significant pathways based on GSVA. (B). 8 (OS)/ 8 (PFS) significant pathways based on entropy. (C). 12 (OS)/ 10 (PFS) significant pathways based on gene eigenvector centrality scores. (D). 16 (OS)/ 14 (PFS) significant pathways based on gene closeness centrality scores. (E). 9 (OS)/ 9 (PFS) significant pathways based on edge betweenness centrality score.

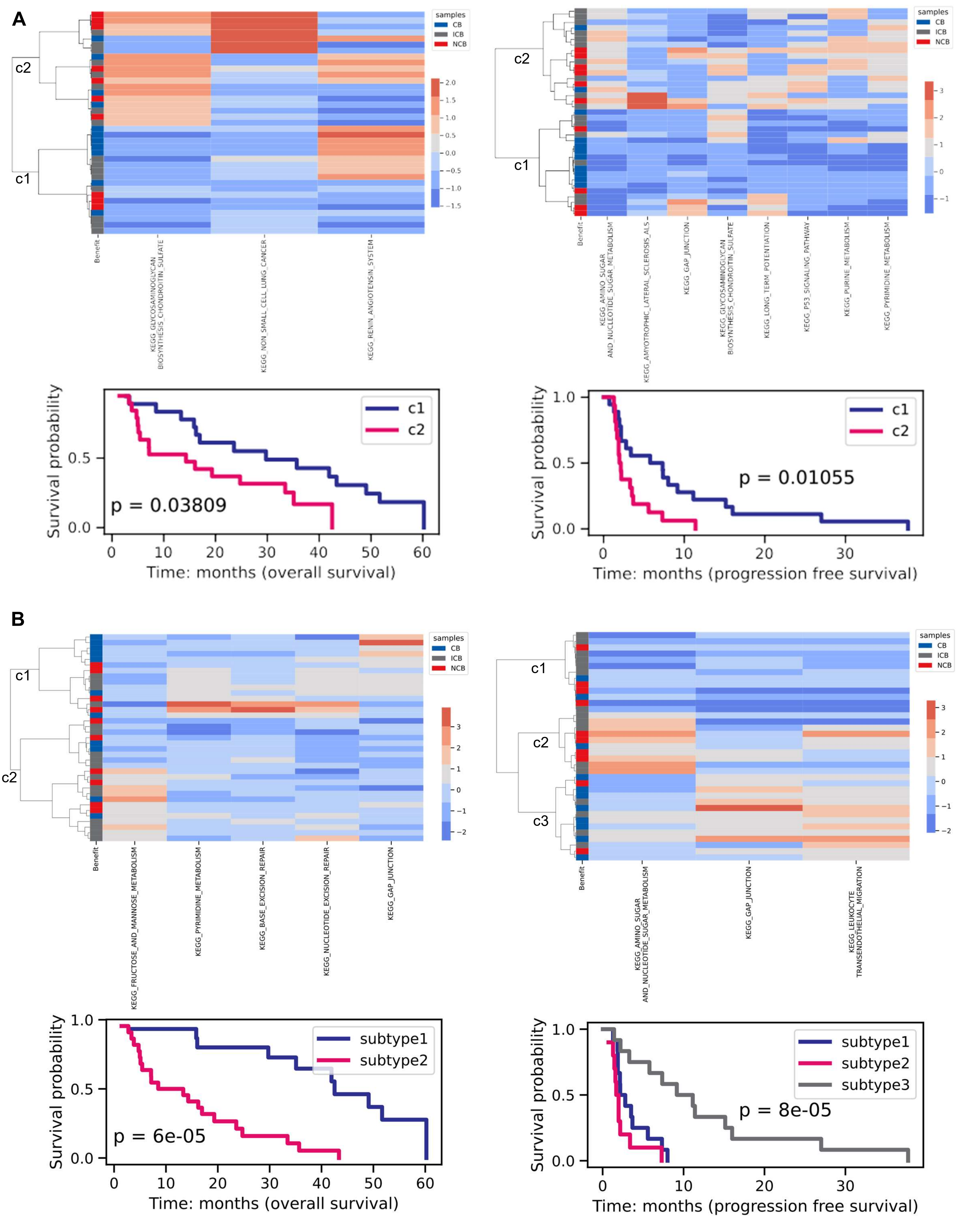

A
c2
c1
samples
CB
ICB
NCB
Benefit
KEGG_GLYCOSAMINOGLYCAN_BIOSYNTHESIS_CHONDROITIN_SULFATE
KEGG_NON_SMALL_CELL_LUNG_CANCER
KEGG_RENIN_ANGIOTENSIN_SYSTEM
KEGG_AMINO_SUGAR_AND_NUCLEOTIDE_SUGAR_METABOLISM
KEGG_AMYOTROPHIC_LATERAL_SCLEROSIS_ALS
KEGG_GAP_JUNCTION
KEGG_GLYCOSAMINOGLYCAN_BIOSYNTHESIS_CHONDROITIN_SULFATE
KEGG_LONG_TERM_POTENTIATION
KEGG_P53_SIGNALING_PATHWAY
KEGG_PURINE_METABOLISM
KEGG_PYRIMIDINE_METABOLISM
Survival probability
p = 0.03809
Time: months (overall survival)
p = 0.01055
Time: months (progression free survival)
B
KEGG_FRUCTOSE_AND_MANNOSE_METABOLISM
KEGG_PYRIMIDINE_METABOLISM
KEGG_BASE_EXCISION_REPAIR
KEGG_NUCLEOTIDE_EXCISION_REPAIR
KEGG_GAP_JUNCTION
c3
KEGG_AMINO_SUGAR_AND_NUCLEOTIDE_SUGAR_METABOLISM
KEGG_GAP_JUNCTION
KEGG_LEUKOCYTE_TRANSENDOTHELIAL_MIGRATION
subtype1
subtype2
subtype3
p = 6e-05
p = 8e-05

C

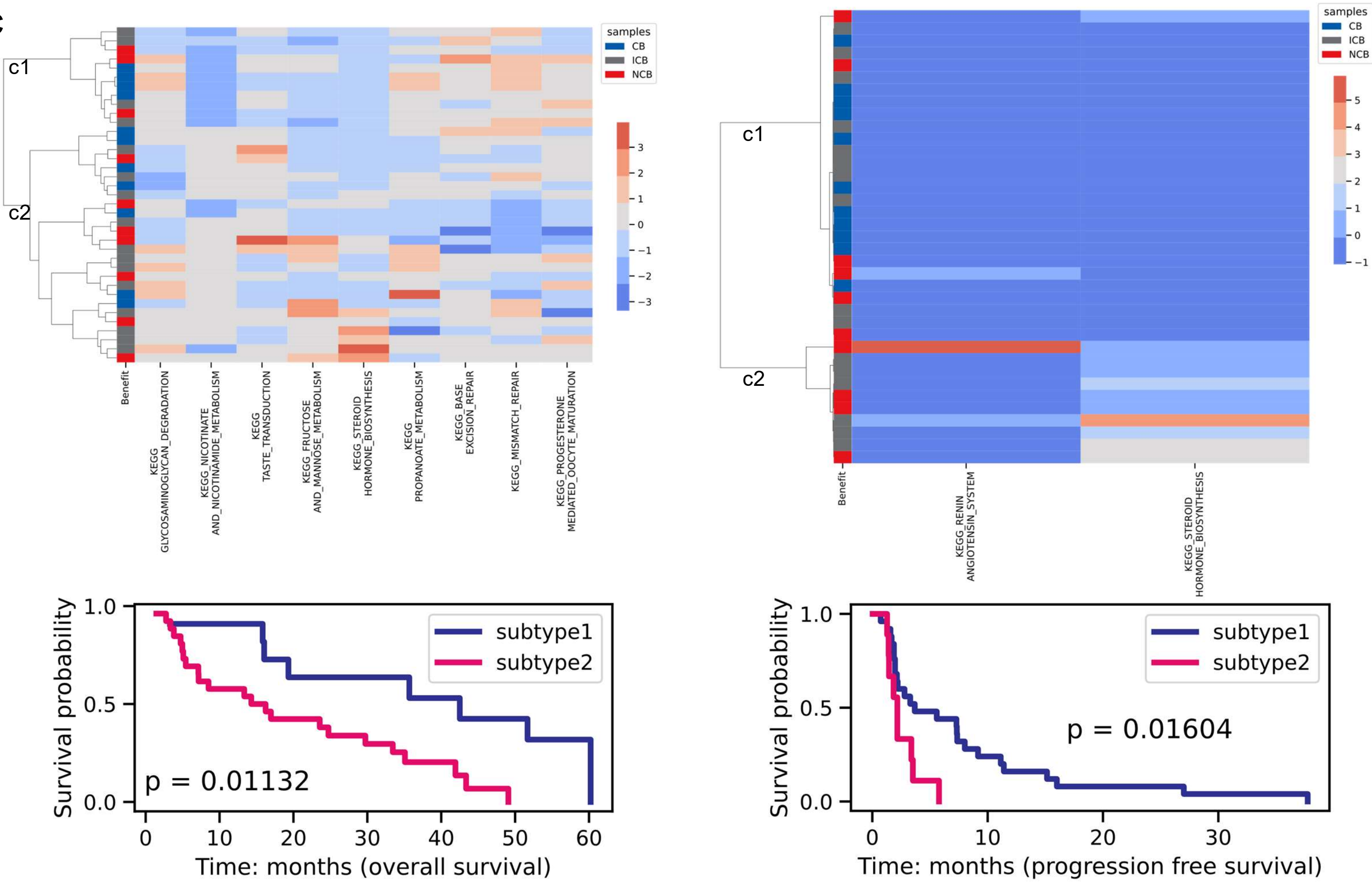
c1
c2
samples
CB
ICB
NCB
Benefit
KEGG GLYCOSAMINOGLYCAN_DEGRADATION
KEGG_NICOTINATE AND_NICOTINAMIDE_METABOLISM
KEGG TASTE_TRANSDUCTION
KEGG_FRUCTOSE AND_MANNOSE_METABOLISM
KEGG_STEROID HORMONE_BIOSYNTHESIS
KEGG PROPANOATE_METABOLISM
KEGG_BASE EXCISION_REPAIR
KEGG_MISMATCH_REPAIR
KEGG_PROGESTERONE MEDIATED_OOCYTE_MATURATION
KEGG_RENIN ANGIOTENSIN_SYSTEM
subtype1
subtype2
Survival probability
p = 0.01132
Time: months (overall survival)
p = 0.01604
Time: months (progression free survival)

D

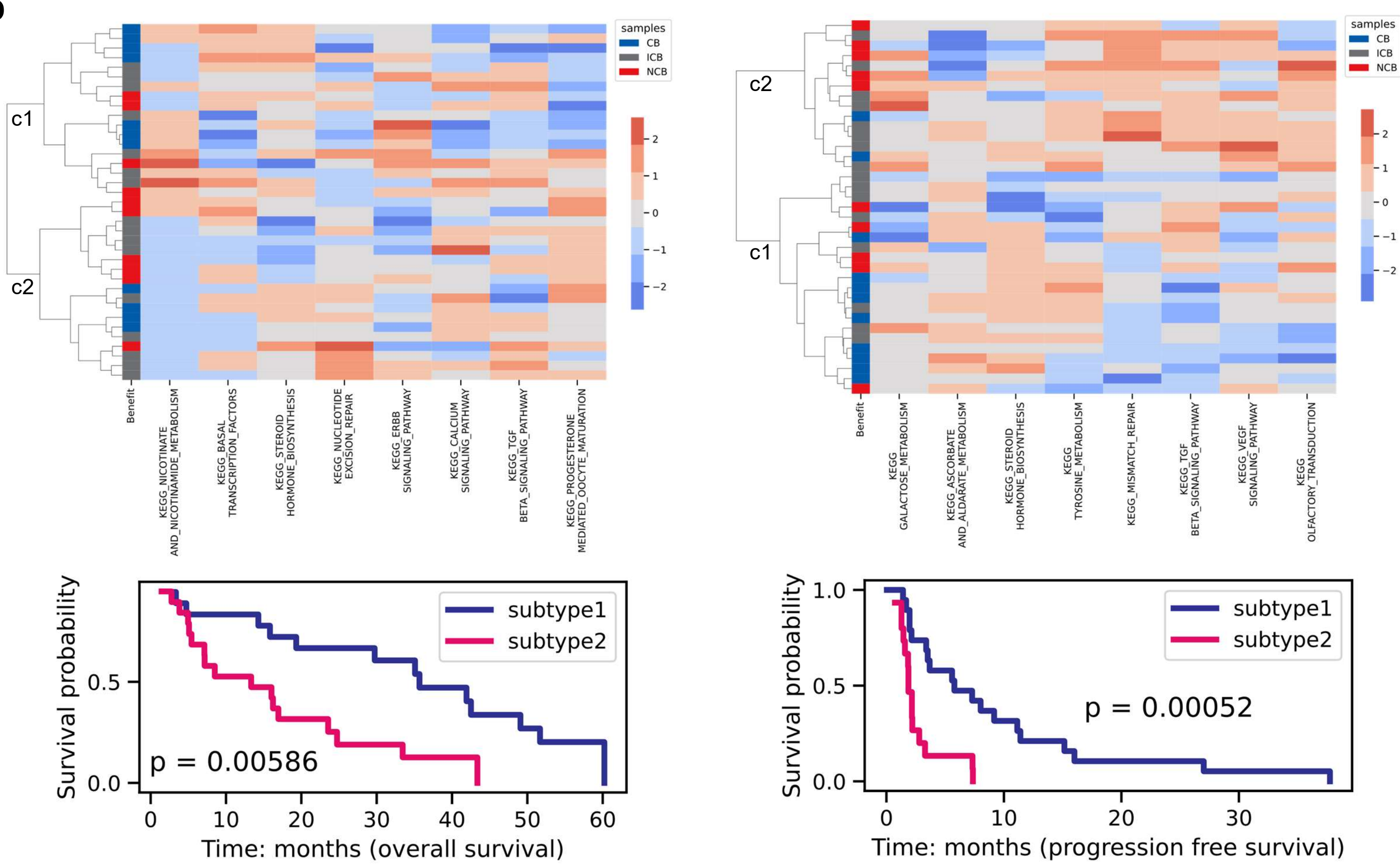
c1
c2
samples
CB
ICB
NCB
Benefit
KEGG_NICOTINATE AND_NICOTINAMIDE_METABOLISM
KEGG_BASAL TRANSCRIPTION_FACTORS
KEGG_STEROID HORMONE_BIOSYNTHESIS
KEGG_NUCLEOTIDE EXCISION_REPAIR
KEGG_ERBB SIGNALING_PATHWAY
KEGG_CALCIUM SIGNALING_PATHWAY
KEGG_TGF BETA_SIGNALING_PATHWAY
KEGG_PROGESTERONE MEDIATED_OOCYTE_MATURATION
KEGG GALACTOSE_METABOLISM
KEGG_ASCORBATE AND_ALDARATE_METABOLISM
KEGG TYROSINE_METABOLISM
KEGG_MISMATCH_REPAIR
KEGG_VEGF SIGNALING_PATHWAY
KEGG OLFACTORY_TRANSDUCTION
subtype1
subtype2
Survival probability
p = 0.00586
Time: months (overall survival)
p = 0.00052
Time: months (progression free survival)

E

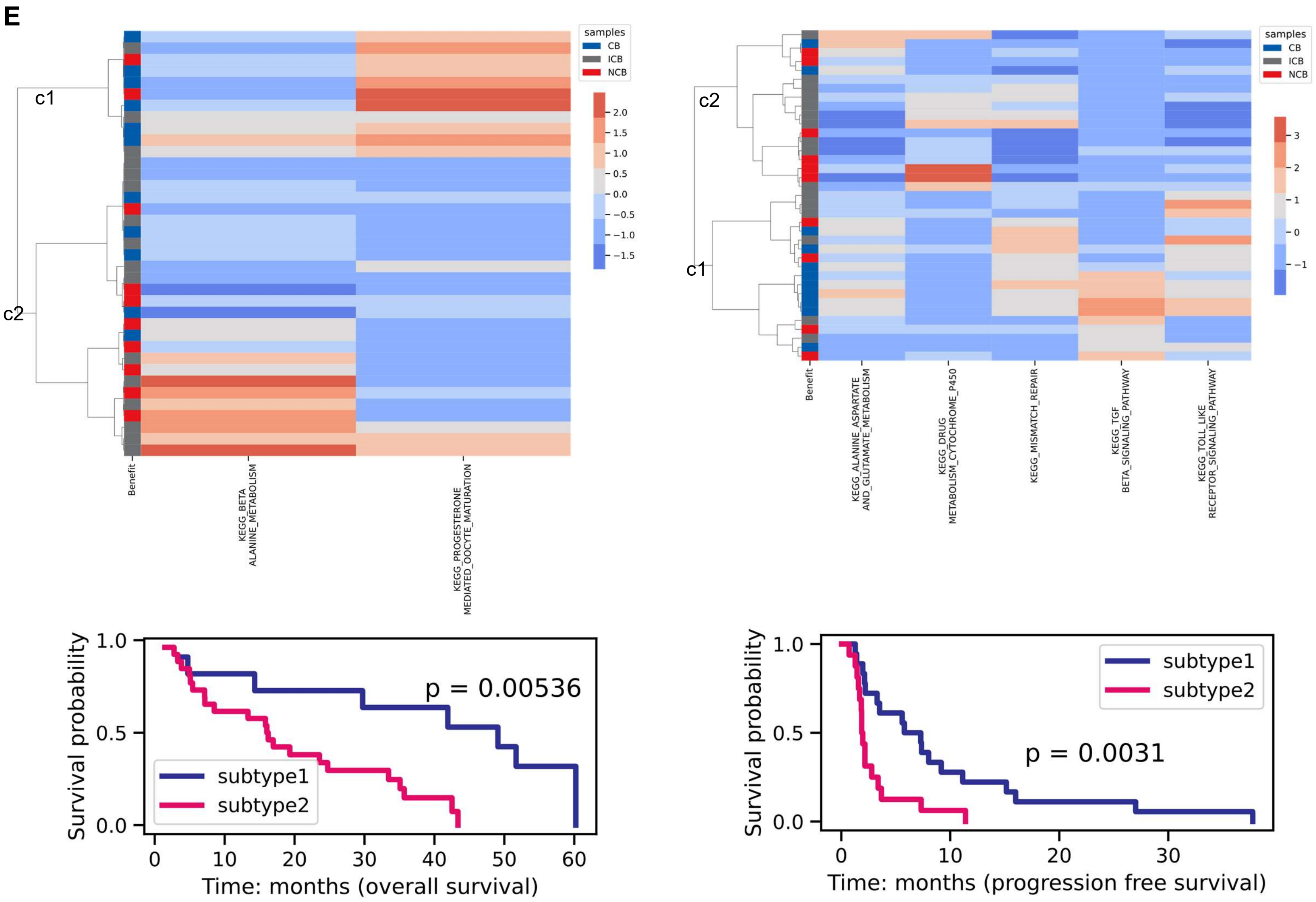

Figure S20. Clustermap and survival analysis of mE samples using pathway scores, related to Figure 5. (A). 3 (OS)/ 8 (PFS) significant pathways based on GSVA. (B). 5 (OS)/ 3 (PFS) significant pathways based on entropy. (C). 9 (OS)/ 2 (PFS) significant pathways based on gene eigenvector centrality scores. (D). 8 (OS)/ 8 (PFS) significant pathways based on gene closeness centrality scores. (E). 2 (OS)/ 5 (PFS) significant pathways based on edge betweenness centrality score.

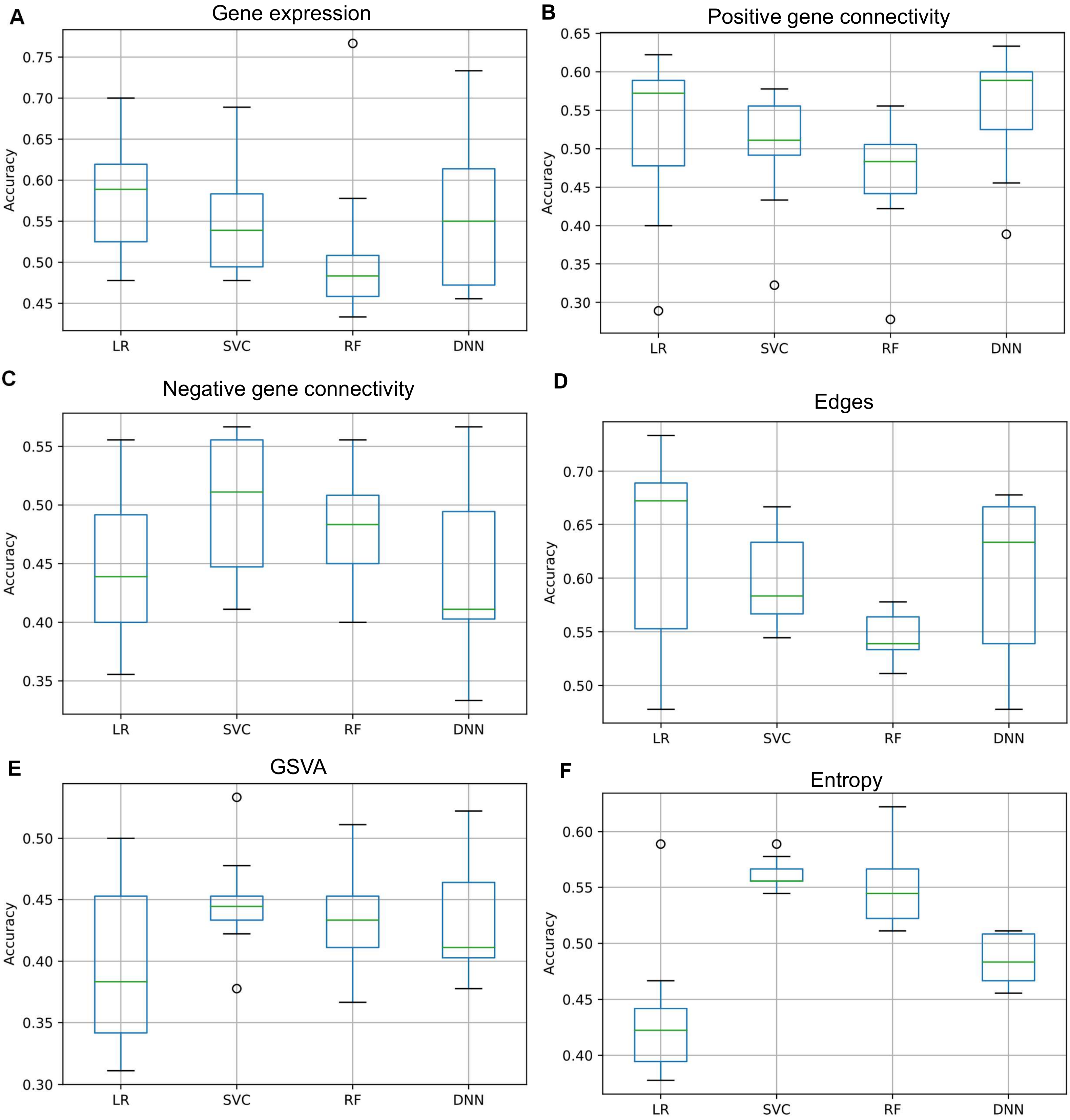
A
Gene expression
B
Positive gene connectivity
C
Negative gene connectivity
D
Edges
E
GSVA
F
Entropy
Accuracy
LR
SVC
RF
DNN

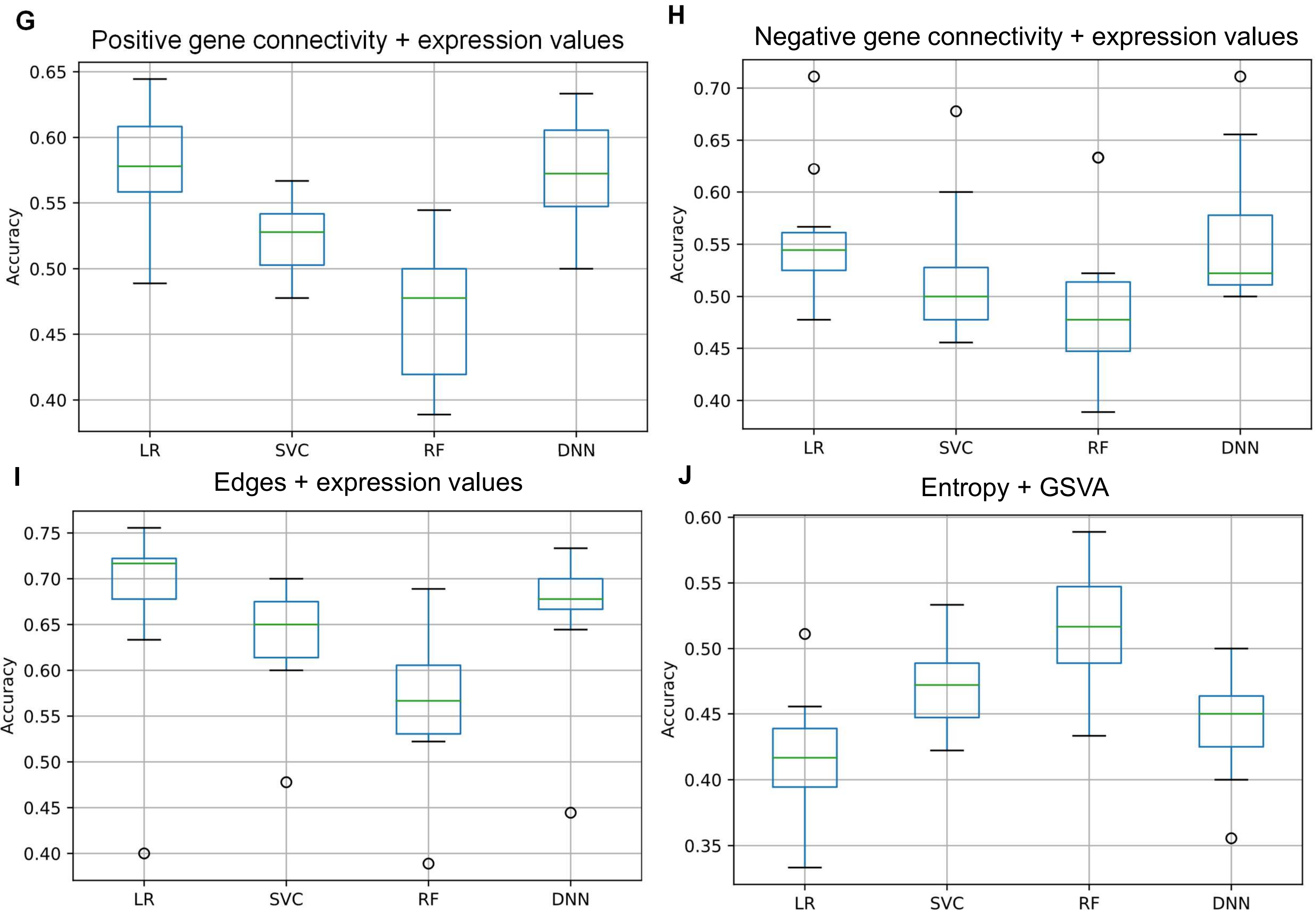

Figure S21. Comparisons of leave-one-out cross validation (LOOCV) prediction performance of four ML models based on gene expression input, network feature input and their combinations, related to Figure 6. LR: Logistic regression model. RF: Random forest model. SVC: Support vector machine model. DNN: deep neural network model. (A). Gene expression. (B). Positive gene connectivity. (C). Negative gene connectivity. (D). Edges. (E). GSVA. (F). Entropy. (G). Positive gene connectivity + expression values. (H). Negative gene connectivity + expression values. (I). Edges and expression values. (J). Entropy and GSVA.

For each algorithm-based ML model, we ran ML models ten times independently by using different feature numbers (K) from 10 to 100 with a 10 step, embedded in the selectKbest function with f_classif.

,

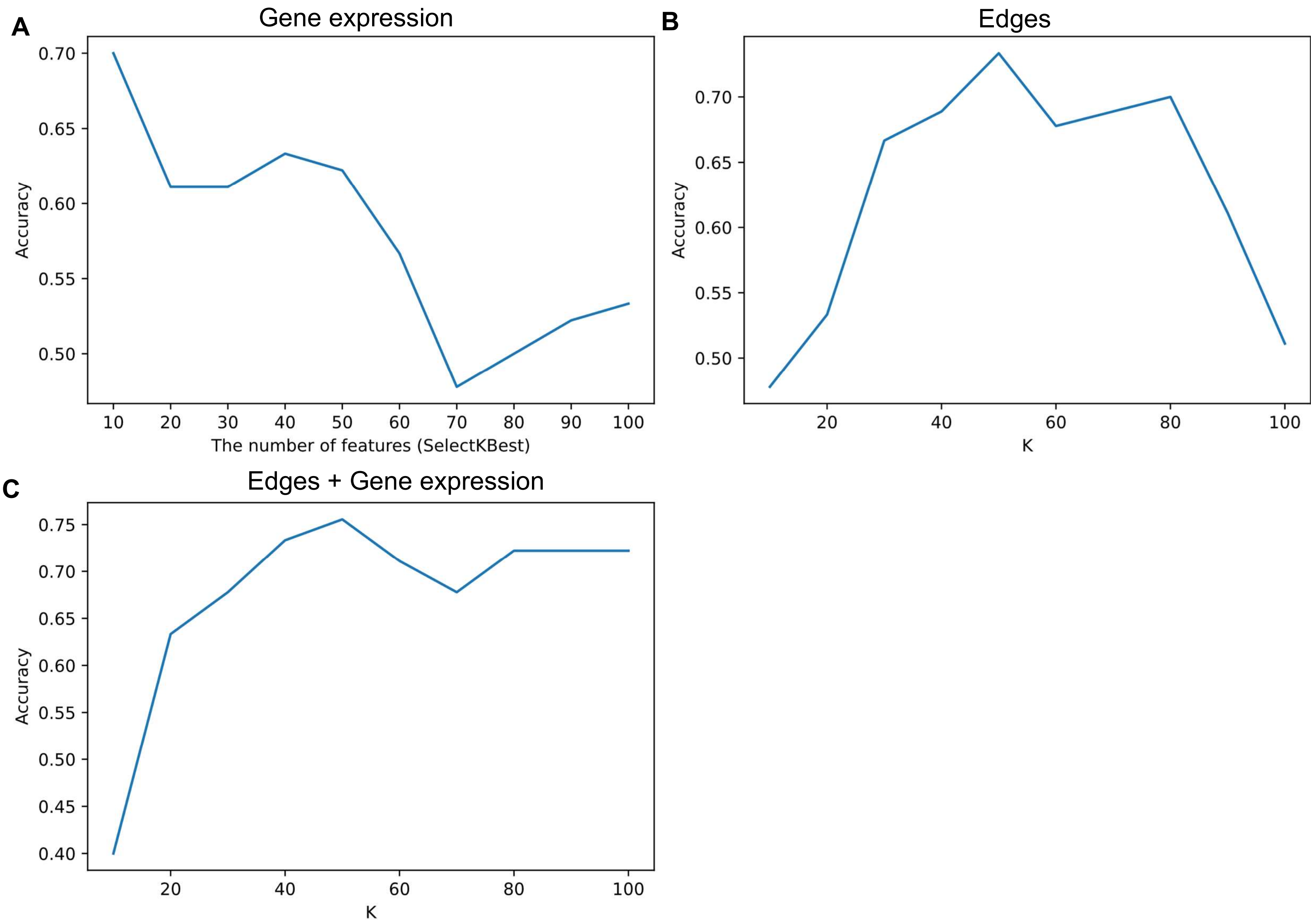

Figure S22. Selection of feature number for LOOCV - logistical regression prediction models, related to Figure 6. We used f_classif from selectKbest function to test which number of features could achieve the highest prediction accuracy. The number of features was set from 10 to 100 with a 10 step. (A). Gene expression as the input. (B). Edges as the input. (C). The combination of edges and gene expression as the input.

We selected the most 10, 50, 50 important features from the training set with the input of gene expression, edges, and the combination of gene expression and edges.

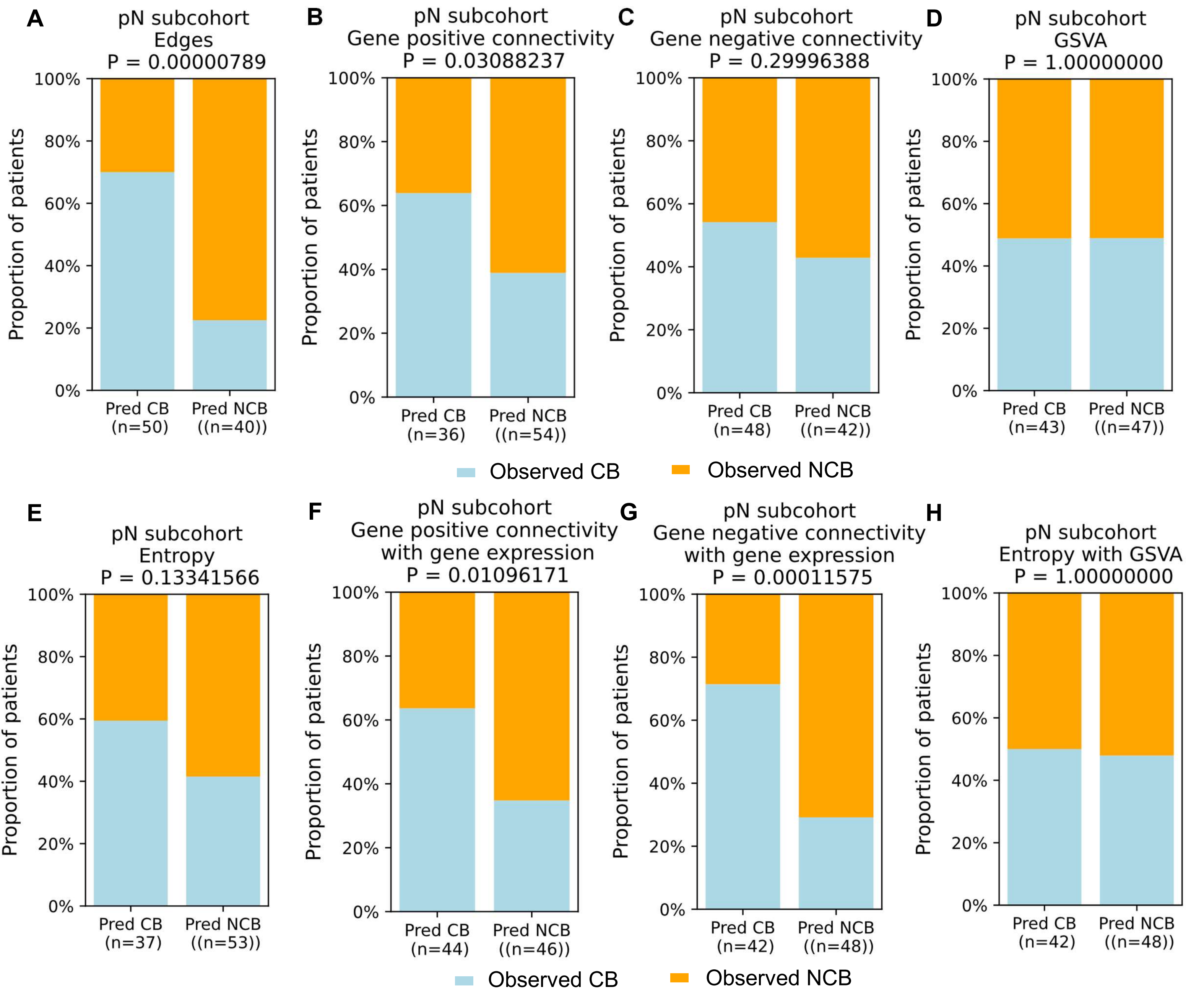

Figure S23. Bar plot of predicted responders and non-responders from LOOCV LR prediction models, related to Figure 6. Predicted responders (Pred CB) and predicted non-responders (Pred NCB) are plotted against real responders (light blue) and non-responders (orange). (A), 50 features for edges. (B), 90 features for positive gene connectivity (C), 30 features for negative gene connectivity (D), 10 features for GSVA. (E), 10 features for pathway entropy. (F), 10 features for positive gene connectivity and gene expression. (G), 10 features for negative gene connectivity and gene expression. (H). 10 features for pathway entropy and GSVA.

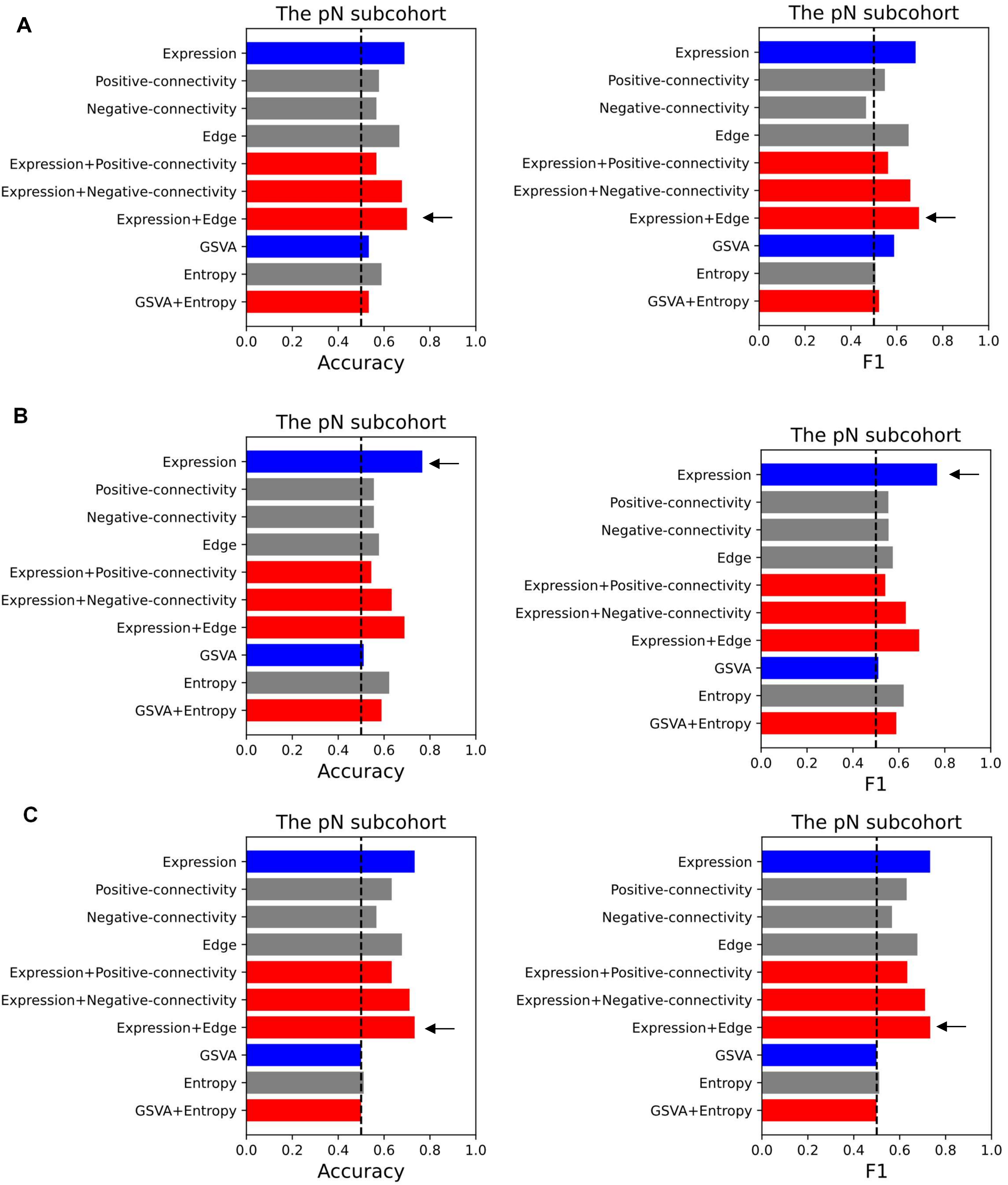

Figure S24. Leave-one-out cross validation (LOOCV) method evaluated the performance (accuracy and F1 score) of ML models based on gene expression input, network feature input and their combination, related to Figure 6. (A). Support vector machine (SVC) model. (B). Random forest (RF) model. (C). Deep neural network (DNN). The highest scores were highlighted.

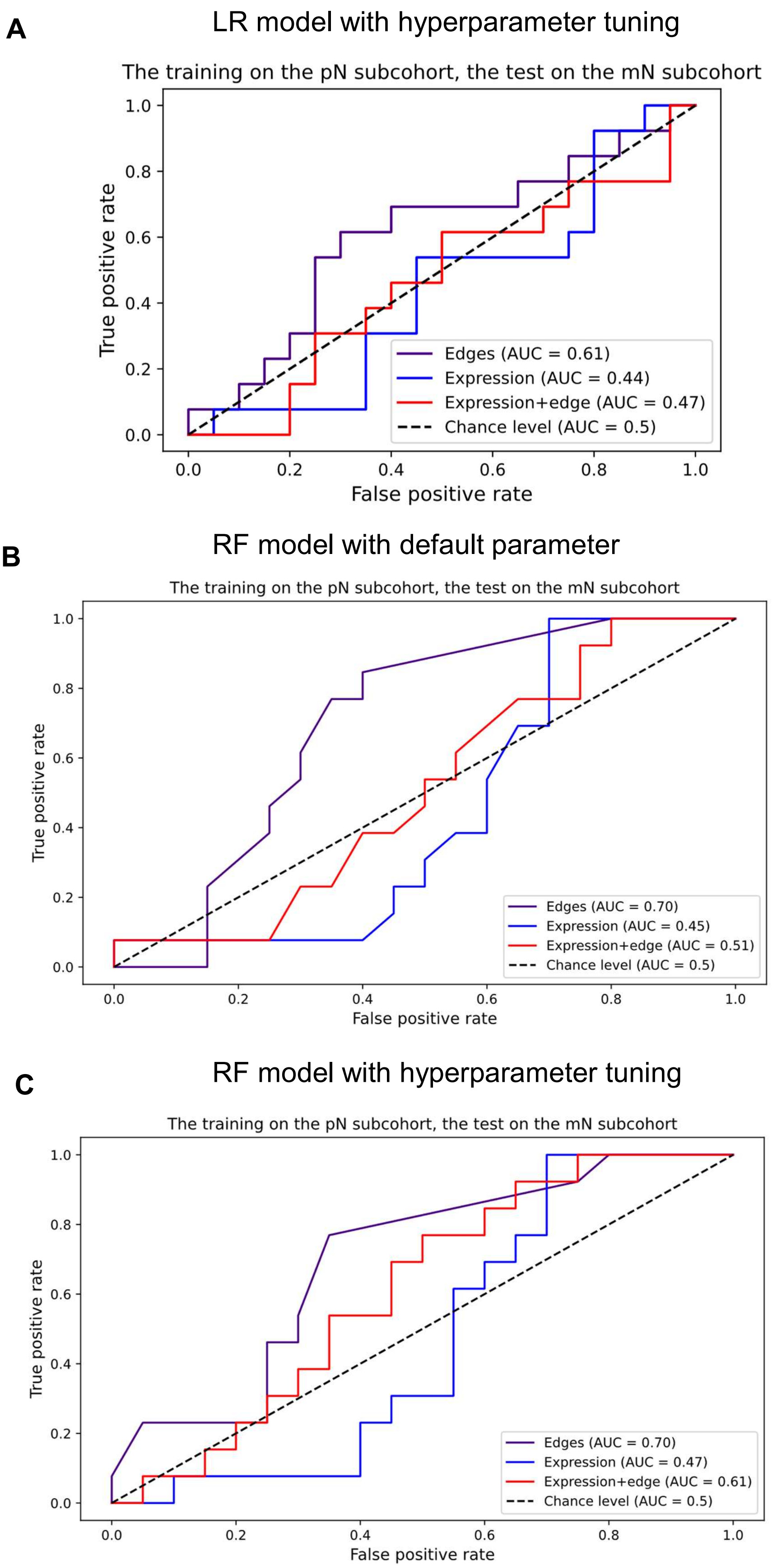

Figure S25. Across study prediction, related to Figure 6. The pN subcohort (n = 90) were used as the training set (20 features selected for edges, 30 features for both expression, and 50 features for their combination during the training process), and the mN subcohort was used as the test set (n = 33). (A). Logistic regression classification model with hyperparameter tuning. (B). Random forest model with default parameters. (C). Random forest model with hyperparameter tuning.

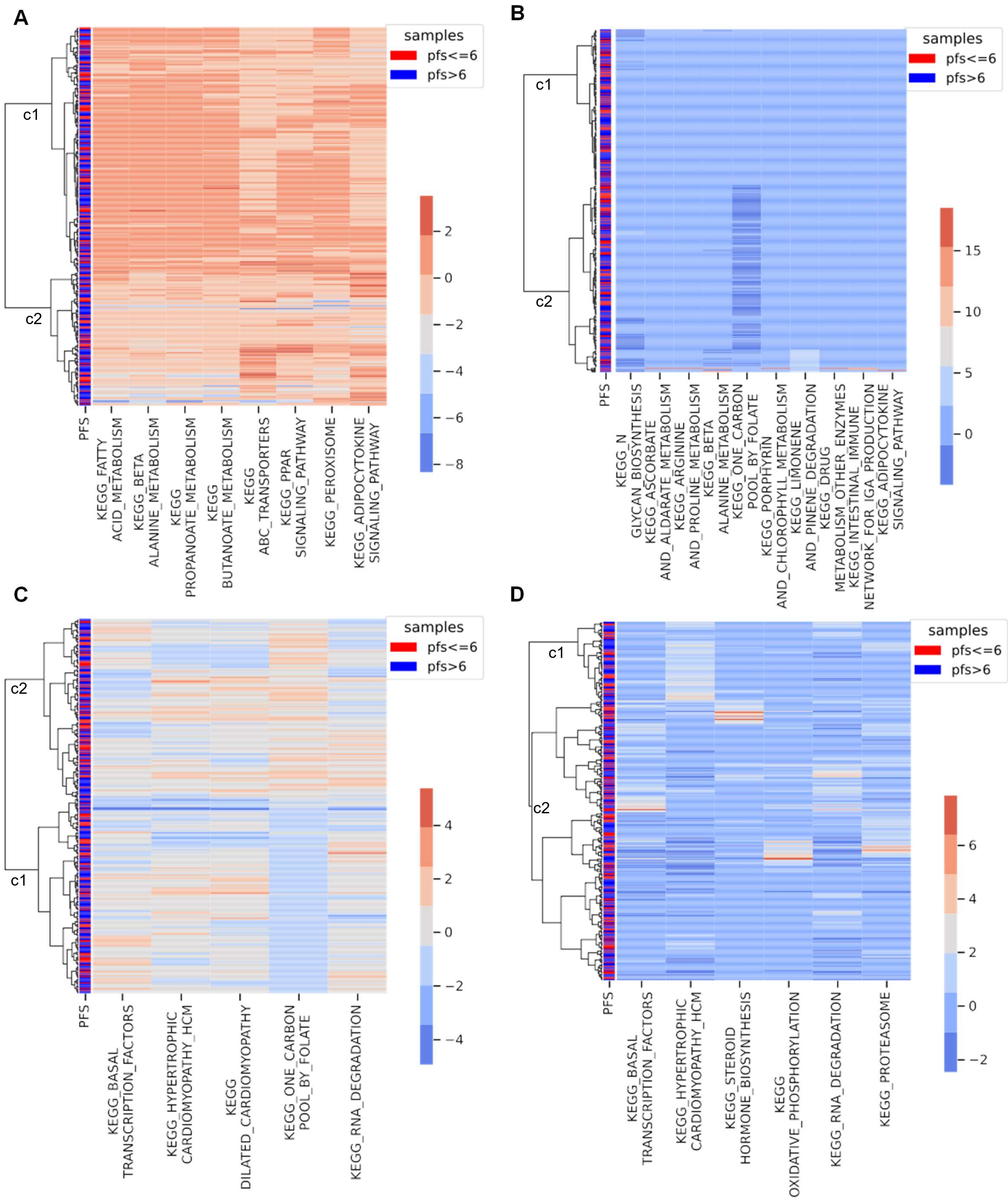

Figure S26. Clustering samples treated with Avelumab plus axitinib, related to Figure 7. A. Entropy. B. Eigenvector centrality scores. C. Closeness centrality scores. D. Edge betweenness centrality scores.

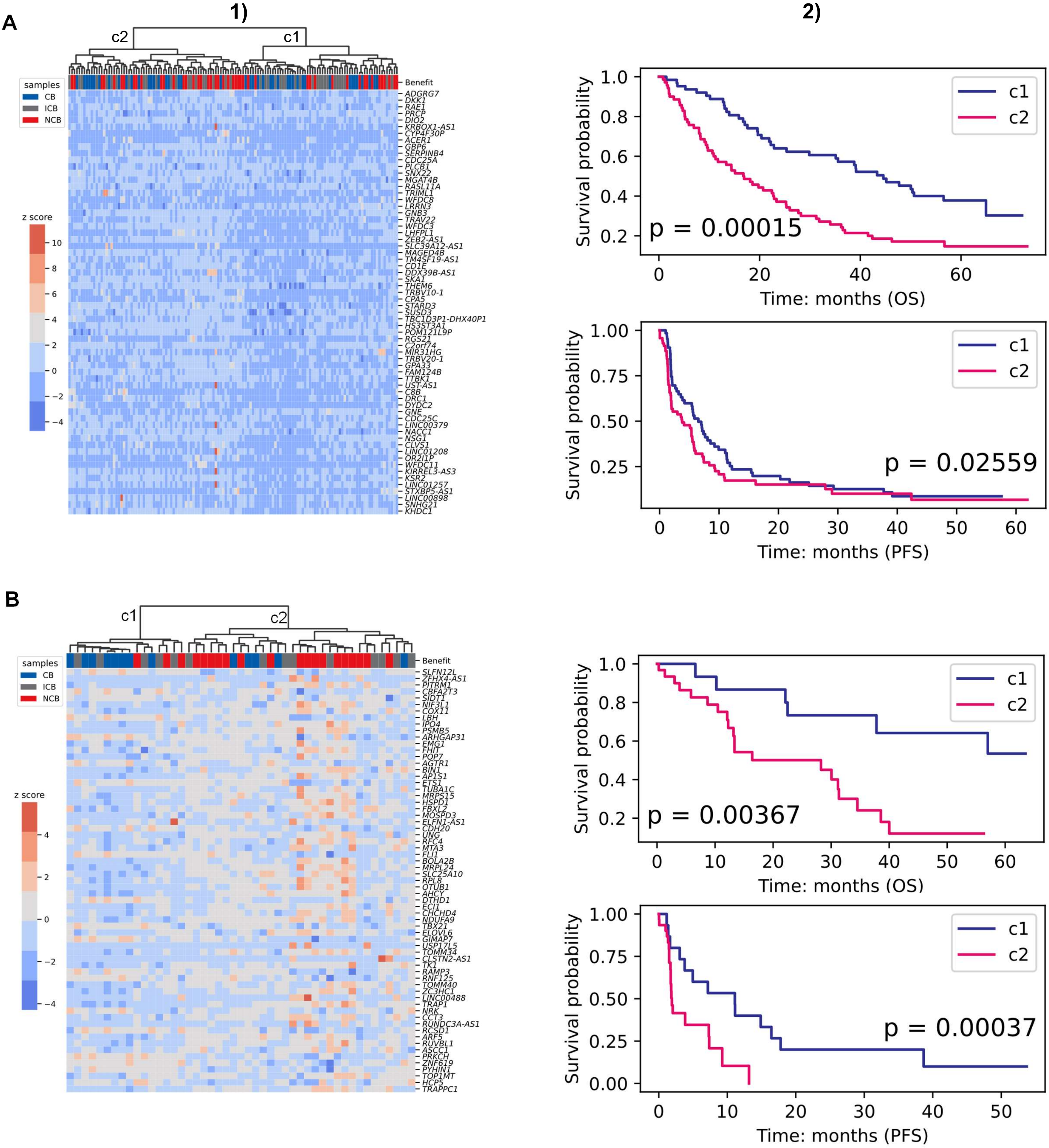

Figure S27. Comparison between two clusters generated from their gene expression values of pN (A) and mN (B), related to Figure 3 &4. (1). Hierarchy clustering of samples based on expression values of selected genes (64 genes of pN, 65 genes of mN). Genes were used if they were significantly related to both OS and PFS (the same method we used for gene connectivity). Two clusters (c1 and c2) were preferred. (2). Survival analysis between cluster c1 (blue) and c2 (pink). P values were from log rank tests.

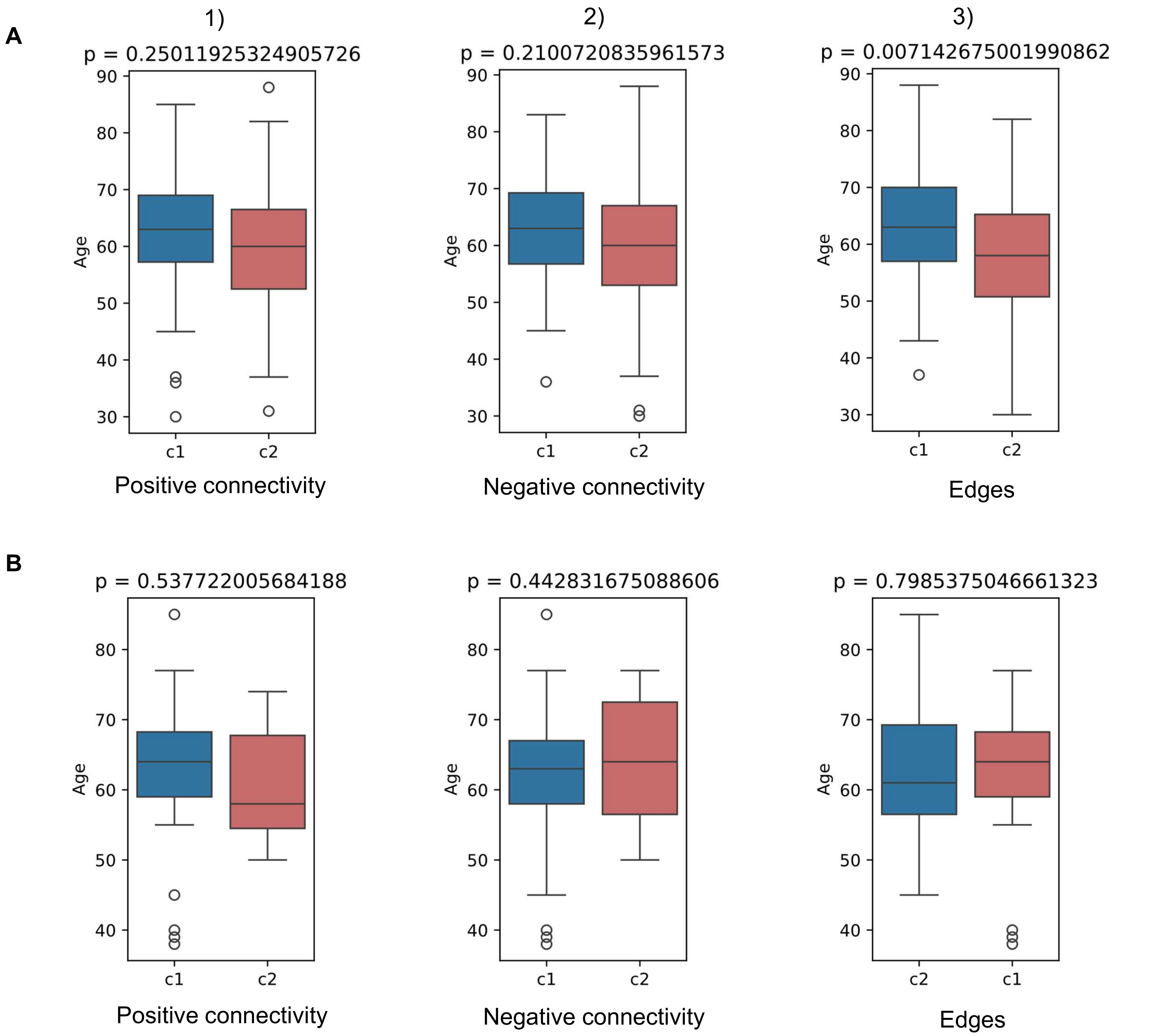

Figure S28. Comparison of age between two clusters generated from their gene connectivity of positive correlation 1) or negative correlation 2) or edges 3) of pN (A) and mN (B), related to Figure 6. P values were calculated from Student T tests.

Cancer	Localization	Treatment	No. of tumor samples
Advanced ccRCC	Primary	Nivolumab	133
Advanced ccRCC	Primary	Everolimus	92
Advanced ccRCC	Metastasis	Nivolumab	47
Advanced ccRCC	Metastasis	Everolimus	37
ccRCC	Unknown	Avelumab and Axitinib	354
Normal kidney tissue	Cortex	NA	85

Table S1. RNA-seq data from several studies, including the Braun 2020 paper, the Motzer 2020 paper, and the GTEx portal, related to Figure 1.

subcohort		Numbers of prognostic genes based on gene connectivity						
		OS	PFS	Overlapped		OS	PFS	Overlapped
pN	positive correlation	218	85	21	positive correlation	209	108	48
pE		74	97	29		114	131	39
mN		72	114	9		100	165	17
mE		69	17	1		67	11	0

Table S2. Number of prognostic genes based on gene connectivity, related to Figure 3.
Genes were selected by cox regression model and p-values at 0.01 was applied.

subcohort	Numbers of prognostic genes based on edges			
		OS	PFS	Overlapped
pN	edges	214	224	51
pE		228	165	40
mN		85	100	6
mE		70	84	10

Table S3.Number of prognostic genes based on edges, related to Figure 4.
Genes were selected by cox regression model and p-values at 0.01 was applied.

Query Name	Rank	TF	Score	Library	Overlapping_Genes
gene_set_query	1	MZF1	6	4 Coexpression,1;Enrichr Queries,9;GTEx Coexpr	PNPLA7,PNKP,KMT2B,SCRIB,PKD1,RHOT2,INF2,IRF3,ATG16L2,TTLL3,TAF1C,CROCC,SGSM3,TUBGCP6,TNRC18,CAMTA2,MAN2C1,CLASRP,RGL2,PPP1R12C
gene_set_query	2	ZNF692	9.667	Coexpression,6;Enrichr Queries,1;GTEx Coexpre	CENPT,PNPLA7,CROCCP2,KMT2B,SCRIB,PKD1,RHOT2,INF2,ATG16L2,TAF1C,CROCC,SGSM3,TUBGCP6,TNRC18,CLASRP,MAN2C1,RGL2,ARGLU1,PPP1R12C
gene_set_query	3	ZNF76	15.67	Coexpression,19;Enrichr Queries,25;GTEx Coexp	TCF25,PNKP,KMT2B,AKAP17A,PKD1,ABTB1,RHOT2,INF2,TTLL3,TAF1C,CROCC,SGSM3,TUBGCP6,TNRC18,CAMTA2,MAN2C1,CLASRP,RGL2,PPP1R12C,GAS8
gene_set_query	4	CXXC1	18.67	Coexpression,29;Enrichr Queries,17;GTEx Coexp	CENPT,KMT2B,SCRIB,AKAP17A,RHOT2,INF2,IRF3,TAF1C,CROCC,SGSM3,CDC37,TUBGCP6,CLASRP,MAN2C1,PPP1R12C
gene_set_query	5	ZNF316	20.33	Coexpression,24;Enrichr Queries,12;GTEx Coexp	TRADD,PNKP,KMT2B,SCRIB,AKAP17A,PKD1,INF2,IRF3,ATG16L2,TAF1C,CROCC,TUBGCP6,TNRC18,CLASRP,MAN2C1,RGL2,PPP1R12C,SKP1
gene_set_query	6	RBCK1	21	ARCHS4 Coexpression,35;GTEx Coexpression,7	CCDC142,CROCCP2,PNKP,AKAP17A,ABTB1,RHOT2,INF2,IRF3,ATG16L2,SGSM3,TUBGCP6,TNRC18,CLASRP,RGL2,PPP1R12C
gene_set_query	7	PRR12	23.33	Coexpression,27;Enrichr Queries,11;GTEx Coexp	UNC45A,CENPT,SETD1B,KMT2B,SCRIB,PKD1,RHOT2,INF2,CROCC,TUBGCP6,TNRC18,CAMTA2,CLASRP,PPP1R12C
gene_set_query	8	SCX	25	ARCHS4 Coexpression,25	RHOT2,INF2,TAF1C,KMT2B,SCRIB,TNRC18,CLASRP,PPP1R12C,RGL2
gene_set_query	9	MBD6	29.5	ARCHS4 Coexpression,38;GTEx Coexpression,21	TCF25,PNKP,SETD1B,KMT2B,AKAP17A,ABTB1,RHOT2,ATG16L2,TUBGCP6,TNRC18,MAN2C1,CLASRP,RGL2,PPP1R12C
gene_set_query	10	ZNF783	31	Coexpression,46;Enrichr Queries,29;GTEx Coexp	SETD1B,KMT2B,SCRIB,AKAP17A,RHOT2,INF2,IRF3,TTLL3,ATG16L2,TAF1C,CROCC,TUBGCP6,TNRC18,MAN2C1,CLASRP,NLRP1,ARGLU1
gene_set_query	11	SAFB2	33.5	ARCHS4 Coexpression,4;GTEx Coexpression,63	TCF25,KMT2B,AKAP17A,PKD1,RHOT2,TTLL3,TAF1C,CROCC,TUBGCP6,TNRC18,CLASRP,MAN2C1,NLRP1,PPP1R12C
gene_set_query	12	ANKZF1	35.67	Coexpression,8;Enrichr Queries,95;GTEx Coexp	CENPT,TCF25,AKAP17A,ABTB1,RHOT2,IRF3,ATG16L2,TTLL3,TAF1C,CROCC,SGSM3,TUBGCP6,CLASRP,MAN2C1,NLRP1,ARGLU1,RGL2
gene_set_query	13	FLYWCH1	43	ARCHS4 Coexpression,3;GTEx Coexpression,83	KMT2B,SCRIB,PKD1,RHOT2,INF2,TAF1C,CROCC,TUBGCP6,TNRC18,CAMTA2,MAN2C1,NLRP1,CLASRP,ARGLU1,PPP1R12C
gene_set_query	14	E4F1	51.5	63;ARCHS4 Coexpression,21;Enrichr Queries,3;G	CENPT,PNKP,KMT2B,SCRIB,PKD1,RHOT2,IRF3,TAF1C,CROCC,TUBGCP6,TNRC18,CAMTA2,CLASRP,MAN2C1,ERGIC2,RGL2,PPP1R12C,ARGLU1
gene_set_query	15	IRF3	52.6	ODE ChIP-seq,106;Enrichr Queries,71;ReMap Ch	USP16,TRADD,PNKP,KMT2B,TMTC3,AKAP17A,RHOT2,TAF1C,MAN2C1,NLRP1,RBM15,BBIP1,<br>COMMD6,ABTB1,RPS27,ATG16L2,TTLL3,IRF3,EIF3L,SGSM3,CDC37,TUBGCP6,CLASRP,RGL2,PPP1R12C,RPS24
gene_set_query	16	CC2D1A	54	ARCHS4 Coexpression,9;GTEx Coexpression,99	CENPT,KMT2B,SCRIB,PKD1,RHOT2,INF2,TAF1C,CROCC,TUBGCP6,TNRC18,CAMTA2,MAN2C1,CLASRP,ERGIC2,PPP1R12C
gene_set_query	17	ATF6B	54	ARCHS4 Coexpression,31;GTEx Coexpression,77	TRADD,PNKP,KMT2B,AKAP17A,ABTB1,IRF3,ATG16L2,TUBGCP6,TNRC18,MAN2C1,CLASRP,RGL2,PPP1R12C
gene_set_query	18	HSF4	61.67	Coexpression,2;Enrichr Queries,171;GTEx Coexp	CENPT,PNPLA7,KMT2B,SCRIB,PKD1,ABTB1,RHOT2,INF2,TAF1C,CROCC,SGSM3,TUBGCP6,TNRC18,CAMTA2,MAN2C1,CLASRP,PPP1R12C,GAS8
gene_set_query	19	KMT2B	71.5	ARCHS4 Coexpression,18;ReMap ChIP-seq,125	CENPT,ZNF770,RBM15,USP16,CCDC142,SETD1B,KMT2B,SCRIB,RHOT2,RPS27,INF2,TAF1C,TUBGCP6,TNRC18,MAN2C1,CLASRP,CBLL1,PPP1R12C

Table S4. The enrichment of transcription factor was obtained from online query on the website: https://maayanlab.cloud/chea3/#top, related to Figure 4.

51 genes from the intersection of significant OS and PFS edges in pN were used as the input. We have added TFEA.ChiP (https://www.iib.uam.es/TFEA.ChIP/) as an alternative tool to infer transcription factors, and genes MZF1, ZNF692 were also detected.

Feature	Score	loocv
RPL9_SGSM3	23.69543	0
ACAP3_RPL26	19.26899	0
CCDC6_INF2	18.25947	0
MTMR9	17.99828	0
PRELID3B_TTLL3	17.066	0
FCHSD1_RPF2	16.25079	0
MAPK8IP3_PTPRA	16.01615	0
FAAH_MOB1A	15.86933	0
NCKAP1_NLRP1	15.86933	0
ABI3	15.81455	0
ATG16L2_NDUFA6	15.643	0
PIP5K1C	14.76414	0
ATG2A_RPS24	14.73009	0
ZNF578	14.64298	0
RHPN1_RPL32	14.55772	0
RGL2_TMED7	14.3592	0
CENPT_RPS24	14.23818	0
CROCC_PRKAG1	14.23818	0
INF2_OGFRL1	13.85264	0
AKAP17A_ZNF770	13.85264	0
MAN2C1_OGFRL1	13.77035	0
PRKAG1_RGL2	13.693	0
CSNK2B	13.64308	0
AS3MT	13.46555	0
GAS8_RPL30	13.23404	0
STXBP5-AS1	12.91943	0
ATG16L2_SUCO	12.89933	0
AKAP14	12.76576	0
NDUFA6_RGL2	12.57806	0
INF2_POLR2M	12.40518	0
TUBGCP6_UBXN2B	12.40518	0
ACADVL_RPL23A	12.3999	0
SF3B3	12.12962	0
EXOC5_RGL2	12.02602	0
ABCA12	11.9675	0
RPS24_SH3GLB2	11.93392	0
UBALD2	11.93033	0
CCNL2_NPM1	11.92258	0
CCDC142_RFC1	11.92258	0
ERGIC2_MBD1	11.65323	0
AP3M1_TTLL3	11.64944	0
SNRPB2_TBC1D9B	11.52755	0
OR2W3	11.46306	0
LRCH4_UBA2	11.41776	0
ATG2A_NSA2	11.38486	0
RPL11_SGSM2	11.23836	0
MBD1_UQCRB	11.17529	0
NACA_TCF25	11.16506	0
ERGIC2_TCF25	11.09826	0
NRIP1_TRADD	11.07967	0

Table S5. Feature selection (selectKbest, n=50) in LOOCV, with the combination of gene expression and edges as the input, related to Figure 6.

Feature	Model	Feature Number	Accuracy	Precision	F1	tn	tp	fp	fn	sensitivity	specificity
Expression		10	0.7	0.697674	0.689655	30	33	14	13	0.717391304	0.681818182
Positive-connectivity		90	0.622222	0.638889	0.575	23	33	21	13	0.717391304	0.522727273
Negative-connectivity		30	0.555556	0.541667	0.565217	26	24	18	22	0.52173913	0.590909091
Edge		50	0.733333	0.7	0.744681	35	31	9	15	0.673913043	**0.795454545**
Expression+Positive-connectivity	LR	10	0.644444	0.636364	0.636364	28	30	16	16	0.652173913	0.636363636
Expression+Negative-connectivity		10	0.711111	0.714286	0.697674	30	34	14	12	0.739130435	0.681818182
Expression+Edge		50	**0.75556**	**0.75**	**0.75**	33	35	11	11	**0.760869565**	0.75
GSVA		10	0.5	0.488372	0.482759	21	24	23	22	0.52173913	0.477272727
Entropy		10	0.588889	0.594595	0.54321	22	31	22	15	0.673913043	0.5
GSVA+Entropy		40	0.511111	0.5	0.488372	21	25	23	21	0.543478261	0.477272727
Expression		10	0.688889	0.681818	0.681818	30	32	14	14	0.695652174	0.681818182
Positive-connectivity		80	0.577778	0.575	0.547619	23	29	21	17	0.630434783	0.522727273
Negative-connectivity		10	0.566667	0.586207	0.465753	17	34	27	12	**0.739130435**	0.386363636
Edge		60	0.666667	0.666667	0.651163	28	32	16	14	0.695652174	0.636363636
Expression+Positive-connectivity	SVM	10	0.566667	0.555556	0.561798	25	26	19	20	0.565217391	0.568181818
Expression+Negative-connectivity		10	0.677778	0.682927	0.658824	28	33	16	13	0.717391304	0.636363636
Expression+Edge		100	**0.7**	**0.68889**	**0.69663**	31	32	13	14	0.695652174	**0.704545455**
GSVA		10	0.533333	0.517241	0.588235	30	18	14	28	0.391304348	0.681818182
Entropy		10	0.588889	0.612903	0.506667	19	34	25	12	**0.739130435**	0.431818182
GSVA+Entropy		60	0.533333	0.522727	0.522727	23	25	21	21	0.543478261	0.522727273
Expression		10	**0.76667**	**0.76744**	**0.75862**	33	36	11	10	**0.782608696**	**0.75**
Positive-connectivity		30	0.555556	0.55	0.52381	22	28	22	18	0.608695652	0.5
Negative-connectivity		20	0.555556	0.547619	0.534884	23	27	21	19	0.586956522	0.522727273
Edge		60	0.577778	0.555556	0.612245	30	22	14	24	0.47826087	0.681818182
Expression+Positive-connectivity	RF	10	0.544444	0.540541	0.493827	20	29	24	17	0.630434783	0.454545455
Expression+Negative-connectivity		10	0.633333	0.648649	0.592593	24	33	20	13	0.717391304	0.545454545
Expression+Edge		20	0.688889	0.690476	0.674419	29	33	15	13	0.717391304	0.659090909
GSVA		10	0.511111	0.5	0.488372	21	25	23	21	0.543478261	0.477272727
Entropy		40	0.622222	0.625	0.595238	25	31	19	15	0.673913043	0.568181818
GSVA+Entropy		40	0.588889	0.577778	0.58427	26	27	18	19	0.586956522	0.590909091
Expression		10	**0.73333**	**0.75**	0.714286	30	36	14	10	**0.782608696**	0.681818182
Positive-connectivity		50	0.633333	0.648649	0.592593	24	33	20	13	0.717391304	0.545454545
Negative-connectivity		20	0.566667	0.553191	0.571429	26	25	18	21	0.543478261	0.590909091
Edge		50	0.677778	0.653061	0.688172	32	29	12	17	0.630434783	0.727272727
Expression+Positive-connectivity	DNN	70	0.633333	0.627907	0.62069	27	30	17	16	0.652173913	0.613636364
Expression+Negative-connectivity		10	0.711111	0.714286	0.697674	30	34	14	12	0.739130435	0.681818182
Expression+Edge		90	**0.73333**	0.708333	**0.73913**	34	32	10	14	0.695652174	**0.772727273**
GSVA		10	0.5	0.487805	0.470588	20	25	24	21	0.543478261	0.454545455
Entropy		30	0.511111	0.5	0.521739	24	22	20	24	0.47826087	0.545454545
GSVA+Entropy		50	0.5	0.487805	0.470588	20	25	24	21	0.543478261	0.454545455

Table S6. The performance metric of ML models (default parameter with class weight assigned to "balanced"), with the inputs as gene expression, network features, and their combinations, related to Figure 6.

	Model	Feature Number	Accuracy	Precisior	F1	tn	tp	fp	fn	sensitivity	specificity
Expression	LR	10	0.711111	**0.70455**	0.704545	31	33	13	13	0.717391304	0.704545455
Positive-connectiv		90	0.6	0.605263	0.560976	23	31	21	15	0.673913043	0.522727273
Negative-connectiv		30	0.544444	0.533333	0.539326	24	25	20	21	0.543478261	0.545454545
Edge		50	0.688889	0.666667	0.695652	32	30	12	16	0.652173913	0.727272727
Expression+Positiv		10	0.6	0.595238	0.581395	25	29	19	17	0.630434783	0.568181818
Expression+Negativ		10	0.7	0.707317	0.682353	29	34	15	12	**0.739130435**	0.659090909
Expression+Edge		50	**0.72222**	0.702128	**0.72527**	33	32	11	14	0.695652174	**0.75**
GSVA		10	0.466667	0.454545	0.454545	20	22	24	24	0.47826087	0.454545455
Entropy		10	0.555556	0.558824	0.487179	19	31	25	15	0.673913043	0.431818182
GSVA+Entropy		40	0.455556	0.444444	0.449438	20	21	24	25	0.456521739	0.454545455
Expression	SVM	10	**0.71111**	**0.69565**	**0.71111**	32	32	12	14	0.695652174	**0.727272727**
Positive-connectiv		80	0.588889	0.574468	0.593407	27	26	17	20	0.565217391	0.613636364
Negative-connectiv		10	0.533333	0.529412	0.461538	18	30	26	16	0.652173913	0.409090909
Edge		60	0.655556	0.638298	0.659341	30	29	14	17	0.630434783	0.681818182
Expression+Positiv		10	0.588889	0.594595	0.54321	22	31	22	15	0.673913043	0.5
Expression+Negativ		10	0.666667	0.675	0.642857	27	33	17	13	**0.717391304**	0.613636364
Expression+Edge		100	0.688889	0.673913	0.688889	31	31	13	15	0.673913043	0.704545455
GSVA		10	0.5	0.494382	0.661654	44	1	0	45	0.02173913	1
Entropy		10	0.577778	0.59375	0.5	19	33	25	13	**0.717391304**	0.431818182
GSVA+Entropy		60	0.5	0.490566	0.536082	26	19	18	27	0.413043478	0.590909091
Expression	RF	10	**0.73333**	**0.71739**	**0.73333**	33	33	11	13	**0.717391304**	**0.75**
Positive-connectiv		30	0.533333	0.527778	0.475	19	29	25	17	0.630434783	0.431818182
Negative-connectiv		20	0.544444	0.538462	0.506024	21	28	23	18	0.608695652	0.477272727
Edge		60	0.555556	0.54	0.574468	27	23	17	23	0.5	0.613636364
Expression+Positiv		10	0.566667	0.567568	0.518519	21	30	23	16	0.652173913	0.477272727
Expression+Negativ		10	0.633333	0.648649	0.592593	24	33	20	13	**0.717391304**	0.545454545
Expression+Edge		20	0.722222	0.711111	0.719101	32	33	12	13	**0.717391304**	0.727272727
GSVA		10	0.555556	0.547619	0.534884	23	27	21	19	0.586956522	0.522727273
Entropy		40	0.588889	0.589744	0.554217	23	30	21	16	0.652173913	0.522727273
GSVA+Entropy		40	0.555556	0.55	0.52381	22	28	22	18	0.608695652	0.5
Expression	DNN	10	0.577778	**0.63636**	0.424242	14	38	30	8	**0.826086957**	0.318181818
Positive-connectiv		50	0.577778	0.575	0.547619	23	29	21	17	0.630434783	0.522727273
Negative-connectiv		20	0.444444	0.425	0.404762	17	23	27	23	0.5	0.386363636
Edge		50	**0.62222**	0.631579	0.585366	24	32	20	14	0.695652174	0.545454545
Expression+Positiv		70	0.466667	0.444444	0.4	16	26	28	20	0.565217391	0.363636364
Expression+Negativ		10	0.611111	0.68	0.492754	17	38	27	8	**0.826086957**	0.386363636
Expression+Edge		90	0.522222	0.513514	0.469136	19	28	25	18	0.608695652	0.431818182
GSVA		100	0.5	0.492063	0.579439	31	14	13	32	0.304347826	**0.704545455**
Entropy		30	0.611111	0.621622	0.567901	23	32	21	14	0.695652174	0.522727273
GSVA+Entropy		50	0.611111	0.604651	**0.5977**	26	29	18	17	0.630434783	0.590909091

Table S7. The performance metric of ML models, with hyperparameter tuning, related to Figure 6.

The inputs as gene expression, network features, and their combinations. Hyperparameter range can be found in Table S8.

ML model	Hyperparameter type	Grid
LR	C	0.1, 0.2, 0.3, 0.4, 0.5, 0.6, 0.7, 0.8, 0.9, 1
SVM	C	0.1, 0.2, 0.3, 0.4, 0.5, 0.6, 0.7, 0.8, 0.9, 1
	kernel	linear', 'rbf'
	gamma	scale', 'auto'
RF	rf__n_estimators	100, 150
	max_depth	None,3,
	min_samples_split	2, 5
	min_samples_leaf	1, 2
DNN	hidden_layer_sizes	(50,), (100,), (50, 50), (256, 256),(128,128,128)
	alpha	0.0001, 0.001, 0.01
	learning_rate	constant', 'adaptive'
	max_iter	10, 50, 100, 200

Table S8. The hyperparameter grid used for Machine-learning models, related to Figure 6.
